# Single-parameter programmed thermomechanical actuation via 3D-printed helical director fields in liquid crystal elastomers

**DOI:** 10.1038/s41467-026-73204-y

**Published:** 2026-05-15

**Authors:** Yuxuan Sun, Boxi Sun, Zhengqing Zhu, Jiyang Wu, Hao Jing, Xingxiang Li, Dongxiao Li, Ziyi Zhang, Dongchang Zheng, Guorui Wang, Weihua Li, Yu Xiao, Tingrui Pan, Yong Chen, Shiwu Zhang, Mujun Li

**Affiliations:** 1 Institute of Humanoid Robots, Department of Precision Machinery and Precision Instrumentation, University of Science and Technology of China, Hefei, China; 2 Department of Modern Mechanics, University of Science and Technology of China, Hefei, China; 3 School of Mechanical, Materials, Mechatronic and Biomedical Engineering, University of Wollongong, Wollongong, NSW Australia; 4 Suzhou Institute for Advanced Research, University of Science and Technology of China, Suzhou, China; 5 Epstein Department of Industrial and Systems Engineering, Viterbi School of Engineering, University of Southern California, Los Angeles, CA USA; 6 Department of Mechanical and Aerospace Engineering, The Hong Kong University of Science and Technology, Hong Kong, China

**Keywords:** Polymers, Liquid crystals, Liquid crystals

## Abstract

Stimuli-responsive material like liquid crystal elastomers (LCEs) hold great promise for untethered soft machines, yet conventional extrusion-based 3D printing restricts their molecular alignment strictly to the uniaxial deposition path. This inherent constraint strongly couples the actuation mode to the printed geometry, typically requiring complex multi-material architectures or spatially structured stimuli to achieve multimodal behaviors. Here we introduce a rotational 3D printing approach that embeds a helical director field within LCE filaments, enabling multimodal actuation controlled by a single fabrication parameter: the helix angle (*θ*). Tuning *θ* programs each filament to contract, elongate, twist or remain macroscopically invariant when heated, decoupling actuation from device geometry. Spatial gradients in *θ* create a hierarchy of activation temperatures, yielding sequential shape changes under uniform heating. Localized heating of the magnetic-LCE composite segments allows their magnetic domains to be reoriented, making the shape programs rewritable and enabling switchable volatile and non-volatile memory. We demonstrate these capabilities in self-partitioning grippers, multimodal/color robots and reprogrammable guidewires that perform multi-step or adaptive tasks without external circuitry. By encoding actuation modes, deformation sequences, and memory in a single parameter, this approach establishes a paradigm of material-encoded programmability and points toward monolithic soft robots and reconfigurable structures.

## Abstract (web summary)

Liquid crystal elastomers are promising for actuators in soft robotics, but 3Dprinting methods often restrict responsive properties. Here the authors design by tuning the printing angle, affording a coiled fiber, for programmable actuation.

## Introduction

Stimuli-responsive materials provide a pathway to soft machines capable of complex, untethered shape transformations. By leveraging intrinsic thermomechanical properties rather than external motors, these systems can achieve sophisticated behaviors such as self-folding, locomotion, and adaptive gripping^1–5^. Rather than relying solely on external computation or rigid control algorithms, stimuli-responsive systems leverage material architectures to realize behaviors such as self-shaping^6–10^, self-sensing^11–13^, and adaptive response^14–16^ in situ. A key challenge in this paradigm is creating actuators that can exhibit desired modes of behavior, enabling machines with multi-modal, adaptive and even reconfigurable response^2,3,17^. Recent advances in smart materials have started to address this: for example, liquid crystal elastomers (LCEs) can be programmed to undergo large, predetermined shape changes (e.g., contraction, bending, or twisting) when stimulated by heat or light^18–20^. By encoding molecular alignment within an LCE, researchers can impart a form of encoded anisotropy—the material itself directs how it will move under stimuli, contributing to the system’s overall functionality^6–9,14,15^. However, traditional programming methods are typically tailored for simple deformation mode (e.g., mechanical stretching^21–23^), limited to thin-film LCEs (e.g., surface patterning^24–26^), or limited complexity and materials (electrical/magnetic field alignment^19,27,28^). Creating devices that can achieve complex actuation modes usually requires combining separate actuators or complex architectures, adding design and fabrication complexity that run counter to the concept of monolithic, integrated design.

3D printing has emerged as a powerful approach to fabricate custom-aligned LCE actuators with fine control over the director field (the orientation of liquid crystal molecules) within printed structures. Pioneering work by the Lewis^8,11,29^, Qi^30,31^, and Ware^32,33^ groups demonstrated that extrusion-based deposition can align LCE molecules via shear-induced orientation. For example, Kotikian et al. introduced a multi-material 3D printing strategy in which shear from the extruder aligns LCE filaments along the printing axis (**n**//**l**). This alignment enabled printed LCE robots to exhibit continuous rolling motion under a temperature gradient^8^. However, conventional extrusion-based 3D printing inherently limits the director field to uniaxial alignment along the deposition path, enforcing a rigid one-dimensional programming paradigm^34–37^. This constraint largely fixes the filament-level programmed eigenstrain to a dominant axial response along the deposition direction, such that the intrinsic actuation palette of a monolithic extruded filament remains effectively one-dimensional; while sophisticated shape changes (e.g., planar folding^8,30^, arching^38,39^, and twisting^37,40^) and even richer, non-developable curvatures (including Gaussian curvature) can be achieved via antagonistic/interface designs, voxelation/multi-material architectures, and/or spatially patterned stimuli, these approaches typically add geometric/toolpath or stimulus-field complexity. As a result, the printed LCE structures exhibit strong coupling between deformation mode and printing path, complicating independent control of distinct deformation dimensions. Recent efforts have explored multi-material printing^41–43^ and post-processing alignment^22^ to address these issues. While such composite approaches can yield multifunctional devices, they increase design complexity, and post-processing steps that may reduce robustness and scalability. Multimodal deformations in a monolithic LCE can also be achieved via spatially structured stimuli, for example locally addressed photo-^44^, magneto-^45^, or spatially programmed temperature fields. However, the resulting mode is inherently tied to the spatiotemporal stimulus field and may be constrained by practical requirements such as line-of-sight/penetration, spatial calibration, and thermal management, particularly for thick or embedded structures. Ideally, a single monolithic LCE element that can be programmed with any desired deformation mode in situ would offer greater adaptability, simplifying the creation of responsive machines with rich behavior repertoires.

Recently, rotational printing has been proposed for increasing mechanical performance and programming the sub-filament architectures. For example, Raney et al. demonstrated that rotating the nozzle during DIW could impart helical alignment to short fibers within a composite matrix, enhancing damage tolerance via crack steering^46^. Larson et al. further advanced this technique to rotational multimaterial printing, achieving sub-voxel control over filament microstructure to program anisotropic stiffness and swelling behaviors in hydrogels and epoxies^47^. Similarly, Ren et al. leveraged twisted interfaces in bi-material systems to generate mismatch strain for deformation or suture-like geometries for mechanical interlocking^48,49^. However, these strategies rely on structuring heterogeneous phases—either by aligning discontinuous fillers or manipulating material distribution. The potential of this kinematic strategy to program the intrinsic molecular director field within a monolithic filament for multimodal actuation remains underexplored. Leveraging nozzle rotation to decouple the actuation mode from the printing path in a single material could significantly expand the design space of soft structures and robotics, yet a systematic methodology for such single-parameter programming is still lacking (Supplementary Table 1).

Here, we introduce a rotational 3D printing method that creates programmable helical director fields within individual LCE filaments. This approach allows a single LCE material to exhibit multiple actuation modes by tuning a single parameter during fabrication. In this method, a filament of LCE ink is extruded while the printer nozzle continuously rotates, encoding a helical twist in the molecular alignment (director) along the filament’s length. The helix angle *θ* of this director field is set solely by the ratio of nozzle rotation speed to translation speed, and this angle serves as a single design parameter that deterministically governs how the filament will deform when heated. By simply adjusting *θ*, we can program a filament to contract (*θ* = 0°), torsionally twist (at a neutral angle *θ*_*n*_ = 68°), elongate (*θ* = alternating ±80°), or even remain unchanged (*θ* = alternating ±68°) when heated. Moreover, spatially varying *θ* along the filament during printing produces combined responses (e.g., simultaneous twist and contraction), allowing a continuum of deformation modes to be achieved within the same printed material. This single-parameter programmability effectively decouples the actuation mode from the physical printing path or device geometry. Consequently, an LCE filament can be laid out in an arbitrary shape or integrated as a component of a larger device, while its actuation behavior is independently determined by the chosen director helix angle. It also enables sequential actuation via spatially graded *θ*, wherein a uniform magnetic field triggers different segments in a prescribed order. Finally, via magnetic reprogramming, the system can undergo both volatile and non-volatile deformations—either reverting to its original shape once the field is removed or retaining a new permanent shape.

## Results

### Rotational printing enabled helix director field

**Fig. 1:**
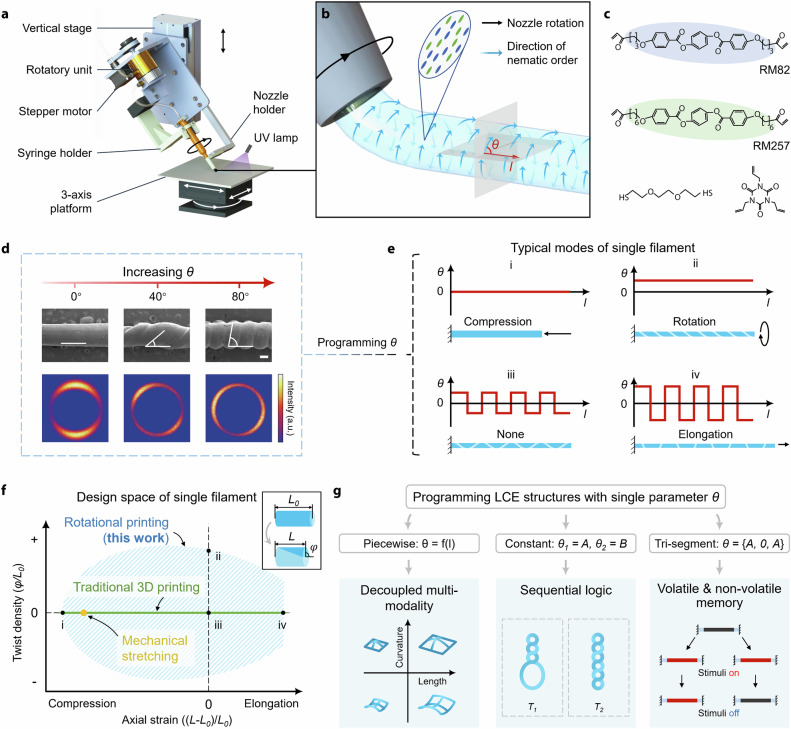
Helix-aligned LCE filament printing system and resulting director orientation and actuation modes. **a** Schematic of the 3D printing setup, featuring a two-axis translation stage for nozzle positioning, a rotating build platform, and a rotational printhead (with syringe and nozzle) driven by a stepper motor. **b** Illustration of a printed filament showing helical alignment of the LCE director due to nozzle rotation; the director field (**n**) rotates from the core to the periphery, and the angle between the outer-layer director and the filament axis (printing direction, **l**) is defined as *θ*. **c** Chemical constituents of the LCE ink. **d** Wide-angle X-ray scattering (WAXS) characterization of filament alignment, indicating that the director helix angle *θ* increases with higher nozzle rotation speed *ω*. (scale bar = 0.1 mm) (**e**) Demonstration of four fundamental thermomechanical deformation modes obtained by programming *θ* along the filament: axial contraction (0°), near-pure twist (68°), axial elongation ( ± 80°), and macroscopic invariance ( ± 68°). **f** Two-dimensional design space spanned by axial strain and twist density, illustrating the expansion from conventional one-dimensional programming to coupled strain–twist control, where points i–iv correspond to the four deformation modes shown in (**e**). **g** Multi-modal actuation, time-programmed sequential actuation, and switchable volatile / non-volatile shape memory enabled by different programming forms of *θ*.

The printing system consists of a two-axis translation stage for positioning, a rotating platform, and a custom rotational printhead (Fig. 1a, Supplementary Fig. 1). The printhead assembly comprises a stepper motor, a rotary union, a syringe holder, and a syringe with an attached nozzle. The rotary union is mounted at a slight angle to provide a continuous pressurized ink channel while permitting relative rotation of the syringe. During printing, the stepper motor drives the syringe (and thus the nozzle) via a belt-and-pulley transmission, imparting a controlled angular speed *ω*. The nozzle is securely mounted on the robotic arm such that it rotates about a fixed vertical axis (Supplementary Fig. 2, Supplementary Movie 1).

Nozzle rotation during extrusion induces a helical alignment of the LCEs within the filament (Fig. 1b). In this study, we employed a main-chain LCE (Fig. 1c, Supplementary Fig. 3) with a molecular weight of *M*_*w*_ = 1.4×10^4^ (Supplementary Note 1, Supplementary Fig. 4). Notably, *T*_*NI*_ of this material can be tuned by adjusting the ratio of RM257 to RM82 (Supplementary Fig. 5). The LCE’s director field (denoted **n**) twists around the filament axis as the material exits the rotating nozzle. Here, we define the helix angle *θ* as the angle between the director in the filament’s outer layer and the printing direction (filament axis **l**). As such, *θ* is dictated by the ratio of nozzle angular velocity *ω* to translational print speed *v*. Under ideal kinematic conditions, this relationship is given by:

1 $$\theta=\arctan \left(\frac{\omega r}{v}\right)$$

where *r* is the nozzle radius. Here, *v* and *r* are fixed, so *θ* varies linearly with *ω*; *ω* therefore serves as a single design variable that deterministically governs how a filament will deform when heated. In the absence of nozzle rotation, the mesogens align predominantly along the print direction (*θ* = 0°). With nozzle rotation, the mesogens in the filament are reoriented to an oblique angle *θ* relative to the print path. By adjusting the angular velocity *ω*, the mesogen orientation can thus be continuously tuned from axial to helical. Wide-angle X-ray scattering (WAXS) measurements showing that *θ* increases proportionally with *ω* (Fig. 1d). To verify the director across the filament cross-section, we performed polarized Raman measurements on transverse cuts and extracted the local director angle *θ*(*r*) from radial line scans (Supplementary Fig. 6). The results show predominantly tangential (circumferential) mesogen alignment in the cross-section, with increasing anisotropy as the programmed helix angle *θ* increases, consistent with nozzle-rotation-induced director programming.

The programmed helix angle *θ* directly determines the filament’s deformation mode upon heating. We developed a theoretical calculation paradigm to predict the deformation of printed filament (Supplementary Note 2, Supplementary Fig. 7). While filament diameter—controlled by printing speed at a constant flow rate (Supplementary Fig. 8)—is a critical factor influencing thermal deformation (Supplementary Fig. 9), we maintained a constant diameter in this study; consequently, *θ* serves as the primary variable governing thermal response. As shown in Fig. 1e, filaments printed with *θ* = 0° (director aligned with the filament axis) undergo uniform axial contraction when heated. Guided by our analytical energy-minimization model (Supplementary Note 3), we theoretically predicted and experimentally verified a critical neutral helix angle (*θ*_*n*_ = 68°) that exhibits primarily torsional twisting with minimal length change (a near neutral length behavior). In contrast, filaments composed of alternating segments with opposing director orientations can achieve more complex actuation (Supplementary Note 4). For example, a filament patterned with alternating *θ* = +80° and *θ* = –80° segments will elongate upon heating, while alternating *θ* = +68° and *θ* = –68°segments result in macroscopic invariance. These four basic deformation modes—contraction, twisting, elongation, and macroscopic invariance —can be combined or spatially programmed by varying *θ* along the filament during printing. Because *θ* simultaneously controls axial strain and torsional rotation, rotational printing expands the design space from one to two dimensions (Fig. 1f). A single parameter thus can be programmed into different forms, enables sequential shape change, decoupled multimodal actuation, and reprogrammable volatile and non-volatile actuation behaviors in soft robotic systems (Fig. 1g).

### Programming single filament actuation

**Fig. 2:**
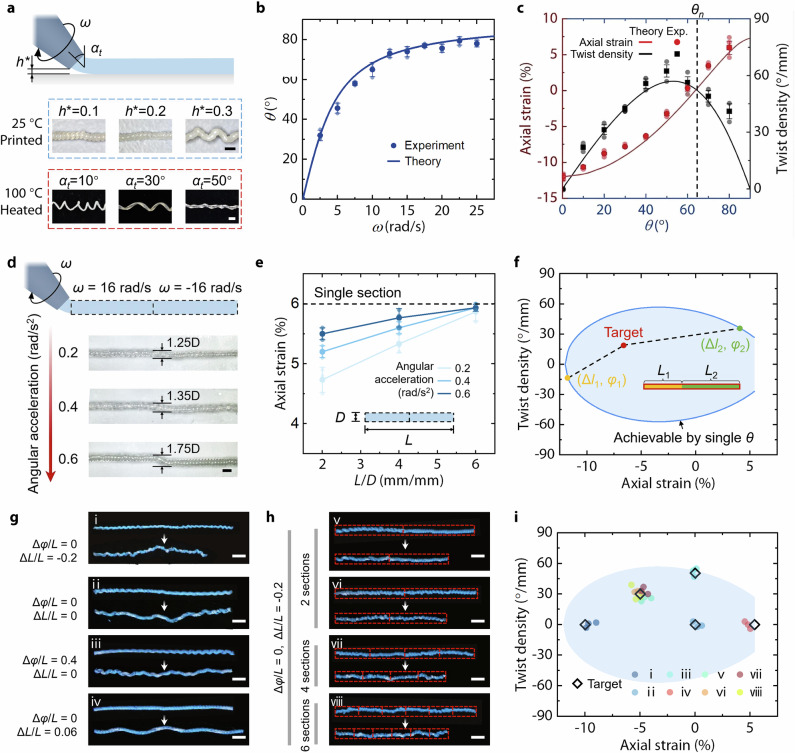
Single filament programming with multi-modality. **a** Comparison of filament morphologies printed at nozzle heights of *h*^∗^=0.1 mm, 0.2 mm and 0.3 mm, illustrating how overly low or high nozzle heights lead to excessive filament width or zigzag deposition. The bottom panels (100 °C) demonstrate the effect of nozzle tilt angle (*α*_*t*_) on actuation symmetry, showing that *α*_*t*_ = 50∘ yields stable helical deformation. (scale bar = 1 mm) (**b**) Schematic showing how the nozzle’s angular speed *ω* corresponds to the filament helix angle *θ*, which can be continuously varied from −80∘ to +80∘. **c** Influence of different *θ* values on the LCE filament’s twisting (right axis) and axial deformation (left axis) upon heating, transitioning from pure axial contraction to primarily twisting, and eventually to axial elongation. **d** Effect of different angular accelerations on the stability of the printed filament shape, where higher accelerations cause noticeable distortion. (scale bar = 0.5 mm) (**e**) Axial deformation under different acceleration. **f** The shaded region represents all achievable combinations of axial and torsional deformations (programmable deformation design space), the blue boundary marks the achievable deformation under uniform *θ*. **g** Selection of target deformations (contraction, macroscopically invariant, twisting, elongation) within the design space by dividing the filament into segments with different *θ*. (scale bar = 2 mm) (**h**) Printed samples demonstrating various target shapes achieved via different number of segments. **i** Experimental results show such multiple segmentations programming strategy can precisely program both axial and torsional deformations (Scale bar=5 mm). Data are presented as mean ± SD, *n* = 3 independent samples.

In our rotational 3D printing setup, the nozzle is tilted by an angle *α*_*t*_ relative to the build platform and positioned at a stand-off height *h* to ensure smooth deposition of LCE filaments without introducing significant asymmetry (which could lead to uncontrolled bending of the filament). Figure 2a demonstrates the effect of nozzle height: at h^*^ = 0.1 mm (very low nozzle height), the extruded filament over-extrudes slightly wider than the nozzle diameter; at *h* = 0.3 mm (too high), the deposited filament begins to meander on the substrate. An intermediate height of h^*^ = 0.2 mm produces a straight, smooth filament without distortion, and was therefore selected as the optimal nozzle height for reliable rotational printing. Printing with a non-tilted nozzle (*α*_*t*_ = 0°) causes significant shear between the nozzle tip and the fresh deposit, often yielding an asymmetric filament cross-section (Supplementary Fig. 10). Increasing *α*_*t*_ gradually alleviates this shear; by *α*_*t*_ = 50°, the filament can be laid down with minimal shear, resulting in a nearly symmetric, uniform filament that exhibits stable actuation without distortion upon heating (Fig. 2a). Under these low-shear conditions, the filament’s printed orientation angle *θ* is directly proportional to the nozzle’s angular speed *ω* (Fig. 2b), allowing *θ* to be tuned continuously from –80° to +80° by adjusting *ω*. We next characterize the thermally induced actuation of filaments printed with different *θ*. At *θ* = 0° (no twist imparted), the filament contracts along its length upon heating with no twisting. As *θ* increases from 0°, the filament’s actuation behavior gradually shifts: it begins to twist upon heating while its axial contraction diminishes. At a neutral angle *θ*_*n*_ of ~68°, the LCE filament exhibits pure macroscopic twisting upon heating, with zero net axial strain, indicating an almost pure torsional actuation. For *θ* beyond this value, the filament elongates when heated (instead of contracting) while still producing a significant twist (Fig. 2c, Supplementary Fig. 11). We also tested the temperature-dependence of deformation (Supplementary Fig. 12) and the repetition of deformation (Supplementary Fig. 13). In summary, by adjusting helix angle *θ*, we can program the filament’s actuation mode to range from purely axial deformation to purely torsional deformation, or a combination of both. Apart from thermal-induced deformation, adjusting *θ* can also program the mechanical property of printed filament. Specifically, as *θ* increases, the Young’s modulus gradually decreases from ~1.1 MPa *θ* = 0° to ~0.5 MPa at *θ* = 80° (Supplementary Fig. 14).

To program spatial variations of *θ* along a single filament—and thereby achieve more complex, decoupled deformation modes—we dynamically modulated the nozzle’s rotation during the printing process. In one demonstration (Fig. 2d), a filament was printed with two consecutive segments having opposite orientations ( + 80° followed by –80°). We varied the nozzle’s angular acceleration during the transition between these segments to observe its effect. With a high angular acceleration (∼0.6 rad/s^2^), the filament was noticeably distorted at the junction between the two sections (the sudden rotation caused a perturbation in the filament laydown). In contrast, using a more moderate acceleration ( ≤ 0.4 rad/s^2^) produced a filament that remained nearly straight through the transition region. However, if the acceleration is too low, the nozzle takes an excessively long time to reach the new target orientation, meaning a longer length of filament is printed under a gradual transition. This extended transition can blur the distinction between segments and alter the filament’s final actuation behavior (Fig. 2e). We found that an angular acceleration of about 0.4 rad/s^2^ provides the optimal balance: it is gentle enough to avoid distortion at the segment interface, yet fast enough to quickly achieve the desired *θ* change without overly prolonging the transition. Using this optimized acceleration, we can reliably program *θ* to change along the filament’s length, enabling custom combinations of axial and torsional actuation in a single printed filament.

With this approach established, a single LCE filament can be programmed to exhibit specific axial and twisting deformations by prescribing the helix angle *θ* as a function of position along its length. To illustrate the design possibilities, we first mapped out the actuation design space for filaments printed with a uniform *θ* (i.e., single-section filaments). The blue boundary in Fig. 2f delineates the range of axial strain (contraction or extension, Δ*l*) and twist (rotation angle *φ*) that such filaments can achieve upon heating, as *θ* is varied from –80° to +80°. Within this boundary lies the continuum of achievable actuation combinations for a uniformly oriented filament.

Consider a desired target deformation consisting of a certain axial change Δ*L*_*t*_ and twist *φ*_*t*_ that falls inside this design space. In principle, any such target point can be achieved by dividing the filament into two or more segments with different print orientations. For example, a two-segment filament with respective print angles *θ*_*1*_ and *θ*_*2*_ (and segment lengths *L₁* and *L₂*) can be designed so that its overall twist and axial actuation match the target. Serving as an inverse-design tool to eliminate empirical trial-and-error, our theoretical framework directly dictates the required segment lengths *L₁* and *L₂* of each section determined by (Supplementary Note 4):

2 $$\left\{\begin{array}{c}{L}_{1}=\frac{{\varphi }_{2}{\Delta l}_{{{{\rm{t}}}}}-{\varphi }_{{{{\rm{t}}}}}{\Delta l}_{2}}{{\varphi }_{2}{\Delta l}_{1}-{\varphi }_{1}{\Delta l}_{2}}L\\ {L}_{2}=\frac{{\varphi }_{1}\Delta {l}_{{{{\rm{t}}}}}-{\varphi }_{{{{\rm{t}}}}}{\Delta l}_{1}}{{\varphi }_{1}{\Delta l}_{2}-{\varphi }_{2}{\Delta l}_{1}}L\end{array}\right.$$

Where $\Delta {l}_{{{{\rm{t}}}}}$ is target axial strain, ${\varphi }_{{{{\rm{t}}}}}$ is target twisting density. ${\Delta l}_{1}$ and ${\Delta l}_{2}$ are axial strain at *θ*_*1*_ and *θ*_*2*_, respectively, ${\varphi }_{1}$ and ${\varphi }_{2}$ are twisting density at *θ*_*1*_ and *θ*_*2*_, respectively. This method extends naturally to three or more segments, effectively allowing any combination of twist and axial strain within the design space (Supplementary Note 5). Figure 2g illustrates this multi-segment programming strategy. Four representative actuation modes are highlighted on the design space plot: (i) pure contraction (significant Δ*l* shortening, minimal twist), (ii) macroscopic invariance (negligible Δ*l* and *φ*), (iii) pure twist (rotation without length change), and (iv) extension (positive Δ*l* with little twist). For each of these target points, we designed a filament with two segments (with appropriate *θ*_*1*_, *θ*_*2*_ and segment lengths) to achieve that behavior. The blue and white markers in the figure indicate the programmed orientations of the two segments for each case, demonstrating how combining two distinct programmed helix angles (*θ*_*1*_ and *θ*_*2*_ for the first and second segments, respectively) yields the overall targeted deformation. Figure 2h shows the actual printed filaments corresponding to these four cases, confirming that each filament, when heated, exhibits the intended actuation mode (contracting, remaining neutral, twisting, or elongating as designed). It is worth noting that the twist and length deformation discussed here refer to the macroscopic end-to-end net rotation and axial strain. For instance, in mode (ii), adjacent segments with opposing programmed angles twist in opposite directions upon heating, but they cancel out globally, resulting in a macroscopically invariant shape. We further explored the flexibility of this approach by achieving a single target deformation through multiple different filament designs. In one example, two distinct two-segment configurations (points v and vi in Fig. 2h) were programmed to produce the same overall actuation, both designs resulted in virtually identical deformation behavior upon heating. Moreover, we found that the same target actuation could be realized by increasing the number of segments: points vii and viii correspond to filaments divided into 4 segments and 6 segments, respectively, each programmed in such a way that the final heated shape matched the same target Δ*l*_*t*_ and *φ*_*t*_. In all cases, the printed filaments successfully achieved their prescribed combinations of axial and torsional deformation (Fig. 2i). These results highlight the versatility of the rotational printing method—by appropriately segmenting the filament and controlling *θ* along its length, one can program a wide range of complex 3D actuation responses that would be unattainable with a uniform filament design alone.

### Programming helical angles for versatile shape transformations

**Fig. 3:**
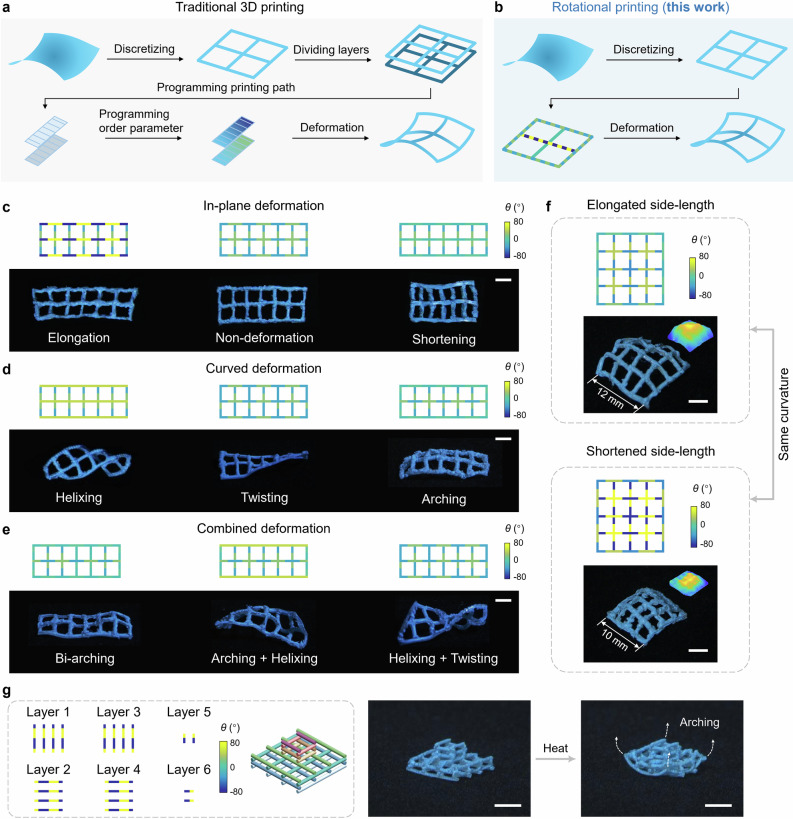
Helical angle programming of a LCE mesh structure yields multiple shape-morphing modes. **a** Traditional multi-step LCE programming with a one-dimensional director field. **b** Single-parameter (*θ*) programming in a 2D LCE filament mesh, enabling diverse in-plane deformations, illustrating how spatial variations in *θ* enable diverse in-plane deformations. **c** Actuated shape changes remain in-plane, with the mesh reconfiguring within the original plane without out-of-plane deflection. (scale bar = 3 mm) (**d**) The mesh undergoes out-of-plane bending, morphing the initially flat structure into a three-dimensional curved surface. **e** Simultaneous in-plane reconfiguration and out-of-plane bending yield complex three-dimensional deformations characterized by coupled bending and twisting (**f**) Decoupling of deformation parameters: two curved strips with the same curvature (*K* = 0.35) but different length changes (one contracts, the other elongates), resulting in a ~ 1.4× difference in length. (Scale bar=5 mm) (**g**) A 6-layer pyramid fabricated by out-of-plane stacking. Upon heating, the structural stiffness gradient firmly anchors the thick central core, forcing the thermal actuation to concentrate at the compliant perimeter and amplifying its upward curl. (Scale bar: 5 mm).

Building on the helical-angle programming strategy for one-dimensional LCE filaments, we extend it to two-dimensional LCE mesh structures, enabling 2D to 3D shape morphing. Traditional LCE 3D-printing approaches require a complex, multi-step programming process because of their one-dimensional director field (Fig. 3a)^50,51^. In contrast, our approach enables a wide range of deformations by programming only a single parameter—the helix angle *θ* (Fig. 3b). For experimental demonstration, we use a standardized platform consisting of a 3 × 7 array of orthogonal LCE filaments arranged in a rectangular grid. All filaments have a uniform diameter and are evenly spaced, ensuring consistent conditions for observing deformation behavior. Figure 3c illustrates a lateral elongation mode, in which the transverse filaments are programmed with large helical angles while the longitudinal filaments are programmed with alternating positive and negative critical-angle segments. This design leads to a pronounced expansion in the lateral (width) direction with negligible change in the longitudinal (length) direction. In the macroscopically invariant state, both transverse and longitudinal filaments are patterned with alternating critical angles, resulting in a thermally stable mesh that maintains nearly constant dimensions upon heating. Conversely, lateral contraction is achieved by programming the transverse filaments with small helical angles (inducing contraction in width) and the longitudinal filaments with large helical angles (inducing extension in length). This combination produces a net decrease in width accompanied by an extension in the mesh length. Notably, in all these planar deformation modes the mesh remains flat, with no significant out-of-plane deformation.

In the helical twisting mode (Fig. 3d), all transverse filaments are programmed to the same critical helical angle, while the longitudinal filaments alternate between active (deforming) and stable (non-deforming) segments. This programming causes the entire mesh to twist around its longitudinal axis, with the angular displacement gradually decreasing from the center toward the edges. For a pure torsional deformation, the longitudinal filaments are patterned with alternating active and stable segments, inducing a globally twisted surface, while the transverse filaments remain unaltered to provide structural support and maintain the mesh’s integrity (Supplementary Fig. 15, Supplementary Movie 2). In contrast, the arching mode is achieved by programming filaments in the inner region with large helical angles (causing significant elongation) while the edge filaments remain at the critical angle (dimensionally stable). Consequently, the central portion of the mesh rises out-of-plane, forming a dome-like arch.

We next demonstrate combined deformation modes that integrate these basic transformations to achieve more sophisticated shape morphing (Fig. 3e). For example, in a dual-arch configuration the mesh is divided into two regions with large helical angles of opposite sign (positive vs. negative), separated by a central stable section; this design produces two arches of different sizes side by side, demonstrating localized deformation control. In an arch-helix combination, coordinated programming of the central and edge regions produces simultaneous arching and twisting: the inner region arches upward while the edges undergo helical rotation. Notably, the direction of arching in this mode depends on the imposed rotation angle, indicating a complex mechanical coupling between arching and twisting. Finally, in a helix–torsion mode, the left portion of the mesh is programmed for torsional twisting while the right portion is programmed for a helical rotation. The deformation transitions smoothly from one mode to the other across the span of the structure, demonstrating a continuous, region-specific shape change within a single integrated design (Supplementary Movie 3).

Beyond enabling these diverse modes via programming *θ*, our method also allows the decoupling of different deformation parameters (e.g., length vs. curvature) in the printed objects. This means one aspect of deformation can be tuned independently of another, without mutual interference. As a demonstration (Fig. 3f), we printed two curved surfaces with the same target curvature (*K₁* = *K*_*2*_ = 0.35) but opposite programmed length changes—one designed to contract (side length 10 mm) and the other to elongate (side length 12 mm). Upon actuation, both surfaces exhibit the same curvature, whereas the elongated surface’s length is ~1.4 times that of the contracted surface, confirming independent control of curvature and length. To demonstrate scalability beyond 1D/2D constructs, we printed free-standing multi-layer structures by out-of-plane stacking (e.g., a 6-layer pyramid, Fig. 3g) This 3D architecture leverages a pronounced structural stiffness gradient to guide localized morphing. The thick multi-layer core strongly resists bending, acting as a geometric anchor that funnels the *θ*-programmed actuation outward, thereby significantly amplifying the upward curling of the highly compliant single-layer perimeter. We also demonstrate that individual rotationally printed filaments can bridge millimeter-scale gaps, with sagging that increases at higher rotational angles (Supplementary Fig. 16, Supplementary Movie 4).

### Programmable sequential deformation for self-partitioning grippers

**Fig. 4:**
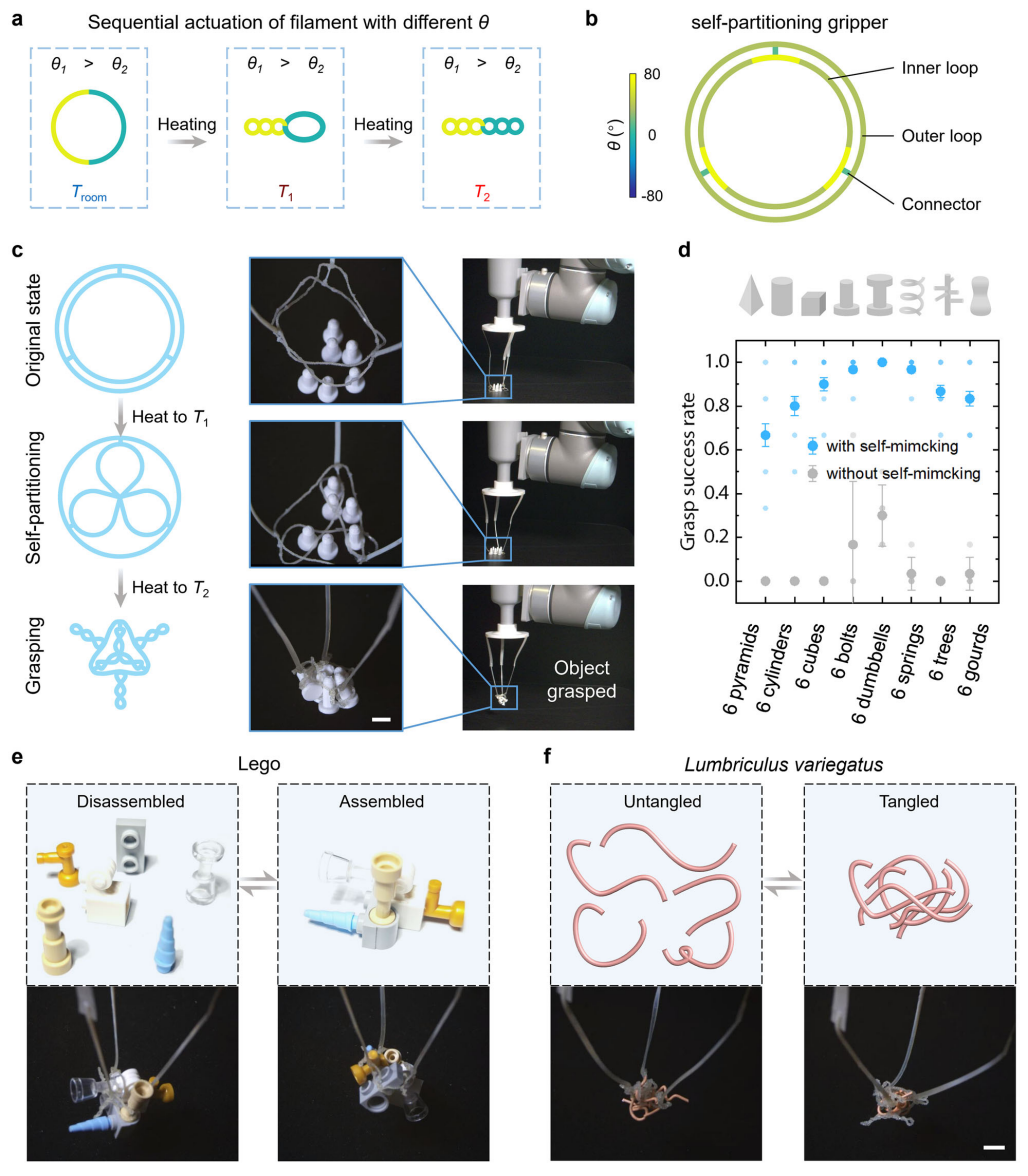
Programmable sequential deformation for a self- partitioning gripper. **a** A printed ring patterned with two distinct helix angles *θ₁* < *θ₂*. Upon heating to an intermediate temperature (*T*_*1*_), only the filament segment with the larger angle (*θ₂*) exceeds the activation threshold and supercoils. **b** Gripper design featuring two concentric loops of slightly different diameters, connected by three struts. **c** Initial heating contracts the inner loop’s high-helix segments, forming three small inner loops, further heating coils the outer loop around the objects, achieving a secure multi-object grasp. **d** Comparison of grasp success rates for six objects, with and without partitioning. **e** LEGO bricks, either assembled or scattered, are adaptively grasped by selective partitioning. **f** Multiple *Lumbriculus variegatus* models are captured in both tangled and untangled states. (scale bar = 5 mm). Bars/points show mean ± SD from *n* = 5 independent trials.

Nematic alignment in liquid crystal elastomers (LCEs) provides the ability to control the sequence of actuation by tuning the onset deformation of supercoil deformation (Supplementary Fig. 17, and Supplementary Fig. 18). As shown in Fig. 4a, a LCE filament is printed into a complete ring whose circumference is partitioned into two equal arcs: one with a smaller helix angle *θ₁* and the other with a larger helix angle *θ₂* (*θ₁* < *θ₂*). When the ring is heated to an intermediate temperature *T₁*, only the *θ₂* segment exceeds its activation threshold and coils into a super-helical configuration, whereas the *θ₁* segment remains essentially undeformed. Upon further heating to a higher temperature *T₂* (*T₂* > *T₁*), both segments actuate and the entire ring coils as a unified structure. This occurs because the higher twist density effectively pre-loads the segment, bringing it closer to the energy barrier required for plectoneme genesis (supercoiling). Consequently, the segment with the smaller helix angle *θ₁*, which presents a higher energy barrier—remains in its initial state until the temperature reaches a higher threshold, *T₂*, where thermal stress is sufficient to trigger deformation. This experiment unequivocally demonstrates that the temporal sequence of actuation is encoded within the filament’s geometry. Such differential thermal sensitivity enables stepwise actuation, governed by the elastic energy stored in the helical director field (Supplementary Fig. 19, and Supplementary Fig. 20). Specifically, the pre-encoded helical director field in high *θ* segments acts as a geometric pre-load, storing higher elastic energy that lowers the macroscopic energy barrier for mechanical instability^52^. To demonstrate this capability, we designed a self-partitioning gripper that undergoes programmed deformations to grasp either single or multiple objects. Grasping multiple objects simultaneously is challenging for traditional grippers, which typically enclose objects externally and rely on friction between the objects^53^; such methods are limited in adaptively adjust to different numbers or shapes of objects^54–57^. Our self- partitioning gripper overcomes this limitation by actively partitioning its structure during the grasping process. As shown in Fig. 4b, the gripper consists of two concentric loops of slightly different diameters, joined by three evenly spaced connectors around the circumference. Both the inner and outer loops are fabricated with a baseline helical alignment of 30°, but at each connector the inner loop’s local helix angle is increased to 70° to facilitate sequential actuation (Supplementary Fig. 21a). We fabricated the gripper using an LCE with a low nematic–isotropic phase transition temperature ( ~ 18 °C) to minimize heat-induced damage to target objects. The completed device was connected to a custom-designed heat gun through a soft connector and adapter and mounted on a 5-axis robotic arm (Supplementary Fig. 21b, c). In the present proof-of-concept setup, heating was used as an open-loop trigger for the programmed LCE deformation rather than as a closed-loop thermal control system. To grasp multiple objects, we first heat the gripper just above its transition temperature so that only the inner loop actuates. The inner loop’s three high-helix segments contract, forming three small inner loops that effectively partition the outer loop into three open sectors. The target objects are placed into these separate compartments. Upon further heating, the outer loop actuates and coils around each object, allowing the gripper to securely capture multiple targets in parallel (Fig. 4c, and Supplementary Fig. 22, Supplementary Movie 5). Figure 4d quantifies the benefit of this strategy. In tests where the gripper attempted to lift six objects of various shapes at once, the sequential self-partitioning approach produced a much higher success rate than a non-partitioning grasping method. Here, the success rate for a single trial was defined as the fraction of the six objects that were successfully lifted and retained during the lifting step.

Enabled by its adaptive partitioning, the gripper can handle multiple objects whether they are scattered or initially grouped as one entity. For example, Fig. 4e shows that a collection of LEGO^®^ bricks can be picked up in two configurations: assembled into a single block, or separated into individual pieces. In the case of scattered pieces, the gripper first partitions itself (via the inner loop actuation) before grasping, whereas no partitioning is needed to grasp the assembled block (Supplementary Movie 6). In nature, certain organisms tangle and untangle their bodies in response to environmental stimuli. For instance, the aquatic worm *Lumbriculus variegatus* will entangle into a single clump when humidity is high and then disperse into individuals when humidity drops. As shown in Fig. 4f, our gripper can likewise grasp multiple *L. variegatus* worm models in both their tangled (collective) and untangled (separate) states, highlighting a bioinspired parallel in adaptive grasping behavior (Supplementary Movie 7).

### Programmable volatile and non-volatile configuration memory

**Fig. 5:**
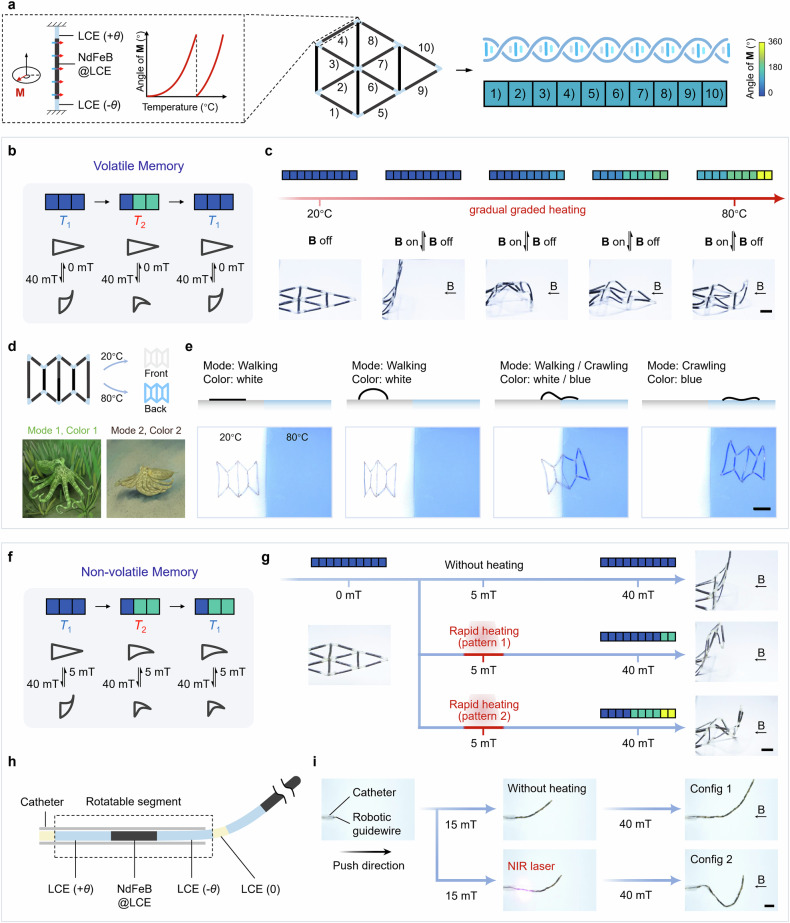
Magnetic-LCE structures with programmable volatile and non-volatile memory. **a** Each LCE leaf is composed of 10 discrete LCE segments, each containing two types of LCE sub-structures with opposite twist and a magnetic composite substructure, resulting in rotation of magnetization under thermal actuation. **b** Increasing the external temperature induces a coordinated global rotation in the LCE leaf (collective twisting of all segments), which disappears upon cooling—demonstrating volatile memory. **c** This temperature-driven reconfiguration leads to varied deformation profiles when the leaf is subjected to a magnetic field. (scale bar=5 mm) (**d**) Four segment LCE crawler demonstrating color and gait switching enabled by volatile reconfiguration. **e** Robot segments flip from white to blue as they traverse an 80 °C zone, transforming a walking robot into a crawling one by simultaneously reconfiguring its magnetization. (scale bar=5 mm) (**f**) Under a constant low magnetic field (5 mT), a localized spike in temperature triggers a locked reorientation in the leaf (twisting in specific segments), which is retained after the leaf cools, indicating non-volatile memory of the magnetic response. **g** Under a low magnetic field (5 mT), rapidly heating certain segments induces local deformations that rotate their magnetic domains by ~180°. Under a stronger magnetic field (40 mT), these reprogrammed states cause the leaf to deform into different shapes (scale bar=5 mm). **h** Enabled by thermal reprogramming, a robotic guidewire is designed with reprogrammable magnetic actuation when it grows from a catheter. **i** Using a near-infrared (NIR) light-induced reprogramming, the robotic guidewire’s magnetic actuation is reprogrammed from an L shape to a U shape (scale bar=5 mm).

To realize versatile and reconfigurable soft robotics, materials are required to exhibit tunable memory capabilities, allowing for both reversible shape shifting and reprogramming of their actuation behaviors. Here, we introduce magnetic composites not as a primary actuator, but to enable reprogrammable volatile and non-volatile memory on top of the printed helical director fields. The composite structure comprises 10 discrete LCE segments arranged in series, each functioning as a tri-section composite unit. The two terminal ends of each segment are LCE components fabricated via multi-step rotational printing (Supplementary Fig. 23), imparted with specific helix angles *θ* so that they undergo predetermined multi-angled twisting deformations upon thermal activation (Supplementary Fig. 24a). The central portion of each segment is a magnetized NdFeB–LCE composite. When heat is applied, the end LCE components twist, causing the central magnetic section to rotate and thereby reorient its magnetic domains (Fig. 5a, Supplementary Fig. 25).

By tailoring the helix angles of the terminal LCE segments based on the twist–deformation profile from the printing process, the rotation angle of each central magnetic segment can be precisely controlled, effectively programming the leaf’s magnetic response behavior. The LCE composite also exhibits dynamic, temperature-dependent configuration changes. An increase in external temperature induces a coordinated global rotation of all segments, which is reversed when the temperature decreases. This reversible behavior indicates a volatile memory effect, as the thermally induced configuration change “vanishes” upon cooling (Fig. 5b). As shown in Fig. 5c and Supplementary Fig. 26a and Supplementary Movie 8, gradually raising the ambient temperature from 20°C to 100°C causes the LCE segments to progressively reorient, resulting in a spectrum of different deformed shapes when the structure is subsequently exposed to a magnetic field. This volatile reconfiguration capability facilitates adaptive robotic functions. Drawing on the mimic octopus (*Thaumoctopus mimicus*), which simultaneously alters body shape and color^58,59^, we built a four-segment crawler with different colors on each side (Fig. 5d, Supplementary Fig. 24b). At 20 °C the robot walks with its white surface uppermost; upon entering an 80 °C zone the heated segment twists, flipping to its blue side and altering both magnetization and gait. Successive flips progressively convert a white, walking robot into a blue, crawling one, demonstrating multi-modal adaptation encoded entirely in the material (Fig. 5e, Supplementary Fig. 26b, Supplementary Movie 9).

Conversely, a sudden localized thermal stimulus can induce a non-volatile change in the LCE leaf’s configuration that persists even after the stimulus is removed. In other words, a rapid, concentrated heating of a specific region produces a locked reorientation in those LCE segments, and this altered state remains memorized in the leaf’s magnetic response after cooling, exemplifying a non-volatile memory effect (Fig. 5f). The sequence illustrated in Fig. 5g and Supplementary Movie 10 demonstrates these principles in detail. Initially, without any induced reprogramming, the structure remains consistent and stable as the strength of an applied magnetic field is increased. Next, under a moderate magnetic field of 5 mT, segments 9 and 10 are rapidly heated with a heat gun, inducing local deformations that produce an ~180° rotation in their magnetic domain orientation. When the magnetic field is subsequently increased to 40 mT, the tip of the structure bends downward as a result of the earlier thermal reprogramming. Finally, further localized heating of the front and middle segments causes the structure to deform into an N-shaped configuration under the magnetic field (Supplementary Fig. 27a). Throughout these adaptive transformations, the LCE structure demonstrates an ability to adjust its torsional configuration and magnetic orientation in response to changing thermal and magnetic conditions. Building on this non-volatile magnetization programming capability, we developed a magnetically steerable soft guidewire as a biomedical robotic application. As shown in Fig. 5h and Supplementary Fig. 24c, the guidewire is assembled by connecting multiple tri-section LCE composite units end-to-end, with small intervening pieces of non-rotating LCE for thermal isolation between units. When the guidewire is gradually extended out of a catheter under a 15 mT magnetic field, it initially bends slightly upward; without any thermal-induced reprogramming it settles into an upward-curving L shape under the field. However, by selectively heating the third and fourth segments with a near-infrared laser, we reorient the magnetization of those segments in opposite directions. Consequently, the guidewire reconfigures into a U shape under the same magnetic conditions (Fig. 5i, Supplementary Fig. 27b, Supplementary Movie 11). This reprogrammable change in shape and trajectory highlights the potential of rotationally printed LCE-based architectures for biomimetic robotics and biomedical applications, where adaptive, reprogrammable actuation is highly desirable.

## Discussion

In summary, this rotational 3D printing strategy significantly advances material-based soft actuation by enabling single-step, single-material programming of multi-modal actuation in LCEs via helical director fields. This approach effectively decouples actuation from geometry: an actuator’s deformation behavior is no longer dictated by its shape or print path but by the encoded helical orientation of its liquid crystal director. As a result, one device can be instructed to contract, bend, or twist solely through its internal alignment, obviating the need for complex multi-material architectures or temperature field control. Embedding such morphological instructions at the material level greatly simplifies system design, effectively shifting control complexity into the actuator itself. Proof-of-concept demonstrations validate these capabilities: an adaptive gripper exploits the programmed deformations to conform to objects, and a volatile/non-volatile actuator displays sequential shape transformations, illustrating the diverse behaviors achievable with this approach. However, control of 3D director fields is currently constrained to simple helical patterns, which limits the range of attainable morphing modes. Moreover, scaling to more complex or larger devices poses challenges in maintaining uniform alignment and actuation consistency. Future efforts should expand the spatial resolution of director control beyond uniform helices, integrate sensing functionalities into the material, and incorporate closed-loop feedback strategies to enable autonomous adaptive behavior in soft robots.

## Methods

### Ink preparation

#### Materials

The main components of LCE ink included 2,2’-[1,2-Ethandiylbis(oxy)]bis(ethanthiol) (EDDET, Sigma-Aldrich, USA), 1,4-bis-[4-(6-acryloyloxyhexyloxy)benzoyloxy]−2-methylbenzene (RM82, Zhende Chemical Technology Co., Ltd.,China), 1,4-Bis-[4-(3-acryloyloxypropyloxy)benzoyloxy]−2-methylbenzene (RM257, Zhende Chemical Technology Co., Ltd.,China), 1,3,5-triallyl-1,3,5-triazine-2,4,6(1H,3H,5H)-trione (TATATO, Sigma-Aldrich,USA),triethylamine (TCI,Japan), antioxidant BHT (Sigma-Aldrich,USA),and photoinitiator 651 (BASF,Germany). NdFeB microparticles with an average size of 38 µm (XND-LW-N-400) were sourced from Guangzhou Xinuode Transmission Components Co., Ltd.

#### LCE ink synthesis

The LCE ink was synthesized via a thiol-acrylate “click” reaction. These components were mixed at a molar ratio of EDDET:RM82:RM257:TATATO = 1:0.6:0.2:0.133. Subsequently, 1 wt% triethylamine, 2 wt% antioxidant BHT, and 1.5 wt% photoinitiator 651 were added. The mixture was thoroughly mixed and melted using a heat gun in a round-bottom flask, then heated in an oil bath at 65 °C for ~3 h to obtain the LCE ink material.

#### Magnetic LCE ink synthesis

For magnetic LCE preparation, NdFeB microparticles were added to the as-synthesized LCE ink followed by manual stirring. The mixture was heated to 90 °C and blended with a planetary mixer (ARV-310, Thinky) at 2000 rpm for 2 min. The well-mixed magLCE ink was transferred into 10 ml syringe barrels and degassed in a vacuum oven overnight. After printing, the ink was magnetized using an impulse magnetizer (M20-2020, HLT Co. LTD) with approximately 2.8 T magnetic field. In this work we use a composite material with 10 vol% NdFeB microparticles (38 μm average diameter) mixed with the LCE ink. The mixture was processed in an ultrasonic disperser for 30 minutes to ensure uniform distribution of magnetic particles.

### Thermal characterization of inks

Differential scanning calorimetry (DSC) was performed to determine the thermal transitions of the LCE ink. Measurements were carried out on a calibrated DSC instrument (DSC Q2000, TA Instruments, USA) under a nitrogen atmosphere at a heating/cooling rate of 10 K min^–1^. Samples were analyzed via a heat-cool-heat cycle between −50° and 150°C with ramp rates of 10 °C/min to clear the thermal history on the first heating ramp and to access both the glass transition temperatures and nematic-to-isotropic transition temperatures of the inks. Samples were held isothermally for 1 min at both high and low temperatures. Data from the second heating ramp were analyzed to determine their *T*_*NI*_ values.

### Rotational printer

#### System setup

The printing system comprises five modules: (i) an air-supply and pressure-control module, (ii) a computer-control module, (iii) a UV-curing module, (iv) a motion module and (v) a rotational print-head module. The air-pressure module combines an air compressor (SY-750X-40 L; Zhejiang Shengyuan Compressor Manufacturing, China) with a high-precision digital dispenser (ML-6000X; MUSASHI Engineering, Japan), delivering 0–700 kPa with 0.1 kPa resolution. System control is provided by an Octopus mainboard, a Raspberry Pi single-board computer and a display, running Klipper firmware. UV curing is achieved with a mercury lamp (Omnicure S2000; Excelitas, USA) at an irradiance of 20 mW cm⁻², which rapidly cross-links the extruded LCE ink to lock in helical alignment. The motion module includes a three-axis platform that employs precision ball-screw stages (travel: X = 300 mm, Y = 300 mm, R = 360°) and a Z stage (Z = 100 mm).

#### Rotational printhead module

The rotational printhead module was mounted on the Z-axis stage. The rotational control module was designed to rotate the printing nozzle without tangling the air supply lines. It incorporated an air slip ring (MK21-1, Shenzhen Moflon Technology Co., Ltd.,China), stepper motor, and synchronous pulley components. The stationary end of the air slip ring was fixed to the Z-axis mounting plate, while the rotating end was connected to the nozzle fixture at the bottom. The upper end was connected to a 42 stepper motor (42BYGH28, Shenzhen Jialibao Electronic Technology Co., Ltd.,China) via synchronous pulleys (HTD-213, Shenzhen Baimao Mechanical Transmission Parts Co., Ltd.,China), allowing real-time control of the nozzle rotation speed. The calibration module included a two-dimensional micro-motion platform on the nozzle fixture (Shenzhen Dahe Industrial Equipment Co., Ltd.,China) for preliminary correction of coaxiality errors, and a nozzle coaxiality correction module connected to the Z-plate for further reduction of rotational printing coaxiality errors.

### Rotational printing of LCEs

To print the designed filaments or meshes, we created models using SolidWorks software, exported them as STL files, and used the specialized slicing software Ultimaker Cura for slicing to generate preliminary G-code files. The G-code file is a plain text file containing instructions for the 3D printer to implement motion control and peripheral control. To achieve control of the newly added rotating platform and pneumatic extrusion, we re-edited the G-code files using MATLAB script programs to add rotation commands and pneumatic extrusion commands. After generating G-code files using slicing software, the MATLAB script program reads the files, automatically analyzes where to insert rotation control commands and pneumatic control commands, automatically inserts R commands and SET_PIN command codes, and saves the re-edited G-code files. The prepared LCE ink was transferred to a 10 ml dispensing syringe, and printing was conducted after leveling the platform and adjusting coaxial deviation. During the printing process, a pressure of 300 kPa was used, and ~20 mW cm^−2^ of ultraviolet light was applied. After printing, ultraviolet irradiation continued for 30 min to ensure complete crosslinking and curing.

Free-standing multilayer structures (Fig. 3g) and gap-spanning struts (Supplementary Fig. 15) were fabricated by extending the rotational-printing workflow to layer-by-layer build-up. Sliced toolpaths were post-processed to embed nozzle-rotation commands, enabling spatially prescribed helix angles (*θ*) across different strut classes and/or layers within a single print. During deposition, each filament was stabilized via in situ UV curing (irradiance ≈ 20 mW cm⁻²) to rapidly crosslink the ink and maintain geometric fidelity during stacking. For gap spanning, struts were printed between two anchor points across millimeter-scale openings and the resulting sagging was quantified as a function of *θ* (Supplementary Fig. 16). When a long-span strut required a discrete θ transition, a small removable sacrificial support was optionally printed at the switching location on the underlying layer and removed after curing; this auxiliary step does not affect the programmed actuation mechanism.

### Characterization

#### Mechanical tests

The printed single filaments were directly used for mechanical tests. Tensile properties were measured on a fiber testing machine with a constant crosshead speed of 10 mm s^−1^ (YG(B)008E, DARONG, China).

#### Characterization of single filament actuation

To test single filament actuation, printed LCE filament with different rotational angle is placed onto a glass slide. During test, one end of the filament is fixed, a mark point is plotted at the other end of the filament. Then, the glass plate is moved up and down by a z-axis stage to contact or not contact the heating plate (JF-976, Dongguan Changanjinfeng Industry Co., Ltd., China). An industrial camera (acA2440-20 gm/gc, Basler, Germany) was used to record the axial and twisting deformation of the filament. We also used an infrared camera to correct the actual temperature of the filament.

#### Polarized Raman microspectroscopy

Transverse filament cross-sections were analyzed using a polarized Raman microscope LabRAM HR Evolution. Radial line scans recorded the polarization-dependent intensity of the mesogenic C = C stretching band at ~1600 cm^−1^.

### Actuation of LCE actuator

For heating the printed samples, a heating plate (JF-976, Dongguan Changanjinfeng Industry Co., Ltd., China) was used to control the sample temperature, and an infrared camera (ETS320, FLIR, USA) was employed to record the temperature distribution during the heating process.

To evaluate the actuation performance and calculate the work density, LCE filaments with a programmed helix angle of *θ* were subjected to isotonic actuation tests. A filament sample with an initial length *L*_*0*_ and diameter *D* was vertically suspended, with the top end fixed and the bottom end tethered to a load of mass *m*. The sample was then heated from ambient temperature (20 ◦C) to the actuation temperature (100 ◦C) using NIR lights. The vertical displacement Δ*h* of the load, resulting from the combined phase transition and supercoiling of the filament, was recorded using a high-resolution camera.

The mechanical work output (*W*) performed by the filament was calculated as the product of the gravitational force acting on the load and the displacement:

3 $$W=m \cdot g\cdot \Delta h$$

where *g* is the gravitational acceleration (9.8 m/s^2^). The volumetric work density (*U*) was then derived by normalizing the work output by the initial volume of the active LCE filament *V*:

4 $$V=\pi {\left(\frac{D}{2}\right)}^{2}\cdot {L}_{0}$$

5 $$U=\frac{W}{V}$$

This calculation assumes the deformation is uniform along the active length of the filament during the supercoiling process.

#### Self- partitioning gripper

The gripper was printed using LCE with low transition temperature. For heating the self- partitioning gripper, it is integrated with a custom-designed heat gun via a soft connector and adapter, enabling real-time heating of the gripper. Then the gripper is mounted on a 5-axis robotic arm for grasping of objects. While fully pre-programmed movements were utilized in Movie 5, the robotic arm was temporarily operated in a gravity-compensated kinesthetic teaching mode in Movies 6 and 7 to rapidly validate conformal adaptation. For the grasping experiments in Fig. 4d, six objects of different shapes were placed beneath the gripper in each trial. A successful grasp for an individual object was defined as complete lifting of the object from the substrate and retention during the lifting step. The success rate for one trial was calculated as *N*_*success*_/6, where *N*_*success*_ is the number of objects that satisfied this criterion. After each trial, the objects and the gripper were reset to the initial configuration, and the experiment was repeated independently five times for each condition (*n* = 5).

#### *Magnetic-LCE* composite actuator

By placing the heating stage in a three-dimensional Helmholtz coil (Hunan Paisheng Technology Co., Ltd., China) and programming the magnitude and direction of the magnetic field through Python scripts, both the magnetic field and temperature field within the coil could be precisely controlled simultaneously, thereby actuating the NdFeB–LCE samples. For the leaf actuator and walking robot, it was selectively heated by the heating plate. For the robotic guidewire, it was heated by NIR laser.

### Finite element simulation of magnetic-LCE composite actuator

Deformation of NdFeB-filled composites in uniform fields was simulated in ABAQUS using a user subroutine developed by Zhao et al^60^. The magnetic torque density **τ** = **M×B** is incorporated through a Cauchy stress term **σ**^magnetic^ = **-B**⨂**FM** where **F** is the deformation gradient and ⨂ denotes the dyadic product. Young’s modulus and magnetization as functions of NdFeB volume fraction were calculated following Wang et al^61^.

## Supplementary information

Supplementary Information

Description of Additional Supplementary Files

Supplementary Movie 1

Supplementary Movie 2

Supplementary Movie 3

Supplementary Movie 4

Supplementary Movie 5

Supplementary Movie 6

Supplementary Movie 7

Supplementary Movie 8

Supplementary Movie 9

Supplementary Movie 10

Supplementary Movie 11

Transparent Peer Review file

## Source data

Source Data

## Data Availability

Source Data are provided with this paper. The data supporting the findings of this study are available within the paper, its Supplementary Information, and the Source Data file. All data are available from the corresponding authors upon request. Source data are provided with this paper.

## Code availability

The custom MATLAB scripts used for G-code toolpath post-processing, together with a README describing software dependencies, workflow instructions, and printer/hardware setup, as well as the CAD design files (.STEP format) necessary to replicate the rotational 3D printing setup, are open-source and available in the public Zenodo repository under the accession (10.5281/zenodo.19396817).
