# Supplementary material for: Single-parameter programmed thermomechanical actuation via 3D-printed helical director fields in liquid crystal elastomers
Source: Nat Commun. 2026 May 15;17:6454. doi: 10.1038/s41467-026-73204-y (PMC13376412; doi:10.1038/s41467-026-73204-y)
Supplement: Supplementary file 1 — Supplementary Information [file 41467_2026_73204_MOESM1_ESM.pdf]

Supplementary Information

Single-parameter programmed thermomechanical actuation via 3D-printed helical director fields in liquid crystal elastomers

Yuxuan Sun[1], Boxi Sun[1], Zhengqing Zhu[1], Jiyang Wu[1], Hao Jing[1], Xingxiang Li[1], Dongxiao Li[1], Ziyi Zhang[2], Dongchang Zheng[2], Guorui Wang[2], Weihua Li[3], Yu Xiao[1], Tingrui Pan[4], Yong Chen[5,6*], Shiwu Zhang[1*], Mujun Li[1*]

1. Institute of Humanoid Robots, Department of Precision Machinery and Precision Instrumentation, University of Science and Technology of China, Hefei 230026, China.

2. Department of Modern Mechanics, University of Science and Technology of China, Hefei 230026, China.

3. School of Mechanical, Materials, Mechatronic and Biomedical Engineering, University of Wollongong, Wollongong, NSW 2522, Australia.

4. Suzhou Institute for Advanced Research, University of Science and Technology of China, Suzhou 215123, China

5. Epstein Department of Industrial and Systems Engineering, Viterbi School of Engineering, University of Southern California, Los Angeles, CA 90089, USA

6. Department of Mechanical and Aerospace Engineering, The Hong Kong University of Science and Technology, Hong Kong 999077, China

*Corresponding authors.

E-mail: yongchen@usc.edu (Y.C); swzhang@ustc.edu.cn (S.Z); lmn@ustc.edu.cn (M.L).

These authors contributed equally: Yuxuan Sun, Boxi Sun, Zhengqing Zhu.

Supplementary Notes

Supplementary Note 1. Theoretical Analysis of Oligomer Length

To estimate the length of the synthesized LCE oligomers, a theoretical analysis based on the Carothers equation was performed. The synthesis involves a base-catalyzed thiol-Michael addition between the dithiol (EDDET) and diacrylate mesogens (RM82 and RM257). The allyl-containing crosslinker (TATATO) is inert under these conditions and does not participate in the initial oligomerization.

The molar ratio of functional groups is calculated as follows:

Thiol groups (N_{SH}): $1.0\ \text{mol (EDDET)} \times 2 = 2.0$

Acrylate groups ($N_{C=C}$): $\big(0.6\ \text{mol (RM82)} + 0.2\ \text{mol } (R\text{M257})\big) \times 2 = 1.6$

The stoichiometric imbalance ratio *r* is:

$$r = \frac{N_{C=C}}{N_{SH}} = 0.8 \tag{S1}$$

Assuming complete conversion of the limiting acrylate groups ($p \rightarrow 1$), the number-average degree of polymerization $\bar{X}_n$ is:

$$\bar{X}_n = \frac{1+r}{1-r} = 9 \tag{S2}$$

This calculation indicates that the ink is composed of thiol-terminated oligomers with an average length of approximately 9 structural units. Consistent with this prediction, THF-GPC (PS standards) shows an oligomeric molecular-weight distribution with PS-equivalent M_n=6.9 kDa, M_w=14.0 kDa, and $Đ$=2.03 (Supplementary Fig. 4). The excess thiol groups serve to end-cap the oligomers, preventing gelation during synthesis and providing reactive sites for the subsequent UV-initiated crosslinking with TATATO.

Supplementary Note 2. Spontaneous Deformation in LCE Fibers

Consider a cylindrical LCE fiber with an initial radius R_0 and length L, which is in the nematic (as-prepared) state and whose director is aligned along the fiber axis Z. In the reference configuration, the cylindrical coordinates are $\mathbf{R} = (R, \Psi, Z)$; in the deformed configuration, the cylindrical coordinates are $\mathbf{r} = (r, \psi, z)$. The deformation gradient $\mathbf{F}$ can be written as:

$$\mathbf{F} = \frac{\partial \mathbf{r}}{\partial \mathbf{R}} = \begin{pmatrix} \frac{\partial r}{\partial R} & \frac{1}{R}\frac{\partial r}{\partial \Psi} & \frac{\partial r}{\partial Z} \\ r\frac{\partial \psi}{\partial R} & \frac{r}{R}\frac{\partial \psi}{\partial \Psi} & r\frac{\partial \psi}{\partial Z} \\ \frac{\partial z}{\partial R} & \frac{1}{R}\frac{\partial z}{\partial \Psi} & \frac{\partial z}{\partial Z} \end{pmatrix} \tag{S3}$$

Correspondingly, the total elastic energy E can be written as:

$$E = \int_V W(\mathbf{F})\mathrm{d}V \tag{S4}$$

where V is the volume in the reference configuration and W is the energy density determined by F. For an incompressible material, using a neo-Hookean model, the energy density is:

$$W(\boldsymbol{F}) = \frac{1}{2}\mu \mathrm{Tr}(\boldsymbol{F} \cdot \boldsymbol{F}^T) + p(\det(\boldsymbol{F}) - 1) \tag{S5}$$

where μ is the shear modulus and p is the Lagrange multiplier describing the spatially varying pressure field in the material. (The second term enforces volume conservation.)

For an LCE cylinder with spontaneous deformation, the condition of local energy minimization can be expressed as $\mathbf{F} = \mathbf{G}(R, \Psi, Z)$. The tensor $\mathbf{G}$ describes the spontaneous}(stress-free) distortion that a liquid-crystal elastomer undergoes when its internal nematic order changes. In practice, $\mathbf{G}$ embodies the preferential contraction by a factor λ_s along the director and the compensating expansion $1/\lambda_s$ in the transverse directions. However, the spontaneous deformation mode is incompatible, so the cylinder cannot fully attain $\mathbf{G}$; instead, it relaxes to an internally stressed state that minimizes the total elastic energy. If the actual local deformation prior to the spontaneous deformation is $\mathbf{F}$, then the local relaxed elastic deformation is $\mathbf{F} \cdot \mathbf{G}^{-1}$, where the second term represents the effect of the spontaneous deformation and the first term is the applied

actual deformation. Therefore, after the spontaneous deformation, the fiber's elastic energy can be written in the same form:

$$E = \int_V \left[\frac{1}{2}\mu \mathrm{Tr}(\boldsymbol{F} \cdot \boldsymbol{G}^{-1} \cdot \boldsymbol{G}^{-T} \cdot \boldsymbol{F}^T) + p(\det(\boldsymbol{F}) - 1)\right] dV \tag{S6}$$

Minimizing the energy with respect to variations yields the conventional mechanical equilibrium equation for the volume:

$$\nabla \cdot \boldsymbol{\Sigma} = \boldsymbol{0} \tag{S7}$$

along with the free boundary condition:

$$\boldsymbol{\Sigma} \cdot \hat{\boldsymbol{R}} = \boldsymbol{0} \tag{S8}$$

on the free surface. Here $\boldsymbol{\Sigma}$ is the first Piola–Kirchhoff stress tensor, given by:

$$\boldsymbol{\Sigma} = \mu \boldsymbol{F} \cdot \boldsymbol{g} + p\det(\boldsymbol{F})\boldsymbol{F}^{-T} \tag{S9}$$

To solve for the actual deformation field, we first observe that, given axisymmetric, Z -independence, and incompressibility, the deformation takes a simple form as follows:

$$\begin{aligned} r(R,\Psi,Z) &= \frac{R}{\sqrt{\lambda}} \\ \psi(R,\Psi,Z) &= \Psi + \tau\left(Z + f_\psi(R)\right) \\ z(R,\Psi,Z) &= \lambda\left(Z + f_z(R)\right) \\ p(R,\Psi,Z) &= -\frac{f_p(R)}{\lambda} \end{aligned} \tag{S10}$$

where f_ψ, f_z, and f_p are unknown functions of R that modify the original solution for a passive rubber cylinder. The function f_ψ introduces a rotation as a function of radius, allowing a reference radius in the initial configuration to become a curved line in the final configuration. Similarly, f_z encodes a warping of each cross-section as an identically rotated surface, and f_p allows the pressure to vary with radius. Now, the deformation can be decomposed into three independent parts: deformation of the cross-section, torsion, and extension. Incorporating this decomposition into the deformation gradient, we obtain:

$$\boldsymbol{F} = \begin{pmatrix} \frac{1}{\sqrt{\lambda}} & 0 & 0 \\ \frac{Rf'_{\psi}}{\sqrt{\lambda}} & \frac{1}{\sqrt{\lambda}} & \frac{R\tau}{\sqrt{\lambda}} \\ \lambda f'_z & 0 & \lambda \end{pmatrix} = \boldsymbol{\lambda} \cdot \mathbf{T} \cdot \boldsymbol{\Phi} \tag{S11}$$

where $\boldsymbol{\lambda}$ and $\mathbf{T}$ are the pure extension and pure torsion components, respectively. $\boldsymbol{\Phi}$ describe the deformation of the cross-section in the absence of any twist or extension, and this cross-sectional deformation is the only part that depends on R and ψ. Its expression is:

$$\boldsymbol{\Phi} = \begin{pmatrix} 1 & 0 & 0 \\ R\tau\left(f'_{\psi} - f'_z\right) & 1 & 0 \\ f'_z & 0 & 1 \end{pmatrix} \tag{S12}$$

Substituting this form back into the energy expression and applying the principle of minimum energy, one can obtain analytical solutions for f_{ψ}, f_z, and f_p (the detailed derivation is given in [1]).

Going back to the expression of E, we can observe that the spontaneous deformation term can be described by $\mathbf{g} = \mathbf{G}^{-1} \cdot \mathbf{G}^{-T}$, which is in the form of:

$$\mathbf{g} = \begin{pmatrix} g_{RR}(R) & g_{R\Psi}(R) & g_{RZ}(R) \\ g_{R\Psi}(R) & g_{\Psi\Psi}(R) & g_{\Psi Z}(R) \\ g_{RZ}(R) & g_{\Psi Z}(R) & g_{ZZ}(R) \end{pmatrix} \tag{S13}$$

During the nematic-isotropic transition of LCE, it spontaneously contracts by a factor of λ_s along the director, and at the same time stretches by a factor of $1/\lambda_s$ in two perpendicular directions in order to maintain the volume, which corresponds to a spontaneous deformation $\mathbf{G} = \mathrm{diag}\left({\lambda_s}^{-0.5}, {\lambda_s}^{-0.5}, \lambda_s\right)$ in the reference frame aligned with the director. Therefore, $\mathbf{g}$ can be expressed as:

$$\mathbf{g} = \lambda_s^{-0.5} \mathbf{n} \otimes \mathbf{n} + \lambda_s (\mathbf{I} - \mathbf{n} \otimes \mathbf{n}) \tag{S14}$$

As shown in Fig. S5, the helical director field is defined by two constant angles θ and ζ, where θ represents the tilt angle between the director and the fiber axis ($\hat{\mathbf{e}}_Z$), and ζ represents the azimuthal

angle of the director's projection in the cross-section relative to the radial direction $\hat{\mathbf{e}}_r$. For rotationally printed LCE fibers, $\zeta = \pi/2$, thus the director expression is:

$$\mathbf{n} = \sin\theta\,\hat{\mathbf{e}}_\Psi + \cos\theta\,\hat{\mathbf{e}}_Z \tag{S15}$$

From this, one can resolve $\mathbf{g}$ explicitly as:

$$\mathbf{g} = \begin{pmatrix} \lambda_s & 0 & 0 \\ 0 & \dfrac{\sin(\theta)^2}{\lambda_s^2} - \lambda_s(\sin(\theta)^2 - 1) & \dfrac{\cos(\theta)\sin(\theta)}{\lambda_s^2} - \lambda_s\cos(\theta)\sin(\theta) \\ 0 & \dfrac{\cos(\theta)\sin(\theta)}{\lambda_s^2} - \lambda_s\cos(\theta)\sin(\theta) & \dfrac{\cos(\theta)^2}{\lambda_s^2} - \lambda_s(\cos(\theta)^2 - 1) \end{pmatrix} \tag{S16}$$

Substituting the $\mathbf{g}$ field back into the elastic energy calculation and using the decomposition of $\mathbf{F}$, we immediately see the influence of $\mathbf{g}$ fully encapsulated in the symmetric tensor:

$$\tilde{\mathbf{g}} = \mathbf{\Phi}\cdot\mathbf{g}\cdot\mathbf{\Phi}^T = \begin{pmatrix} g_{RR} & 0 & 0 \\ 0 & g_{\Psi\Psi} - \dfrac{g_{R\Psi}^2}{g_{RR}} & g_{\Psi Z} - \dfrac{g_{R\Psi}g_{RZ}}{g_{RR}} \\ 0 & g_{\Psi Z} - \dfrac{g_{R\Psi}g_{RZ}}{g_{RR}} & g_{ZZ} - \dfrac{g_{RZ}^2}{g_{RR}} \end{pmatrix} \tag{S17}$$

This captures the residual part of the cross-section after relaxation, excluding twisting or stretching. With the cross-sectional metric $\tilde{\mathbf{g}}$, the energy of a cylinder of radius R_0 and length L reduces to

$$\frac{E}{\mu\pi L R_0^2} = \frac{a_0}{\lambda} + a_1\lambda^2 + \frac{b}{\lambda}\tau + \frac{c}{\lambda}\tau^2 \tag{S18}$$

with:

$$\begin{aligned} a_0 &= \frac{1}{2}\int_0^{R_0} (\tilde{\mathbf{g}}_{RR} + \tilde{\mathbf{g}}_{\Theta\Theta})R\,dR \\ a_1 &= \frac{1}{2}\int_0^{R_0} \tilde{\mathbf{g}}_{ZZ}R\,dR \\ b &= \frac{1}{2}\int_0^{R_0} \tilde{\mathbf{g}}_{\Theta Z}R^2\,dR \\ c &= \frac{1}{2}\int_0^{R_0} \tilde{\mathbf{g}}_{ZZ}R^3\,dR \end{aligned} \tag{S19}$$

Here a_0 and a_1 encode, respectively, the zero-th radial moments of in-plane and axial spontaneous strains; b captures twist–stretch coupling; and c controls the effective torsional stiffness.

As described in the main text:

$$\theta = \arctan\left(\frac{\omega R}{v}\right) \tag{S20}$$

Substituting it back into the expressions of $\tilde{\mathbf{g}}$, a_0, a_1, b and c,can be calculated as:

$$\begin{aligned}
a_0 &= \frac{{\lambda_s}^3 \log\left({R_0}^2\, \omega^2/v^2 + 1\right) - \log\left({R_0}^2\, \omega^2/v^2 + 1\right) + {R_0}^2\, \omega^2/v^2 + {R_0}^2 {\lambda_s}^3\, \omega^2/v^2}{4{\lambda_s}^2\, \omega^2/v^2} \\
a_1 &= \frac{\log\left({R_0}^2\, \omega^2/v^2 + 1\right) - {\lambda_s}^3 \log\left({R_0}^2\, \omega^2/v^2 + 1\right) + {R_0}^2 {\lambda_s}^3\, \omega^2/v^2}{4{\lambda_s}^2\, \omega^2/v^2} \\
b &= \frac{\left({\lambda_s}^3 - 1\right)\left(\log\left({R_0}^2\, \omega^2/v^2 + 1\right) - {R_0}^2\, \omega^2/v^2\right)}{2{\lambda_s}^2\, \omega^3/v^3} \\
c &= \frac{2\lambda_s^3 \log\left({R_0}^2\, \omega^2/v^2 + 1\right) - 2\log\left({R_0}^2\, \omega^2/v^2 + 1\right) + 2{R_0}^2\, \omega^2/v^2}{8{\lambda_s}^2\, \omega^4/v^4} \\
&\quad - \frac{2{R_0}^2 \lambda_s^3\, \omega^2/v^2 + {R_0}^4 \lambda_s^3\, \omega^4/v^4}{8{\lambda_s}^2\, \omega^4/v^4}
\end{aligned} \tag{S21}$$

Stationarity of the total energy requires:

$$\frac{\partial E}{\partial \tau} = 0, \qquad \frac{\partial E}{\partial \lambda} = 0 \tag{S22}$$

The first condition yields:

$$\tau_m = -\frac{b}{2c} \tag{S23}$$

Substituting $\tau = \tau_m$ into the second stationarity condition produces a cubic equation in λ. Solving gives:

$$\lambda_m = \left(\frac{4a_0 - b^2}{8a_1 c}\right)^{\frac{1}{3}} \tag{S24}$$

which represents the spontaneous axial stretch that minimizes the energy in conjunction with the finite spontaneous twist τ_m. Substituting Eq. S20 into Eqs. S23 and S24, the analytical solutions for $\tau_m$and λ_m could be calculated.

Supplementary Note 3. Conditions for four deformation modes with constant $\boldsymbol{\theta}$

(i) Pure axial contraction without twist ($\lambda_m < 1, \tau_m = 0$).

From Eq. 23 the twist density vanishes when $b = 0$, which requires either $\zeta = 0$ (no helical variation) or $\sin 2\theta = 0$ ($\theta = 0$ or $\theta = \pi/2$). Choosing the practically accessible case $\theta = 0$ (axial director) with $\lambda_s < 1$ yields $a_0 > a_1 > 0$ and hence $\lambda_m < 1$, corresponding to a uniform fiber shortening by a factor $|\lambda_m|$. Physically, the chains contract along the axis while transverse swelling is isotropic, resulting in a straight, untwisted but shorter fiber.

(ii) Axial contraction accompanied by twist ($\lambda_m < 1, \tau_m \neq 0$).

Here $b \neq 0$ demands $\sin 2\theta \neq 0$, i.e., a truly helical director with $0 < \theta < \pi/2$. Axial contraction persists provided the cubic numerator in λ_m is negative: $4a_0c - b^2 < 0$. Using Eq. S24, this inequality is satisfied for tilt angles smaller than a critical value

$$\theta < \theta_c \text{ with} \tan^2 \theta_c = \frac{1 - \lambda_s^3}{\lambda_s^2 - 1} \tag{S25}$$

Experimentally one selects $\theta < \theta_c$ to realise a left- or right-handed twisting fiber that also shortens. The twist originates from geometrical incompatibility between the azimuthal director component and the axial contraction.

(iii) Pure twist without net axial strain ($\lambda_m = 1, \tau_m \neq 0$).

Setting $\lambda_m = 1$ in Eq. S24 gives the condition $4a_0c - b^2 = 0$, which reduces precisely to $\theta = \theta_c$ defined above. Any non-zero ζ then enforces $\tau_m \propto b \neq 0$. Thus, choosing the critical tilt angle

$$\theta = \theta_c = \arctan \sqrt{\frac{1 - \lambda_s^3}{\lambda_s^2 - 1}} \tag{S26}$$

produces a fiber that twists at a constant rate:

$$\tau_m R_0 = \frac{\pi R_0}{4} (\lambda_s^{-2} - \lambda_s) \sin 2\theta_c \tag{S27}$$

while its length remains unchanged ($\Delta L = 0$). The absence of axial strain reflects a delicate balance between director-aligned contraction and transverse swelling.

(iv) Axial extension accompanied by twist ($\lambda_m > 1, \tau_m \neq 0$).

For $\theta > \theta_c$ the cubic numerator in Eq. S21 becomes positive, yielding $\lambda_m > 0$. Because $b \neq 0$ whenever$\zeta \neq 0$ and $\sin 2\theta \neq 0$, the same helical geometry that generated τ_m in cases (ii)–(iii) now produces simultaneous fiber elongation. Physically, as the tilt angle increases beyond θ_c, the swelling component transverse to the director over-compensates the axial contraction, forcing the fiber to lengthen while still relieving mismatch through twist.

Supplementary Note 4. Composite fiber consisting of two serial segments

A cylindrical LCE fiber is formed by bonding segment 1 (reference length L_1) to segment 2 (reference length L_2), so the total reference length is $L = L_1 + L_2$. Segment 1 has helical director tilt θ_1 and, if unconstrained, would develop an axial strain Δl_1 and a twist density φ_1, while segment 2 has tilt θ_2 and free responses Δl_2 and φ_2.

When the two pieces are joined in series and heated together they must share a single axial strain Δl_t and a single total twist density φ_t. Serial compatibility therefore gives the length-weighted relations:

$$\Delta l_t = \frac{L_1}{L}\,\Delta l_1 + \frac{L_2}{L}\,\Delta l_2, \qquad \varphi_t = \frac{L_1}{L}\,\varphi_1 + \frac{L_2}{L}\,\varphi_2 \tag{S28}$$

exactly the result obtained by minimising the total elastic energy with respect to the common stretch and twist.

To determining L_1 and L_2 for a prescribed overall response, let $x = L_1/L$ so that $1 - x = L_2/L$. Eq. S28 yields the linear system:

$$\begin{cases} \Delta l_t = x\,\Delta l_1 + (1-x)\Delta l_2 \\ \varphi_t = x\,\varphi_1 + (1-x)\varphi_2 \end{cases} \tag{S29}$$

Solving for x gives:

$$x = \frac{\Delta l_t\,\varphi_2 - \varphi_t\,\Delta l_2}{\Delta l_1\,\varphi_2 - \varphi_1\,\Delta l_2} \tag{S30}$$

Hence:

$$L_1 = x\,L, \qquad L_2 = (1-x)L \tag{S31}$$

If the denominator $\Delta l_1 \varphi_2 - \varphi_1 \Delta l_2$ vanishes (the two free-deformation vectors are collinear), the target pair $(\Delta l_t, \varphi_t)$ cannot be realised with only two segments; a third segment or different tilt angles is then required.

Supplementary Note 5. Generalisation to $N > 2$ serial segments

Suppose the fiber is assembled from N serial segments with reference lengths L_i and free responses $(\Delta l_i, \varphi_i)$ for $i = 1, \dots, N$. Define the length fractions:

$$f_i = \frac{L_i}{L}, \sum_{i=1}^{N} f_i = 1 \quad (S32)$$

Serial compatibility extends Eq. S28 to:

$$\begin{pmatrix} \Delta l_t \\ \varphi_t \end{pmatrix} = \sum_{i=1}^{N} f_i \begin{pmatrix} \Delta l_i \\ \varphi_i \end{pmatrix}, f_i \geq 0 \quad (S33)$$

Writing this in matrix form:

$$\begin{pmatrix} \Delta l_1 & \Delta l_2 & \cdots & \Delta l_N \\ \varphi_1 & \varphi_2 & \cdots & \varphi_N \end{pmatrix} \begin{pmatrix} f_1 \\ f_2 \\ \vdots \\ f_N \end{pmatrix} = \begin{pmatrix} \Delta l_t \\ \varphi_t \end{pmatrix}, \sum_{i=1}^{N} f_i = 1 \quad (S34)$$

The target deformation **b** is achievable iff the $2 \times N$ matrix **A** has rank 2 and **b** lies in the convex hull spanned by the column vectors $(\Delta l_i, \varphi_i)^{\mathrm{T}}$. In terms of solution space, there are $N - 3$ degrees of freedom in choosing the f_i. A common strategy is to select any two segments whose free-deformation vectors are linearly independent, solve Eq. S29 for their length fractions, and set the remaining segments' fractions to zero. Alternatively one may distribute the residual freedom (e.g. minimise the largest L_i, enforce manufacturing limits, or apply a least-squares criterion) subject to $f_i \geq 0$.

Supplementary Note 6. Supercoil deformation of LCE fiber

Liquid crystal elastomer fibers with helical orientation undergo torsion and develop multiple leaf-like twists when heated and transformed from the nematic phase to the isotropic phase. During this process, as the two ends of the fiber gradually approach each other, some leaf-like twists convert into ring-like twists, continuously changing shape. As the fiber ends further approach, significant axial deformation occurs. This structure is known as a supercoil, similar to DNA supercoiling, and its chiral topology can be represented by the following equation:

$$L_k = T_w + W_r \tag{S35}$$

Where L_k (Linking number) represents the number of rotations of the supercoiled actuator, T_w (Twist) is the twist density of the fiber, and W_r (Writhe) is the coiling produced outside the main axis of the fiber, corresponding to plectoneme supercoiling and solenoid supercoiling respectively. When helically oriented LCE deforms under heat, the fiber's L_k increases. Under high load conditions, L_k is mostly converted to T_w; however, as the load decreases, W_r gradually increases while T_w correspondingly decreases, transitioning the deformation toward plectoneme supercoiling. The elastic potential energy E_s of plectoneme supercoiling is:

$$(E_s)_\lambda = \frac{1}{2} a T_f L_0 \left(\tau_0 + \frac{b\lambda \sin\theta \cos\theta}{T_f^{\frac{1}{2}}} \right)^2 - c T_f L_0 \lambda \sin^4\theta \tag{S36}$$

Where a, b, c are material constants, T_f is the linear density of the fiber, L_0 is the initial length of the fiber, τ_0 is the twist per unit length, λ is the ratio of the contraction length (L minus L_0) to L_0, and θ is the rotation angle. The elastic potential energy E_{tc} of solenoid supercoiling is:

$$E_{tc} = \frac{1}{2}\left(\frac{\varepsilon n T_f^2}{\rho} 10^{-5}\right) \tau \cdot 2\pi L_0 \tau$$

$$= \frac{1}{2} a L_0 T_f^2 \left(\tau_0 + \frac{b\lambda(\pi^2 - 1)^{\frac{1}{2}}}{\pi T_f^{\frac{1}{2}}} \right)^2 \tag{S37}$$

The bending strain energy E_{be} is:

$$E_{\mathrm{be}} = -cT_f \cdot \frac{L_0\lambda(\pi^2 - 1)(\pi + 1)}{\pi^3} \tag{S38}$$

Therefore, the total strain energy $(E_c)_\lambda$ of solenoid supercoiling is:

$$(E_c)_\lambda = E_{\mathrm{tc}} + E_{\mathrm{bc}} = \frac{1}{2}aL_0T_f^2\left\{\tau_0 + \frac{b\lambda}{T_f^{\frac{1}{2}}}\frac{(\pi^2 - 1)^{\frac{1}{2}}}{\pi}\right\}^2 - cT_fL_0\lambda\frac{(\pi^2 - 1)(\pi + 1)}{\pi^3} \tag{S39}$$

This result indicates that under constant deformation, the torsional strain energy in solenoid supercoiling is lower than in plectoneme supercoiling, but the bending strain energy is higher. By setting $(E_s)_\lambda = (E_c)_\lambda$, we can derive the expression for critical twist density τ_{c}, at which level plectoneme supercoiling will be replaced by solenoid supercoiling:

$$\tau_{\mathrm{c}} = \frac{cT_{\mathrm{f}}L_0\lambda\left\{\sin^4\theta - \frac{(\pi^2 - 1)(\pi + 1)}{\pi^3}\right\} - \frac{1}{2}ab^2L_0T_{\mathrm{f}}\lambda^2\left\{\sin^2\theta\cos^2\theta - \frac{(\pi^2 - 1)}{\pi^2}\right\}}{abL_0T_{\mathrm{f}}^{\frac{3}{2}}\lambda\left\{\sin\theta\cos\theta - \frac{(\pi^2 - 1)^{\frac{1}{2}}}{\pi}\right\}} \tag{S40}$$

Helicity is a chiral characteristic, with right-handedness defined as positive and left-handedness defined as negative, which explains the phenomena observed in preliminary experimental investigations. Liquid crystal elastomers printed with different helical orientations possess different chirality, either positive or negative. Two supercoiled liquid crystal elastomers with the same chirality will embrace and twist together upon stimulation; whereas two supercoiled liquid crystal elastomers with opposite chirality will twist individually when unconstrained at their ends, but will each undergo supercoil deformation when their ends are connected.

Supplementary Table 1. Comparison of rotational 3D printing strategies for programming material microstructure and functional mechanisms.

Ref	Material	Programming target	Enabled microstructure	Functional Mechanism	Application
[5]	Fiber-reinforced Composites	Discontinuous Fibers (Micro-scale)	Helically aligned short fibers	Crack steering & deflection	Impact-resistant composites
[6]	Multi-material System	Material Composition (Meso/Voxel-scale)	Spatial helical heterogeneity	Structural anisotropy via modulus mismatch	Artificial muscles; Stiffness-graded lattices.
[7]	Bi-material Systems	Interface / Structure (Meso-scale)	Twisted bi-material interface	Inter-layer mismatch strain	Shape-shifting strips; Coiling actuators.
[8]	Bi-material Systems	Interface / Structure (Meso-scale)	Suture-like helical interface	Geometric mechanical interlocking	Mechanical metamaterials; Protective structures.
This work	Monolithic LCE	Intrinsic Director Field (Molecular-scale)	Helical molecular director field	Rotated intrinsic strain axes	Soft actuator; Adaptive Grippers; Medical Guidewires.

Supplementary Table 2. Comparison of representative LCE programming approaches for controlling director fields and actuation modes.

Approach	Control method	Director field	Couple to geometry / toolpath	Format	Min. feature width	Enabled actuation
Photo - patterning	Polarization pattern, mask	Spatially patterned (mostly planar)	None	thin films	~µm-scale	Rich in-plane deformation; less suited for large or 3D architectures
Mechanical stretching / rubbing	Strain, rubbing direction	Uniform (uniaxial)	Couple with stretching direction	Films / bulk	Sample-scale	Contraction along stretch; limited spatial programming
DIW	Toolpath / degree of alignment	Along filament axis, Piecewise uniform (per printing segment)	High	3D printed parts	$\approx D_f$ (longitudinal) $\geq 3\ D_f$ (transversal)	Segmental programming, requires complex trajectories to program
Rotational printing (this work)	ω/v (θ)	Helical/chiral within filament	Low	2D / 3D printed parts	$\approx D_f$	Twist / contract / extend from same laydown path; program 3D deformation by θ

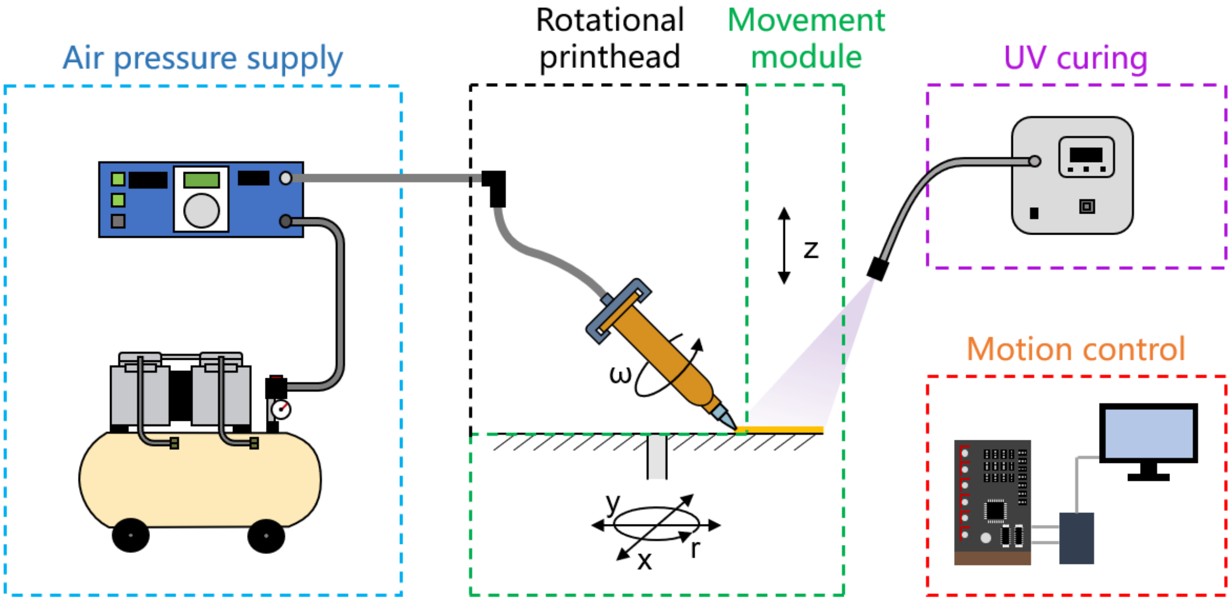

Supplementary Fig. 1. Schematic of the rotational printing system. The movement module enables omnidirectional printing in the x–y plane using a rotational printhead. LCE ink is extruded by an air-pressure supply and is crosslinked in situ by UV light. A control module simultaneously coordinates the movement, rotation, and extrusion processes.

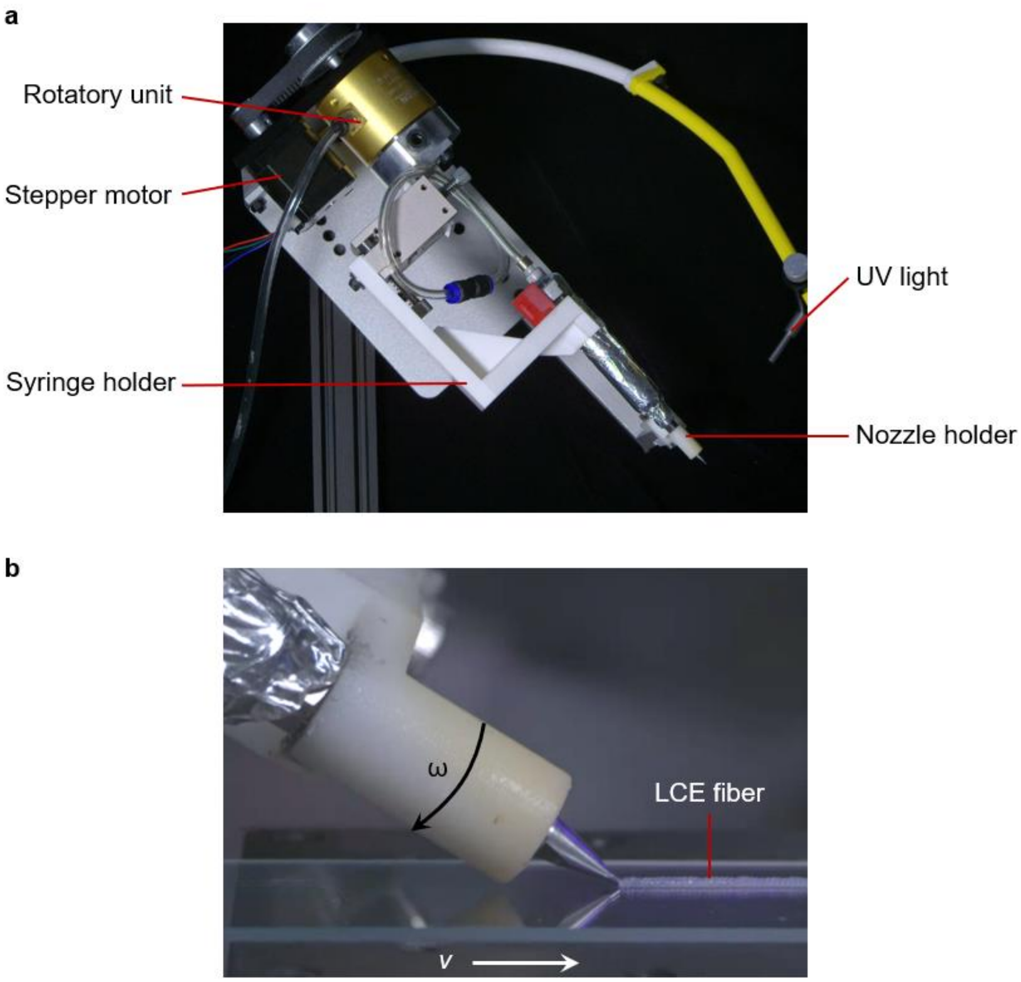

Supplementary Fig. 2. Rotational printhead module and its operation. (a) The module comprises a stepper motor connected to a rotary unit, with a syringe mounted on the rotary unit via a syringe holder. A fixed nozzle holder on the machine frame prevents eccentric motion of the syringe tip, and a UV light source is used for in situ curing of the extruded ink. (b) During printing, the syringe rotates at a tunable speed while the deposited LCE filament is rapidly cured by the UV light.

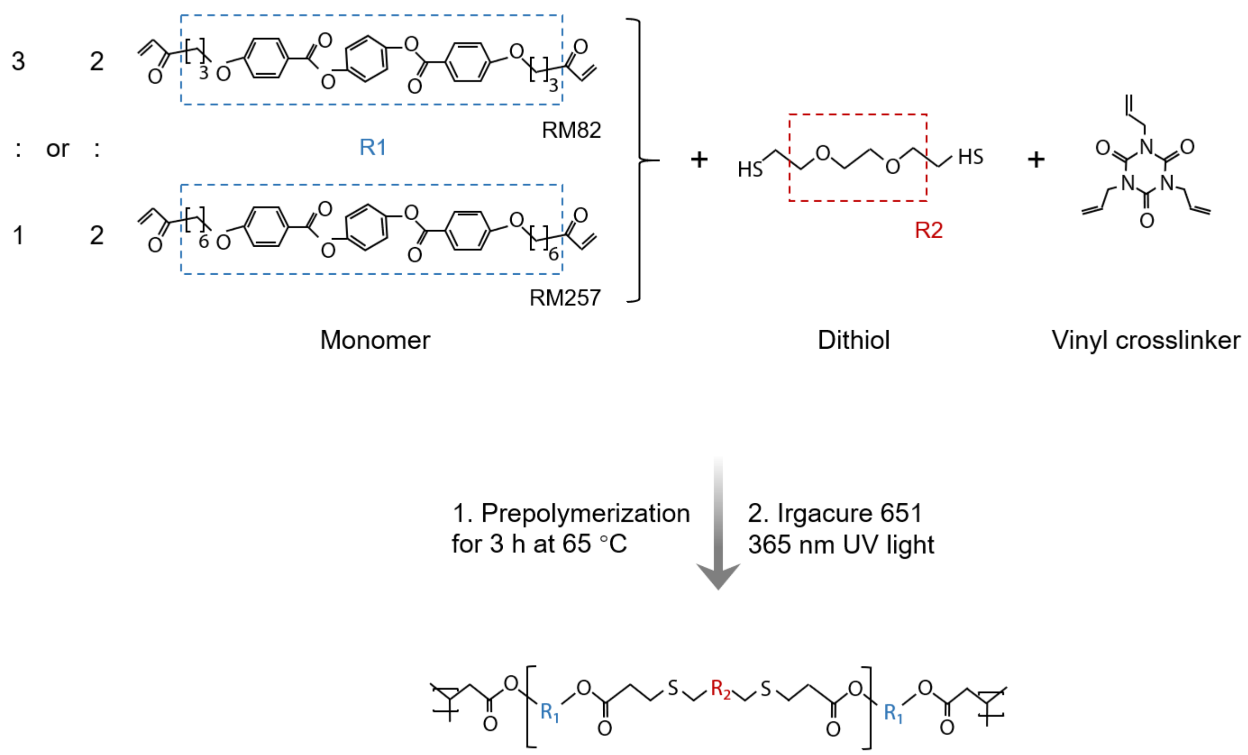

Supplementary Fig. 3. The oligomeric LCE ink is obtained after a prepolymerization step, and subsequent UV crosslinking (20 mW cm^{-2}) during the printing process fully cures the material.

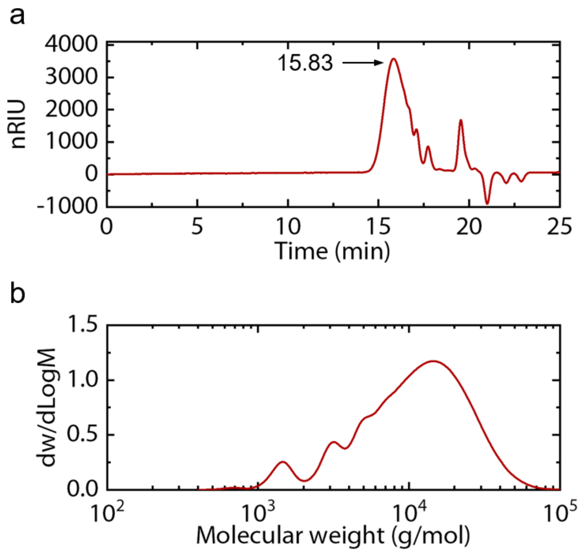

Supplementary Fig. 4. THF-GPC characterization of the LCE oligomer ink. (**a)** Representative refractive-index (RI) chromatogram showing the main elution peak at 15.83 min. (**b)** Corresponding molecular-weight distribution (polystyrene calibration), yielding PS-equivalent M_n=6.9×10^3, M_w=1.4×10^4, and Đ=2.03.

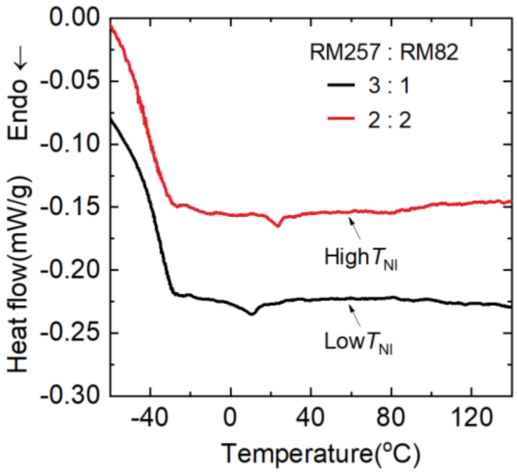

Supplementary Fig. 5. DSC curves of LCE samples with different RM257:RM82 monomer ratios, showing that the phase transition temperature varies with composition.

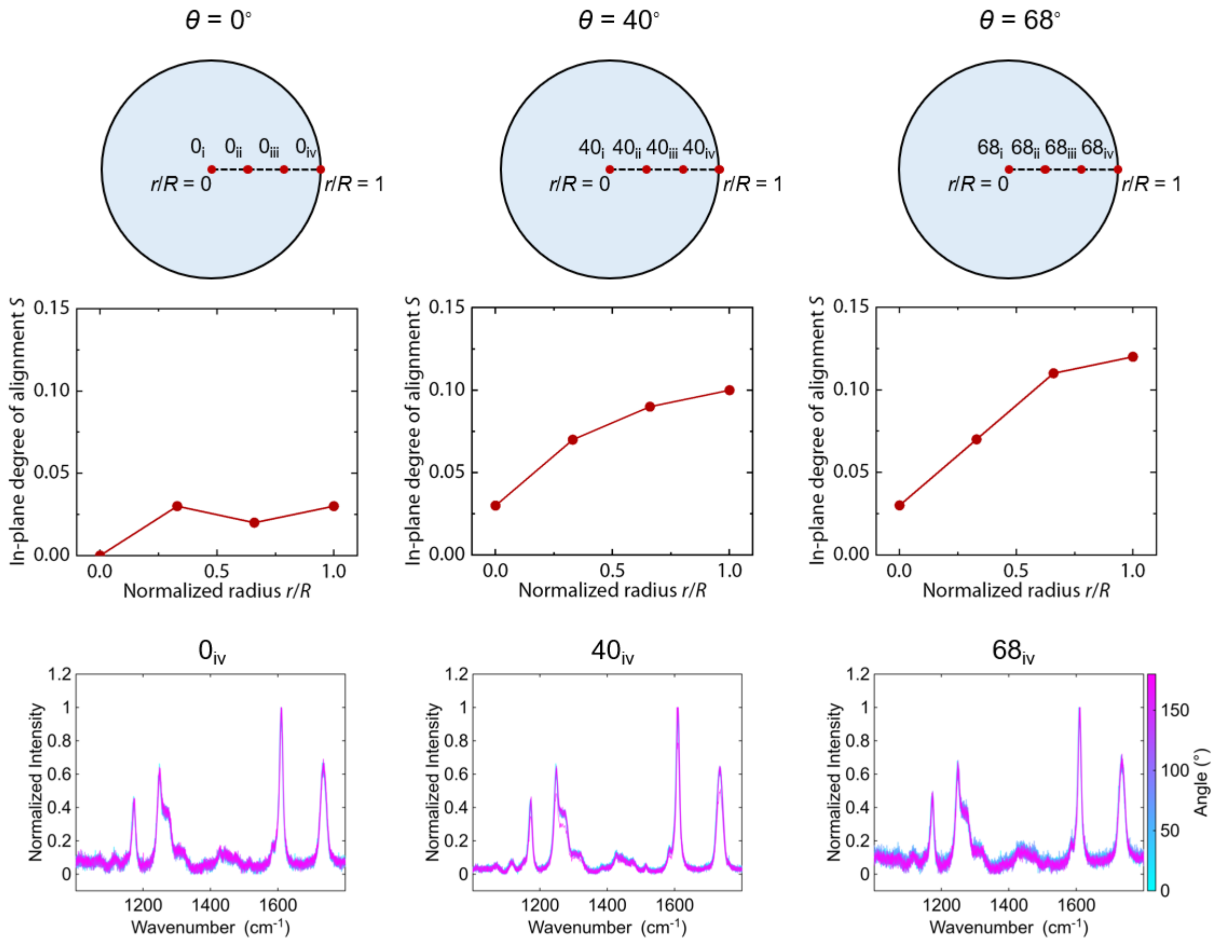

Supplementary Fig. 6. Laser Raman experiment shows higher in-plane rotation of nematic director with larger rotational angle.

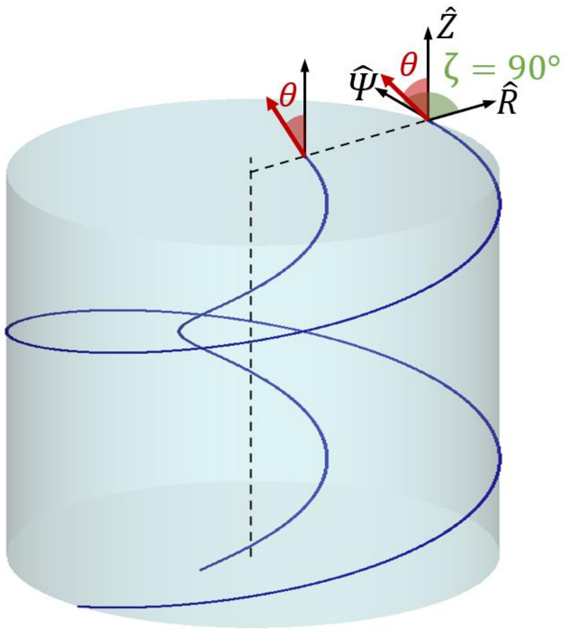

Supplementary Fig. 7. Schematic of a helical director field in a printed LCE filament. The azimuthal angle ζ is fixed at 90°, whereas the director's rotation angle θ varies along the radial direction.

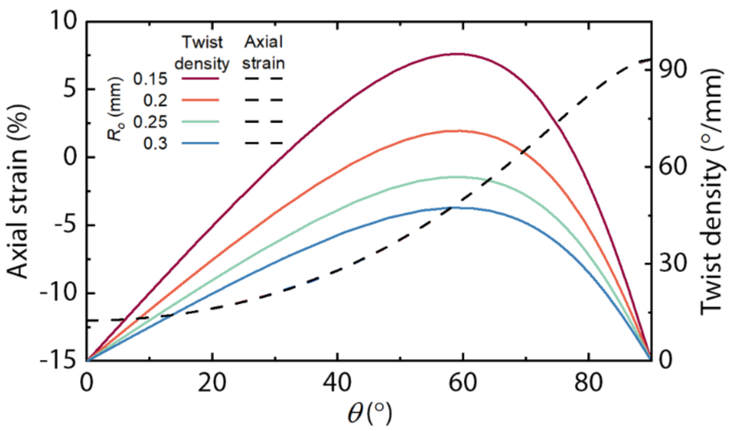

Supplementary Fig. 8. Calculated deformation for filaments of varying radius shows that the axial strain remains constant across different radii, while smaller filament radii result in increased twist deformation.

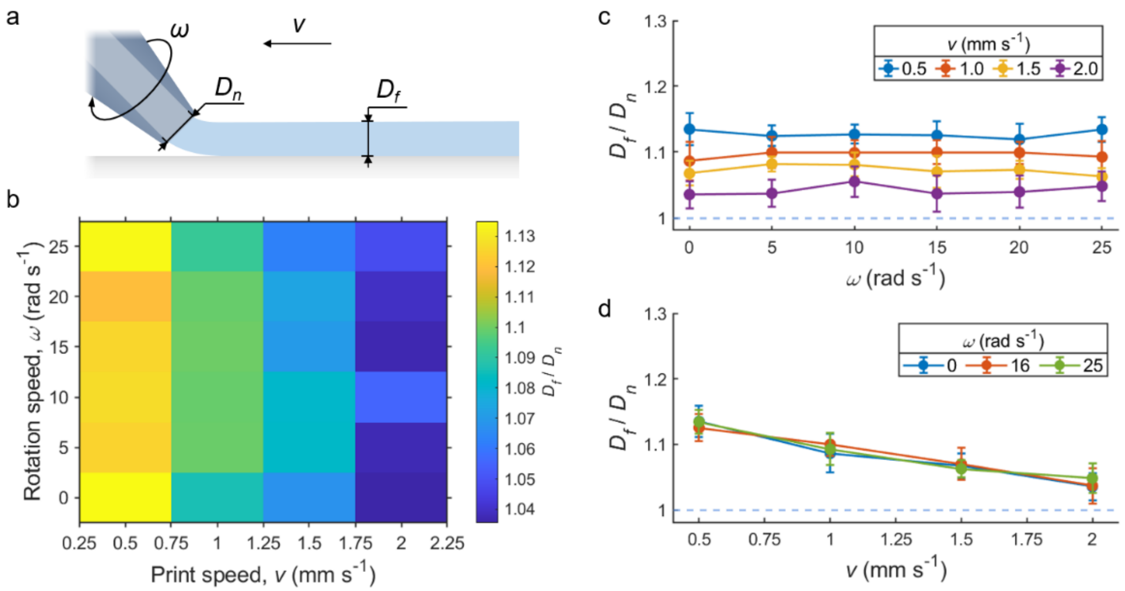

Supplementary Fig. 9. Deposited strand geometry calibration across translation and rotation speeds**.** (a) Definition of nozzle diameter D_n and deposited strand diameter D_f. (b) Map of D_f/D_n across print speed v and rotation speed ω. (c–d) Representative line cuts showing D_f/D_n at different ω and vs. v. Data are presented as mean ± SD, n = 5 independent samples.

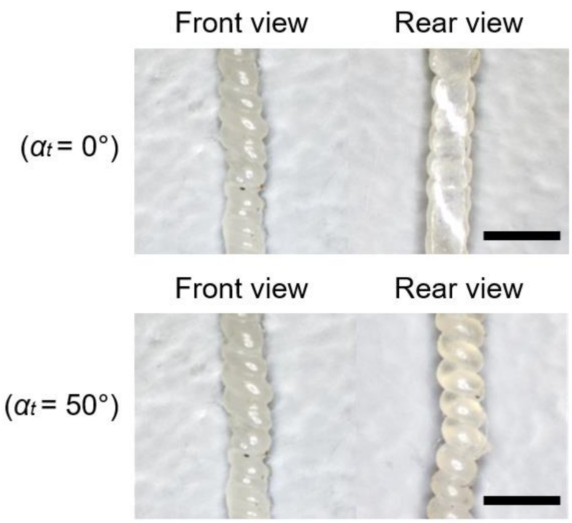

Supplementary Fig. 10. Filament printed at different tilt angle (Scale bar 1 mm).

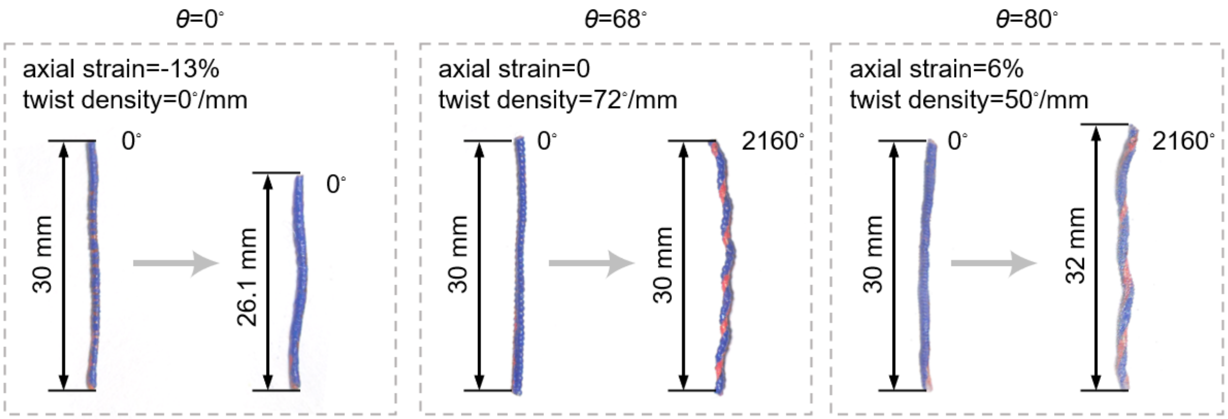

Supplementary Fig. 11. LCE filaments printed at θ = 0°, 68°, and 80° are shown before/after heating, revealing clear torsional rotation for $\theta \neq 0°$ with a θ = 0° non-twisting control. Twist density is quantified as a function of θ.

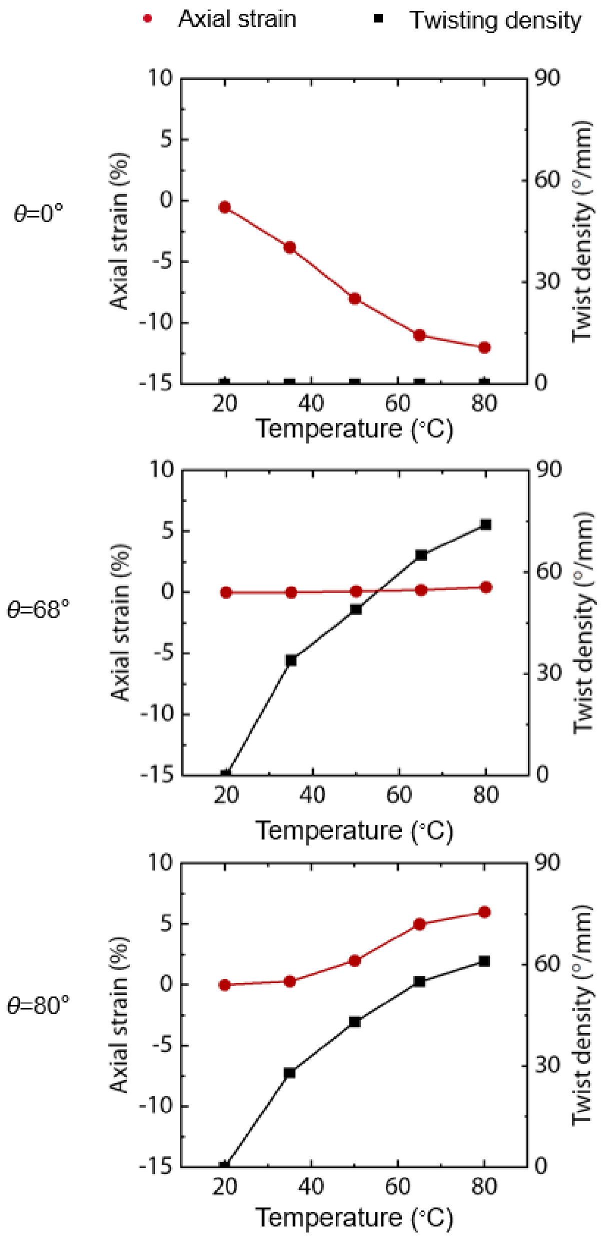

Supplementary Fig. 12. Temperature-deformation relationship of LCE filaments with different rotational angle.

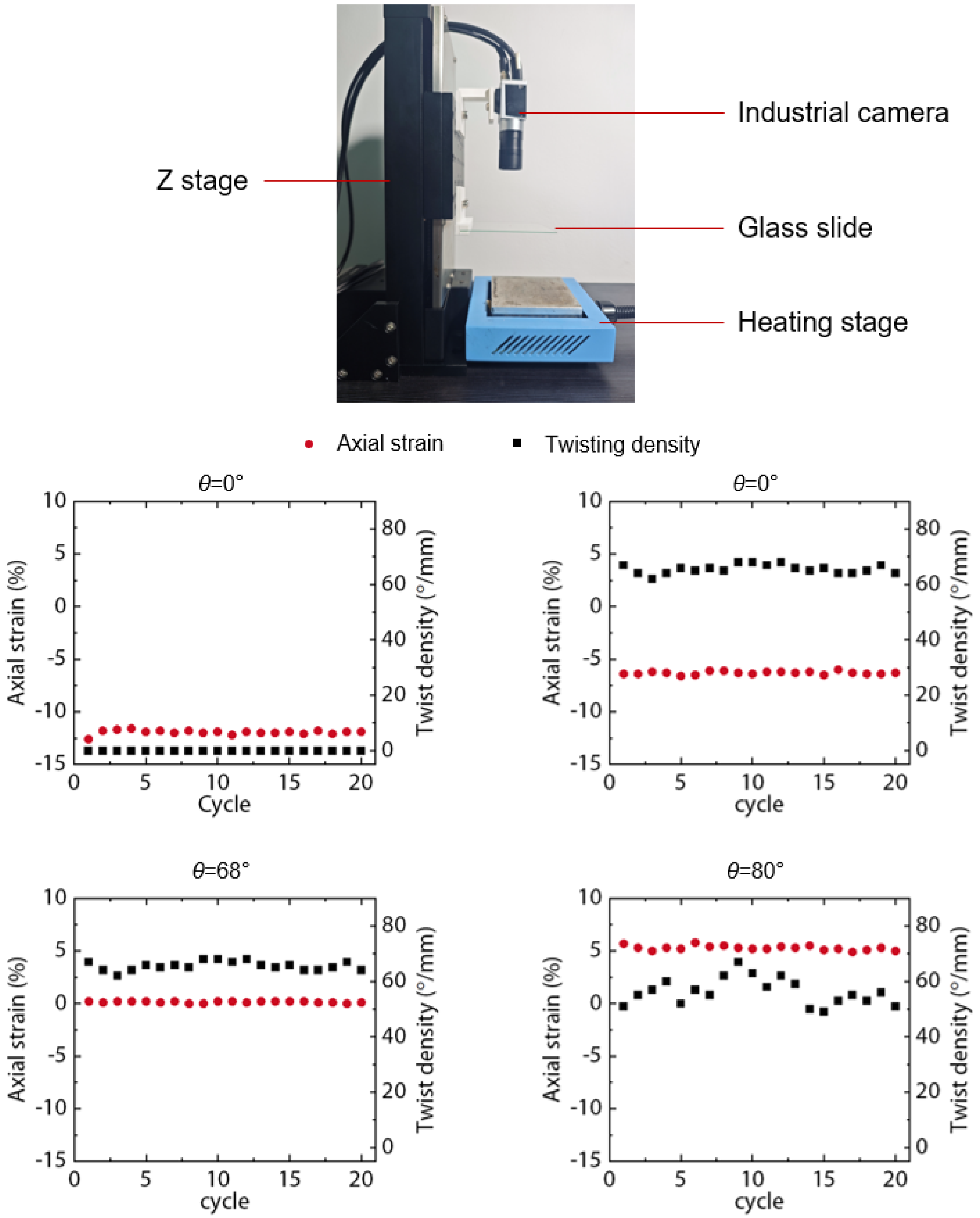

Supplementary Fig. 13. Repetitive testing demonstrates excellent repeatability.

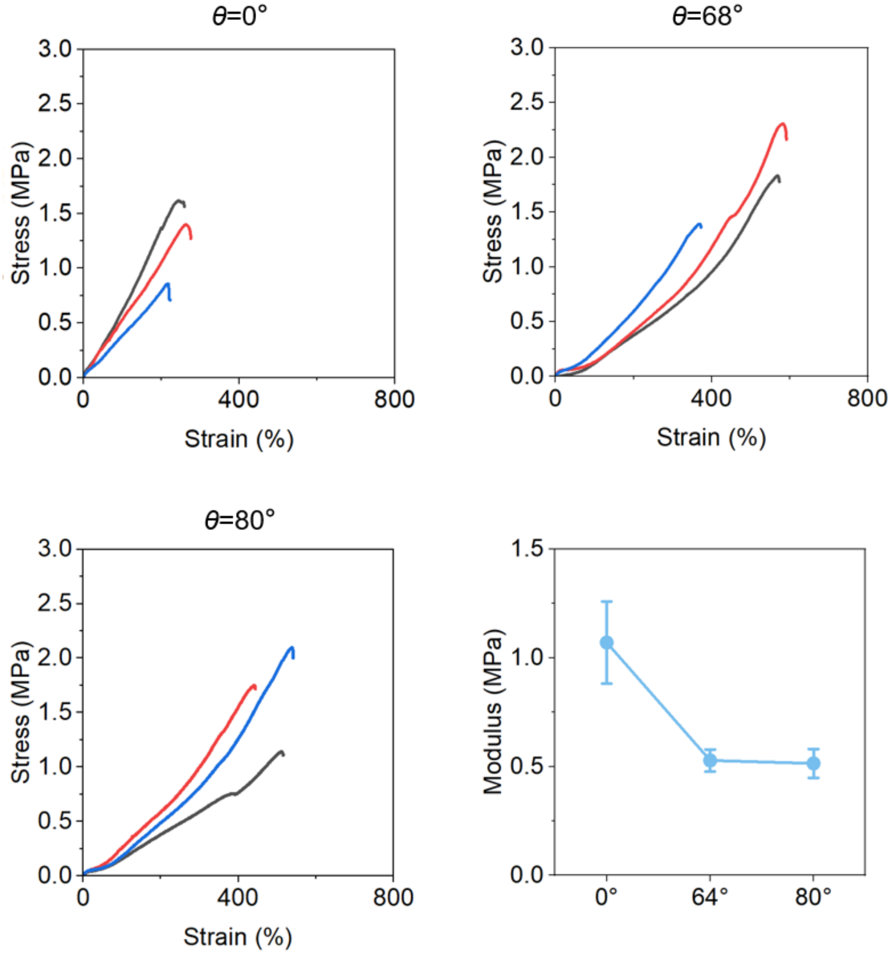

Supplementary Fig. 14. Tensile testing of LCE filaments with different rotational angles indicates that a higher rotational angle leads to a lower tensile modulus. Data are presented as mean ± SD, n = 5 independent samples.

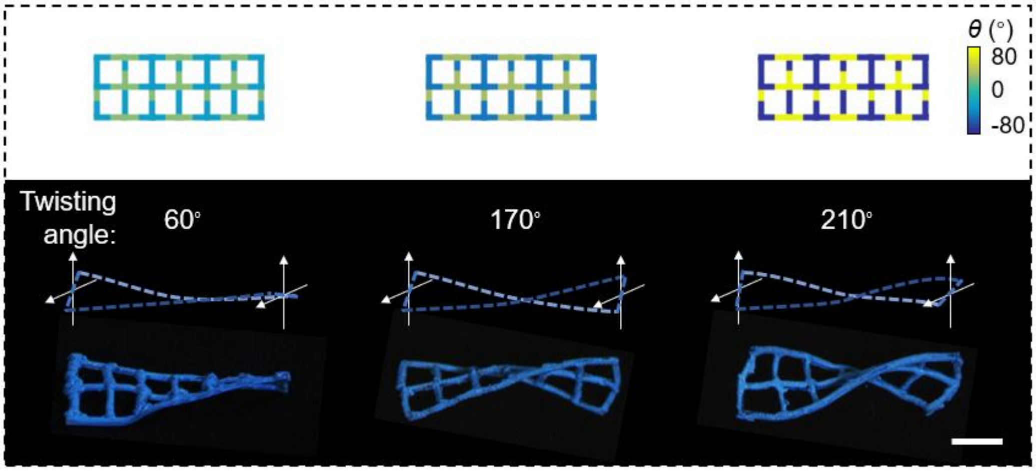

Supplementary Fig. 15. Three programmed mesh architectures exhibit distinct macroscopic twist angles after heating, demonstrating tunable bulk twisting via θ-programming. Scale bars and heating conditions are indicated.

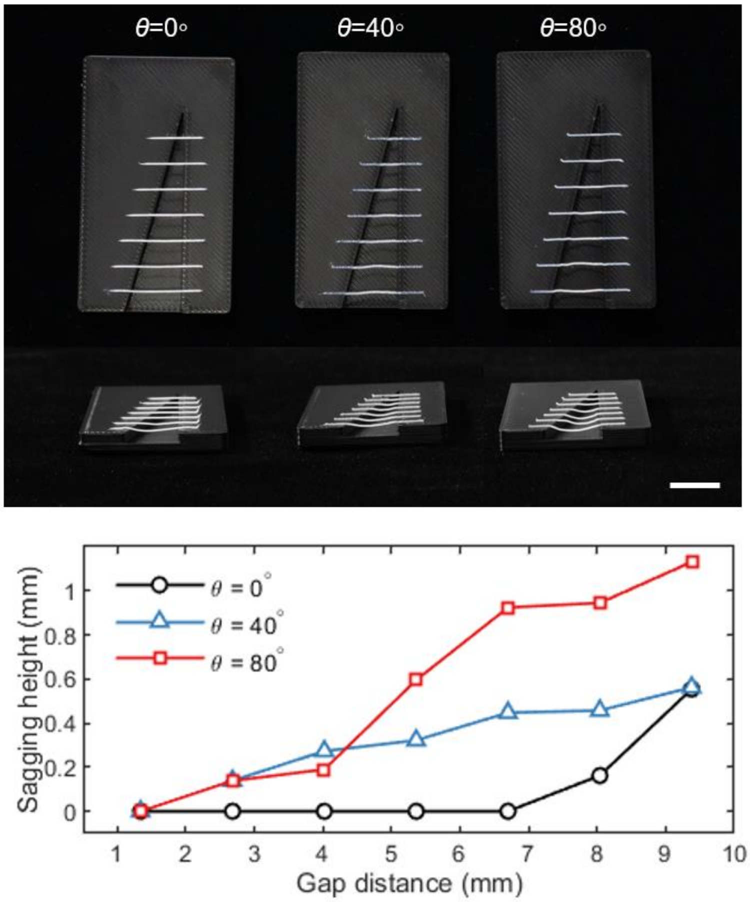

Supplementary Fig. 16. Gap-spanning performance of rotationally printed LCE filaments. Higher rotational angles result in larger sagging heights (scale bar, 5 mm).

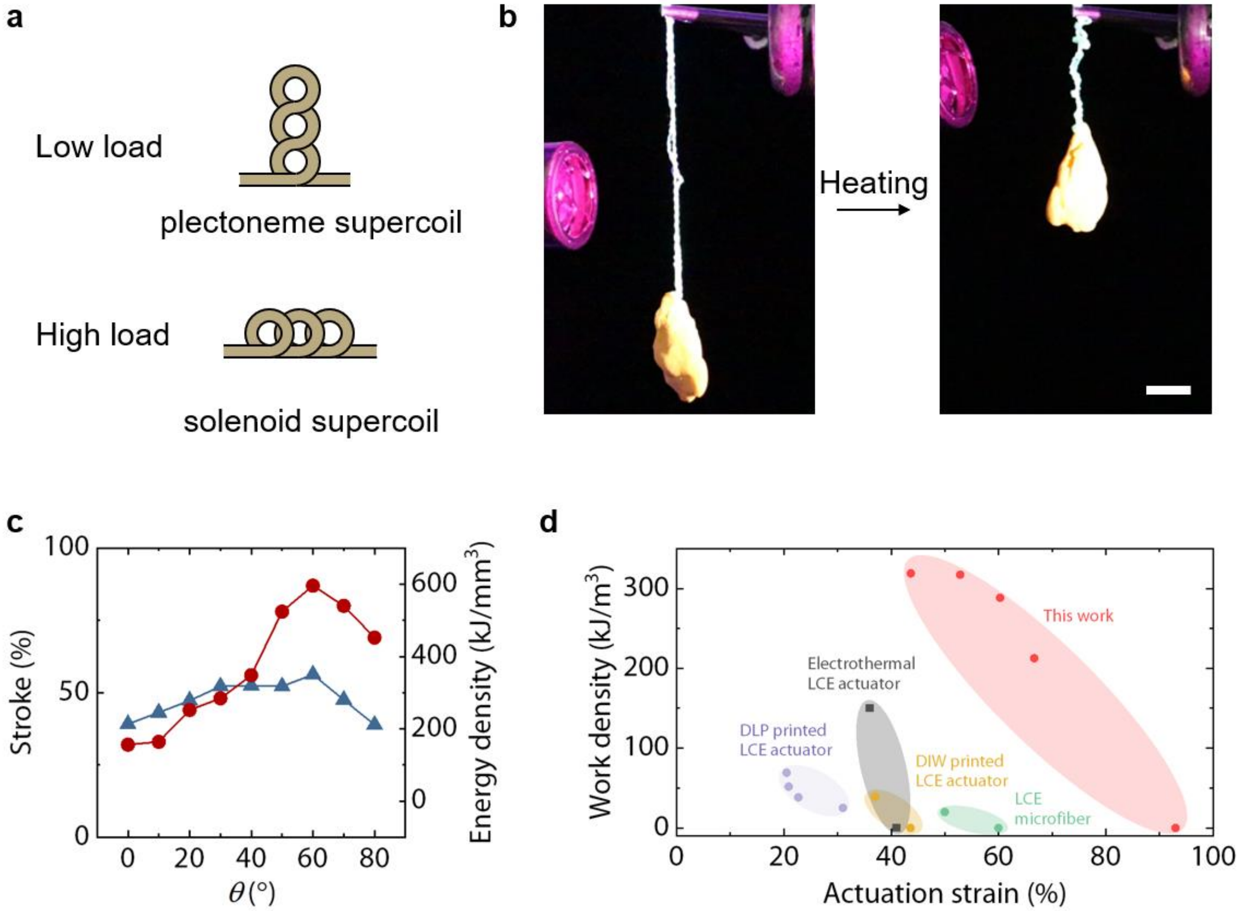

Supplementary Fig. 17. Supercoiling behavior of a rotationally printed LCE filament under constrained end rotation. (a) Under a low tensile load, the filament deforms into a plectoneme supercoil, whereas under a high load it forms a solenoid supercoil. (b) Upon photothermal heating, the supercoiled LCE filament can lift a load (2.5 g) with a large stroke (scale bar, 8 mm). (c) The actuation stroke is maximized at a printhead rotational angle of ~60°. (d) The printed supercoiled LCE exhibits a higher actuation strain and work density than previous LCE actuators fabricated by DIW 3D printing[1], DLP printing[2], mechanical stretching[3] and electrospinning[4].

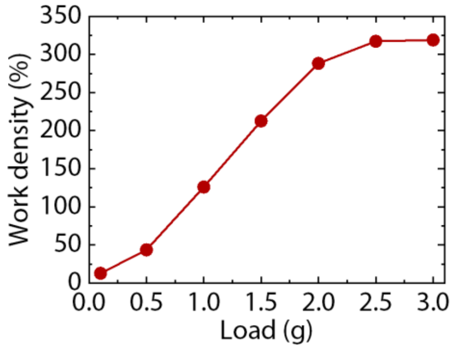

Supplementary Fig. 18. Volumetric work density as a function of lifted load.

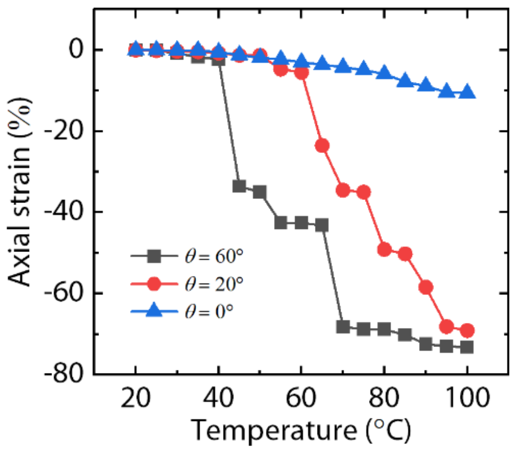

Supplementary Fig. 19. Sequential actuation of rotation-constrained LCE filament, the deformation onset has a difference of 10 °C between θ = 20° and θ = 60°.

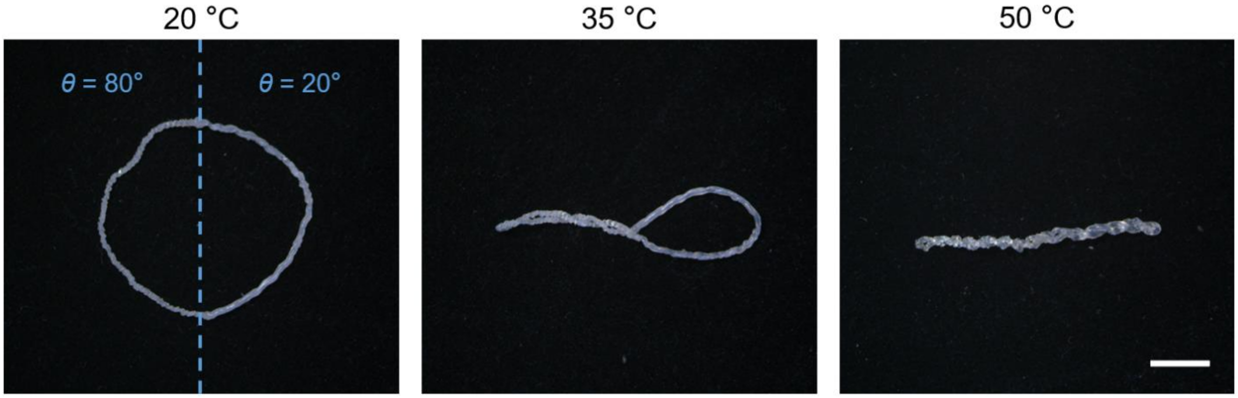

Supplementary Fig. 20. Sequential actuation of supercoiling LCE (T_{NI}=18 °C) ring with different rotational angle. (scale bar = 5 mm)

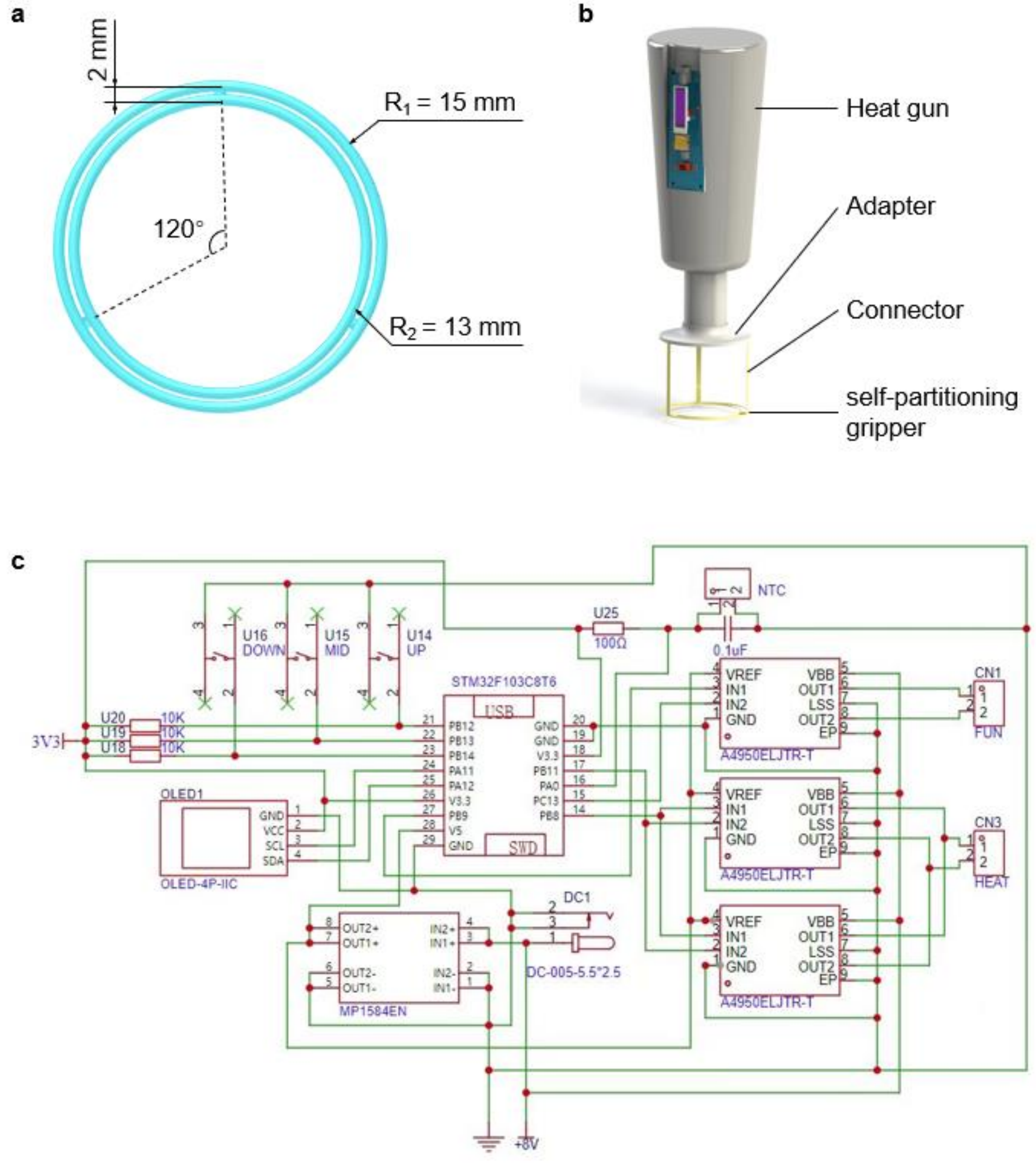

Supplementary Fig. 21. The self- partitioning gripper device. (a) Design of the gripper (outer radius, 15 mm). (b) The gripper is integrated with a custom-designed heat gun via a soft connector and adapter. (c) Schematic of the heat gun's electrical circuit.

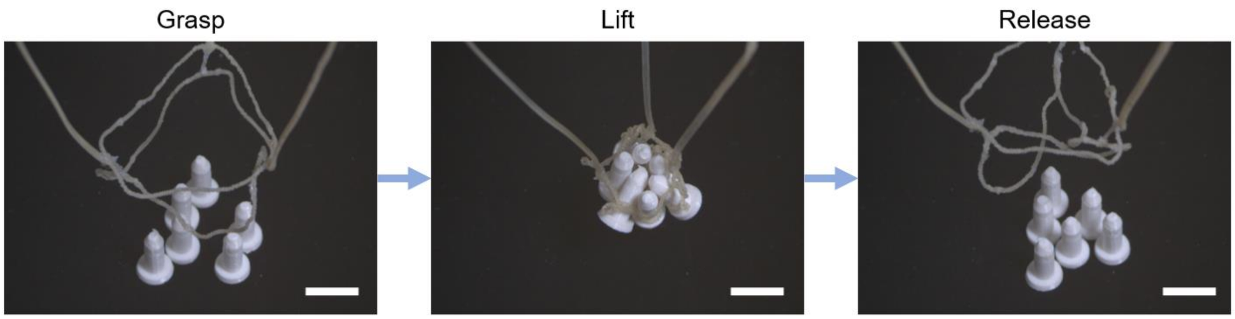

Supplementary Fig. 22. Grasping, lifting, and releasing multiple objects using a self-partitioning gripper (scale bar = 6 mm).

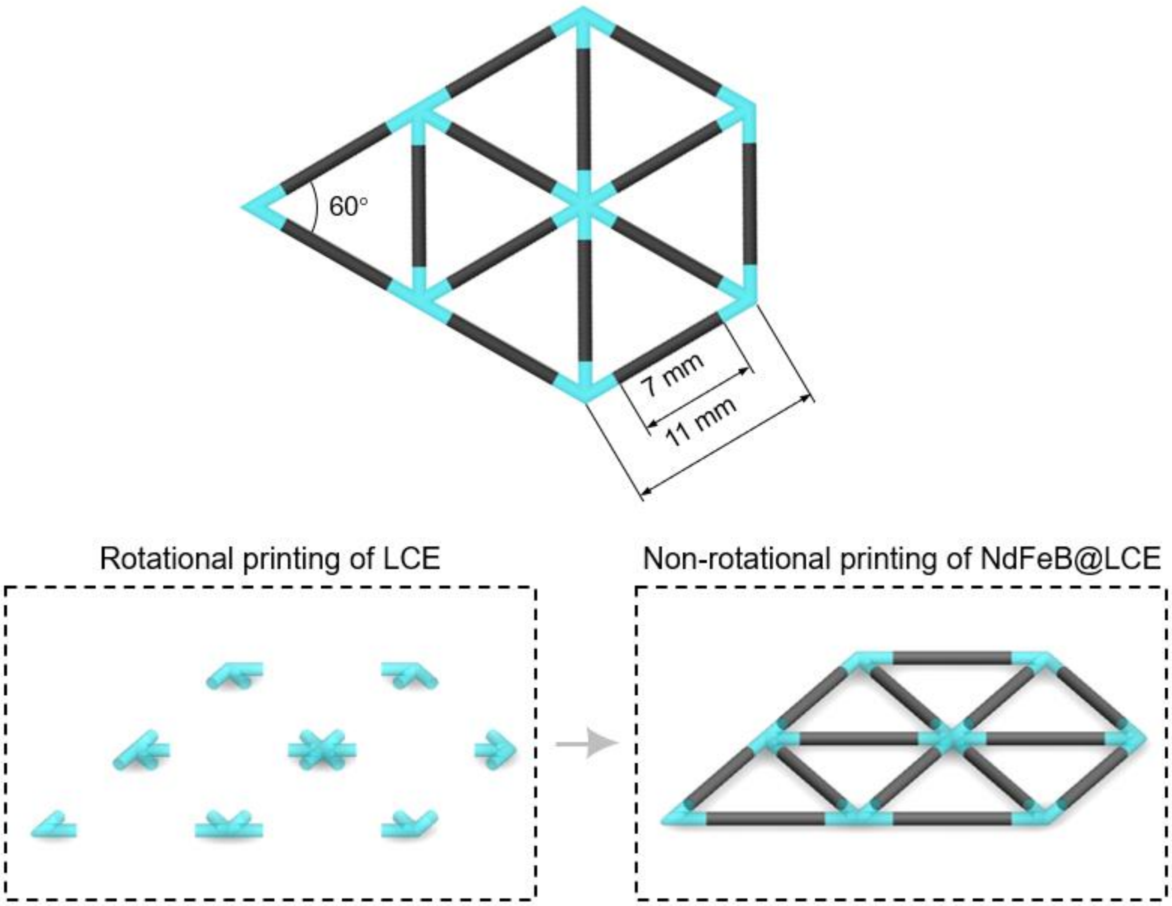

Supplementary Fig. 23. Multi-step printing of a magnetic leaf. The structure is fabricated by first performing rotational printing of an LCE filament, followed by a second step of non-rotational printing using an NdFeB-doped LCE ink.

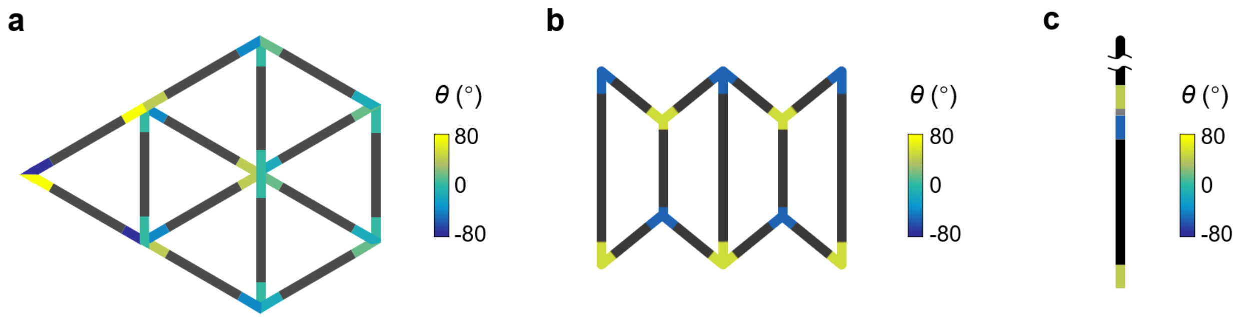

Supplementary Fig. 24. Comparison of filament rotational angles in three LCE-based actuators. (a) The LCE leaf contains filaments with varied rotational angles, whereas (b) the walking robot and (c) the robotic guidewire are composed of filaments printed with a uniform rotational angle in each device.

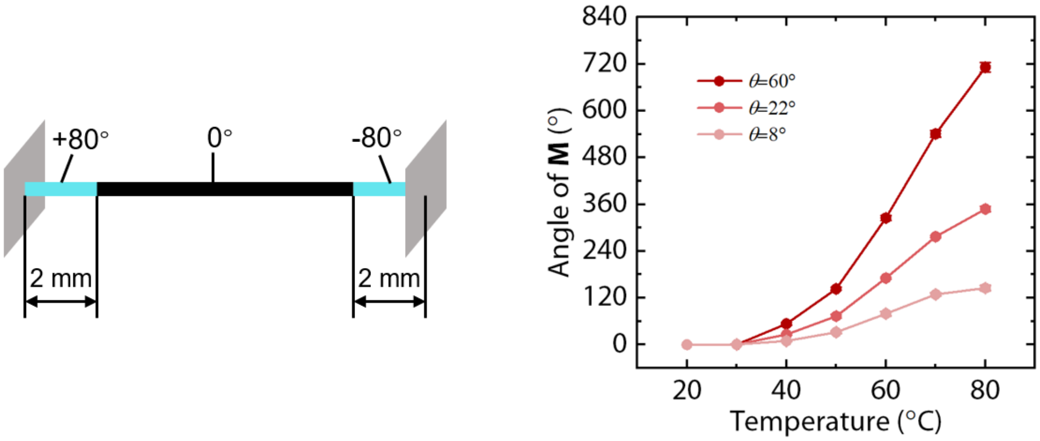

Supplementary Fig. 25. Magnetization angle of a single NdFeB-doped LCE filament under different temperature.

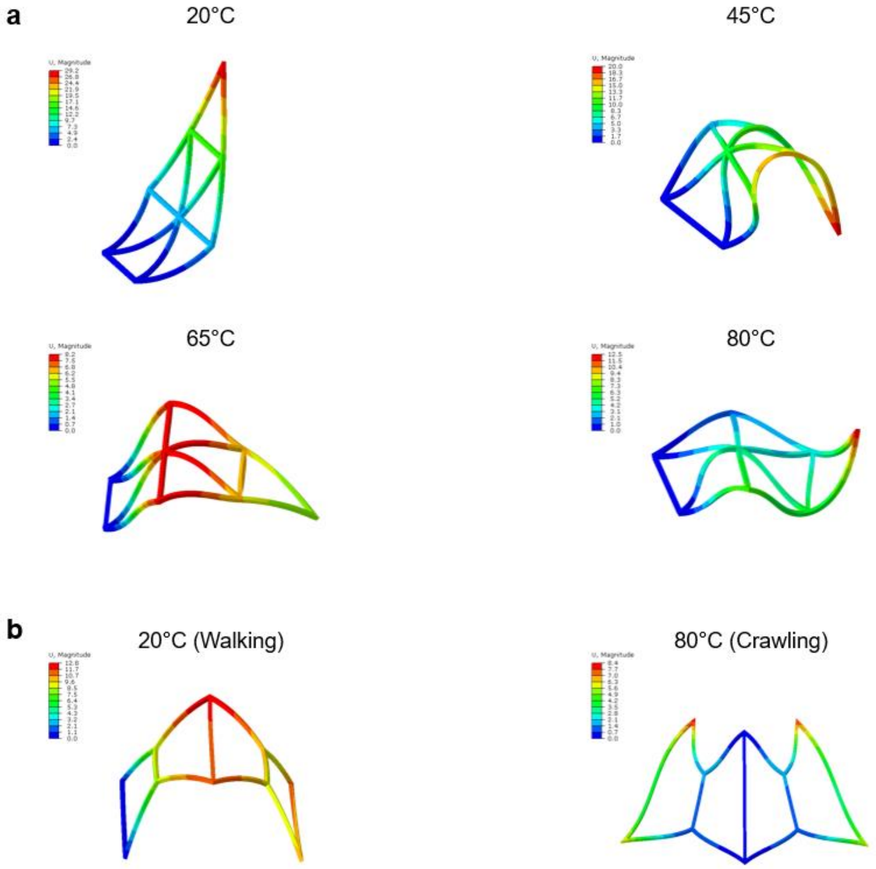

Supplementary Fig. 26. FEM simulation of volatile actuation in magnetic-LCE devices. (a) Progressive morphological changes of a magnetic-LCE leaf under varying stimuli. (b) Locomotion mode transitions in a four-segment magnetic-LCE crawler.

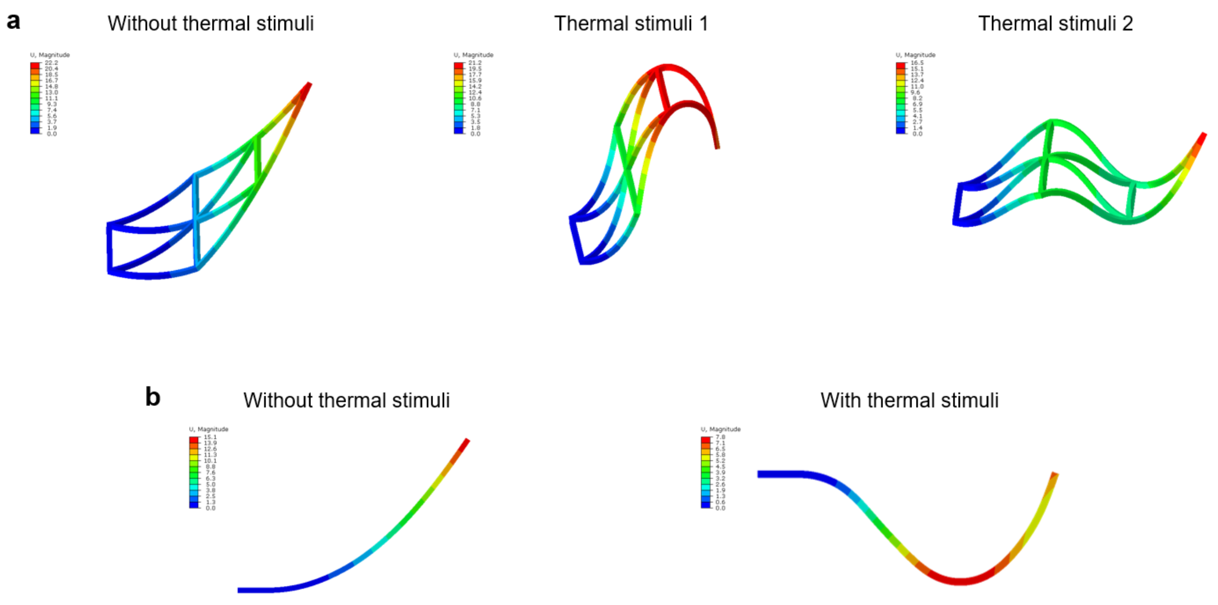

Supplementary Fig. 27. FEM simulation of nonvolatile actuation in magnetic-LCE devices. (a) Deformation of a magnetic-LCE leaf (b) Deformation induced different shape of the robotic guidewire.

Supplementary References

1 Kotikian, A., Truby, R. L., Boley, J. W., White, T. J. & Lewis, J. A. 3D Printing of Liquid Crystal Elastomeric Actuators with Spatially Programed Nematic Order. *Advanced Materials* **30** (2018).

2 Li, S. *et al.* Digital light processing of liquid crystal elastomers for self-sensing artificial muscles. *Science Advances* **7**, eabg3677 (2021).

3 He, Q. *et al.* Electrically controlled liquid crystal elastomer–based soft tubular actuator with multimodal actuation. *Science advances* **5**, eaax5746 (2019).

4 He, Q. *et al.* Electrospun liquid crystal elastomer microfiber actuator. *Science Robotics* **6**, eabi9704 (2021).

5 Raney, J. R. *et al.* Rotational 3D printing of damage-tolerant composites with programmable mechanics. *Proc Natl Acad Sci U S A* **115**, 1198-1203, doi:10.1073/pnas.1715157115 (2018).

6 Larson, N. M. *et al.* Rotational multimaterial printing of filaments with subvoxel control. *Nature*, 1-7 (2023).

7 Ren, L. *et al.* Rotational Co-extrusion 4D printing of heterogeneous filaments to enable sophisticated shape morphing. *Additive Manufacturing* **73**, 103661 (2023).

8 Ren, L. *et al.* Rotational 3D printing spruce-inspired helical structural material with tunable mechanical properties. *Chem Eng J* **490**, 151591 (2024).
