# Supplementary material for: Single-parameter programmed thermomechanical actuation via 3D-printed helical director fields in liquid crystal elastomers
Source: Nat Commun. 2026 May 15;17:6454. doi: 10.1038/s41467-026-73204-y (PMC13376412; doi:10.1038/s41467-026-73204-y)
Supplement: Supplementary file 2 — Description of Additional Supplementary Files [file 41467_2026_73204_MOESM2_ESM.pdf]

Description of Additional Supplementary Files

File name: **Supplementary Movie 1**
Description: Rotational 3D printing process for LCEs (speed x1)

File name: **Supplementary Movie 2**
Description: Controlling twist deformation through helical angle programming (speed x3)

File name: **Supplementary Movie 3**
Description: Helical angle programming of an LCE mesh yields multiple morphing modes (speed x10)

File name: **Supplementary Movie 4**
Description: Fabrication of a complex multilayer pyramid via 3D printing (speed x2)

File name: **Supplementary Movie 5**
Description: Grasping six bolts with and without self-partitioning (speed x5)

File name: **Supplementary Movie 6**
Description: Adaptive grasping of assembled and scattered LEGO bricks via self-partitioning (speed x5)

File name: **Supplementary Movie 7**
Description: Capturing multiple blackworms (*Lumbriculus variegatus*) model in tangled and untangled states (speed x3)

File name: **Supplementary Movie 8**
Description: Temperature history modulates the leaf's deformation under magnetic actuation

File name: **Supplementary Movie 9**
Description: Crossing an 80 °C zone reconfigures magnetization, converting walking to crawling (speed x1)

File name: **Supplementary Movie 10**
Description: A localized thermal spike induces segment-specific twisting in the leaf via magnetization rewrite (speed x3)

File name: **Supplementary Movie 11**
Description: NIR-triggered magnetization rewrite reprograms a robotic guidewire from L- to U-shaped actuation (speed x3)
