# Supplementary material for: Single-parameter programmed thermomechanical actuation via 3D-printed helical director fields in liquid crystal elastomers
Source: Nat Commun. 2026 May 15;17:6454. doi: 10.1038/s41467-026-73204-y (PMC13376412; doi:10.1038/s41467-026-73204-y)
Supplement: Supplementary file 14 — Transparent Peer Review file [file 41467_2026_73204_MOESM14_ESM.pdf]

nature portfolio

Peer Review File

Single-parameter programmed thermomechanical actuation in 3D-printed helical-director liquid-crystal elastomers

Corresponding Author: Dr Mujun Li

This file contains all reviewer reports in order by version, followed by all author rebuttals in order by version.

Attachments originally included by the reviewers as part of their assessment can be found at the end of this file.

Version 0:

Reviewer comments:

Reviewer #1

(Remarks to the Author)
The manuscript 'Single-parameter programmed thermomechanical actuation in 3D-printed helical-director liquid-crystal elastomers' (NCOMMS-25-85982-T) by Chen, Zhang, Li, and co-workers describes the development of a rotational 3D printing technique for main chain liquid crystalline elastomer fibers. Rotational 3D printing is a well-established technique, but has not been used directly for LCEs, which adds novelty. The authors demonstrate that by changing the tilt of the director, fibers can be programmed to undergo either contraction, twisting, or elongation. Through design of defined segments with defined director tilt, the authors are able to achieve finely programmable deformations in meshes and other objects. They also show that segments of varying tilt have different onset deformation temperatures, allowing for sequential activation of deformations. By utilizing twisting of magnetic domains, the authors further achieve multimodal deformation modes by a combination of temperature and magnetic field.

Overall, the described work provides a novel approach to LCE 3D printing and the science is convincing. Yet, the manuscript makes use of superfluous buzzwords (referring to material intelligence, evolution, biomimetic, …), which distracts from the underlying work. Moreover, the manuscripts tends to be imprecise in details (typos, chemical structures, legends, …). In its current state, the manuscript needs considerable work.

Major comments:
1) Rotational 3D printing is not new. Please add an appropriate discussion into the main text with appropriate references (i.e., from the Lewis and other groups).
2) Please provide a cut through the filament to show the distribution of the director across the filament experimentally (i.e. by TEM or by x-ray scattering).
3) What determines the temperature for onset deformation? It is not a different TNI, correct? Please describe in more detail how the onset temperature for deformation can be changed.
4) Please revise the main text, and especially the abstract, to reduce any unnecessary use of buzzwords. The work stands for itself, so it does not need any reference to intelligent materials. There is nothing intelligent (as in decision-making or adaptivity) in the produced LCEs. All responses are baked-in by design.
5) The characterization of ink and resulting polymers remains unsatisfactory. What is the length of the oligomers? Why do you use more dithiol vs. bifunctional mesogens? Please provide a Carother's analysis and better DSC data. (Also note the typos in Fig. S4 (RM82, not RM86).
6) Please carefully revise the overall manuscript to ensure readability and correctness of the details. Please make sure figures are accessible (including more detailed legends, scale bars everywhere, colored y-axes for multi-axes plots).
7) Please make the Matlab scripts and other codes openly accessible? Also the build instructions and design files, as well as wiring of the 3D printing setup, are not accessible at the moment.

Minor comments:
- Self-mimicking is not an established term.
- The magnetic LCE ink synthesis describes both the use of a Thinky mixer and ultrasonic disperser. How does that work? Please revise the text.
- Fig 2e: What are the different colors?

- Fig. 2g/h: the segments in the filament are not visible. Also, the legend is imprecise, what scale bar (2 vs. 5 mm for g and h)?
- Fig. S2: Please add scale bars. Are the 3D print files and assembly instructions openly available? Or will they be made openly available?
- Fig. S3: Please redraw chemical structures. There is also a methyl group in the central aromatic unit missing for RM82 and RM257. Please also fix the chemical structures in Fig. 1 in the manuscripts.
- Fig. S4: DSC ramps are from -50 to +150 °C. Why does Fig. S4: not show up to 150C?
- Fig. S6: Please double-check. The axial strain seems to change with filament thickness. Is the legend switched?
- Fig. S7: Please readjust y-axis for axial strain so that the black dots are visible. Please also use appropriate colors in the axis, so that it is clear which curve is for which axis.
- Fig. S8: What is the difference between the top left and top right graph? (They both seem to come from delta = 0°.
- Fig. S9: Top rights says 68°, but the bottom right graph says 64°. Please double-check.
- Fig. S10: subchart c: which curve corresponds to what axis? Subchart d: there are multiple points per 'domain'/technique, but only four references are given. Is this correct?
- Fig. S11: Is the temperature difference between the deformation onset between θ = 20° and θ = 60° not rather 20 °C instead of 10 °C?
In general, please provide more information in the figure legends. The figures are difficult to parse. Also there are quite a few typos across the manuscript. Please revise.

Reviewer #2

(Remarks to the Author)
[Editor Note: See attached for comments and for marked up Manuscript/SI documents from this reviewer]

See attached review (.docx) and comments on the PDFs.

Reviewer #3

(Remarks to the Author)
I co-reviewed this manuscript with one of the reviewers who provided the listed reports. This is part of the Nature Communications initiative to facilitate training in peer review and to provide appropriate recognition for Early Career Researchers who co-review manuscripts.

Reviewer #4

(Remarks to the Author)
This manuscript describes extrusion of liquid crystal elastomers using a rotating nozzle to control actuation properties. The filament and mesh structures that are produced are interesting, and the approach seems novel. However, the impact is lessened by the limited types of structure produced, and some of the writing/descriptions are not clear. I would recommend one major additional demonstration of the novel approach, substantial rewriting for clarity, and addressing of the following points.
1. The main major demonstration that seems necessary for publication is the demonstration of an actual 3D printed object that uses the highlighted technique of rotational printing to control local actuation. The authors include "3D-printed" in their title and only describe their 1D filaments and 2D mesh structures. 3D printing clearly offers substantial impact, and it would be an important consideration whether this technique can actually be used for 3D printing and what the limitations are. For example, can the technique enable the printing of tall structures? Can the technique be used to span gaps in a 3D lattice-type structure or similar?
2. There is a quite a bit of jargon that lacks substance, inexplicit writing, and lack of clarity in some of the main text, captions, and figures. I apologize if I misunderstand or misread some of the writing; there were many examples that I had trouble parsing, so it is possible that I overlooked detail that could have been already clarified. If that occurred, it still represents a problem with the manuscript's readability since it was difficult to follow in many instances. Some examples are laid out here. First, physical intelligence is not defined in the abstract; the definition in the introduction is clear, but as a reader, the phrase, "LCEs are promising in physical intelligence" seems nonsensical (especially with other fields like AI adopting and utilizing the phrase physical intelligence, possibly more than functional matter researchers are using it.) The phrase "selective heating of LCE segments with magnetic composites make shape programs rewritable" is not clear. It sounds like the authors are saying that they are using magnetic composites to heat the LCE, and it also sounds like the LCE is what is being reprogrammed, but it seems only the magnetic orientation is changing allowing for different response in a fixed field. what that means. Other phrases that aren't really clear include "using their intrinsic material properties and morphology" and "impart a form of embodied logic". Additionally, it seems inaccurate to denote zero net change as a "deformation mode". There was also a typo in the first part of the introduction, "ability to autonomously response", and there may be more that I missed.

Within the figures and figure captions, the following issues stood out to me. Figure 1c shows the constituents of the LCE ink but shows nothing about the "formation" of the ink as suggested by the caption. Figure 1f is not clear what the axes mean, and the labels i through iv are not explained well. In Figure 2a, the images labeled as 100 C are not clear; the text for these mentions filament cross section being asymmetric but why is it shown at 100 C? It also shows a top view of the filament it appears, not the cross section. The caption only mentions the top set of images (modifying h*). In Figure 2e, there appears to be a legend missing. In Figure 2g and 2h, I think these are supposed to be filaments shown at elevated temperatures, but the

temperature is not mentioned. The original shape is not shown for comparison.

3. The mention in the abstract that “programming imposes uniaxial order at voxel level” feels inaccurate or misrepresented here. This point is referenced in many ways throughout the manuscript; the authors emphasize that they are doing some interesting subvoxel alignment. However, I would like to know explicitly how a voxel in this case is defined. Typically, it relates to the diameter of the strand, which should be close to the diameter of the nozzle. However, it seems based on the video that the authors are significantly overextruding. Can they include in some analysis how the diameter of the strand relates to the diameter of their nozzle, especially at different rotation and print speeds? I do not see this approach being much different than simple extruding transverse to the direction of a larger printed feature. Additionally, this point does not apply to cases of LCE processing beyond printing (e.g., photopatterning surfaces.) Typos write away – a little frustrating – “ability to autonomously response”. Similarly, the authors mention that they have many modes of actuation and that typical thermal contraction at the filament level results in “only elementary deformations such as planar folding, arching or twisting.” I would like to know what deformations are not elementary since beyond contraction and extension, these seem like pretty complex modes of actuation and additionally omit Gaussian, arbitrary/organic curvature that is possible using antagonistic ordering at interfaces.

Additionally, there are previous reports of 3D printed LCE structures that can undergo multiple modes of deformation for a single monolithic print due to asymmetric and/or localized input stimulus (e.g., photoresponse). The responses can be reminiscent to what is reported here and doesn’t even require the rotating nozzle.
4. Do the grippers actually let go of the object they grip? In the videos, they only show grasping, and it would seem the untangling would be difficult. Furthermore, sequential, multiple instances of pick up and release would be more important in a demonstration of a good gripper. Otherwise, it just could be interpreted as a one time/lucky instance. It seems the authors try to address a concern like this by reported the “grasp success rate”. However, it’s not clear how this test was performed. Why would the grip success rate have a standard deviation? The only reasonable thing that I can think of is that the success rate was measured for multiple batches of grippers, but that is not mentioned.

Furthrmore for the grippers, the authors show a picture of a robotic arm, but the videos of the gripping look very unstable. Are they actually using the motors to operate the robotic arm, or are they manually picking up objects (either by unlocking the arm and moving it themselves, or by just moving the grippers around by their hand)? If it’s the latter, the photos of the robotic arm are misleading. If it’s the former, some explanation of the robotic arm or programming that results in shaky/organic movement would be needed.

Version 1:

Reviewer comments:

Reviewer #1

(Remarks to the Author)
The reviewer would like to thank the authors for their careful revision in response to the reviewer comments, specifically the added experimental details and revised language.

(Remarks on code availability)

Reviewer #2

(Remarks to the Author)
The authors did an outstanding job of addressing the critiques and criticisms from reviewers well. This is a large body of excellent work that in my opinion is now at a polished level and accurate level befitting of Nature Communications.

The responses were detailed and thorough and made a big impact on the quality of the work presented.

I support the publication of this work in its current form.

(Remarks on code availability)
The code is well commented in the G-code portion. There is no README explaining how it should be used and what setup looks like to an untrained user, but this is fairly straightforward for labs with access to compatible printers like the Ultimaker Cura.

The code is nothing special on its own, but provides clarity for groups that might want to build upon the authors' work.

Reviewer #3

(Remarks to the Author)

I co-reviewed this manuscript with one of the reviewers who provided the listed reports. This is part of the Nature Communications initiative to facilitate training in peer review and to provide appropriate recognition for Early Career Researchers who co-review manuscripts.

(Remarks on code availability)

Reviewer #4

(Remarks to the Author)
Thank you for the response from the authors. My apologies for a hastily written review; it seems I left some typos of my own in my review and left some notes that I intended to clarify. However, the authors appeared to have understood my intention and appeared to have addressed many of the major concerns.
Comment 1 – concerns are adequately addressed
Comment 2 – The authors revised to state, “Embedding complex actuation capabilities into monolithic soft materials remains a challenge for the advancement of soft robotics.”, but I don’t think that’s true. It has been clearly established by various researcher using LCEs and pneumatics that complex actuation is possible. There’s really not much value added in that regard that rotational printing enhances.
Comment 3 – concerns are adequately addressed
Comment 4 – The standard deviation was explained well in the response, but the methods within the manuscript could be updated to include a better explanation of grasp success rate and standard deviation. I would think that n = 5 would mean that you had the object picked up 5 times and measured the success rate (e.g., 4/5 = 0.8). But then a standard deviation would involve repeating that test in different instances (resetting the setup/objects as mentioned).
Comment 5 – While Video 5 appears to have been fixed, video 6 appears to be shaky still. It’s probably okay as long as the authors state explicitly. I was also just noting in the methods that it’s stated that, “it is integrated with a custom-designed heat gun”. That statement appears to reference the gripper, but perhaps the authors could clarify with a photo or description of the heat gun integrated into the robotic arm, if that is what is happening. I imagine that there is no control functionality, so it would be good to explicitly state that the gripper heating is done manually. Also, is Video 5 sped up? There’s no indication that it is.

(Remarks on code availability)
I do not feel equipped to review the code. The MATLAB code looks executable.

Open Access This Peer Review File is licensed under a Creative Commons Attribution 4.0 International License, which permits use, sharing, adaptation, distribution and reproduction in any medium or format, as long as you give appropriate credit to the original author(s) and the source, provide a link to the Creative Commons license, and indicate if changes were made.
In cases where reviewers are anonymous, credit should be given to 'Anonymous Referee' and the source.
The images or other third party material in this Peer Review File are included in the article’s Creative Commons license, unless indicated otherwise in a credit line to the material. If material is not included in the article’s Creative Commons license and your intended use is not permitted by statutory regulation or exceeds the permitted use, you will need to obtain

permission directly from the copyright holder.
To view a copy of this license, visit https://creativecommons.org/licenses/by/4.0/

Responses to Comments on "NCOMMS-25-85982-T"

We sincerely thank three reviewers for thoroughly reading our manuscript. Your constructive comments and suggestions are fully addressed point-by-point in this letter. For your convenience, the corresponding modifications are marked in blue in the revised manuscript. We believe that the concerns raised by the editor and all reviewers have highlighted areas in need of further attention and the modifications have contributed to a significant improvement of our manuscript, making it more suitable for publication in *Nature Communications*.

Reviewer # 1

General Comment

The manuscript 'Single-parameter programmed thermomechanical actuation in 3D-printed helical-director liquid-crystal elastomers' (NCOMMS-25-85982-T) by Chen, Zhang, Li, and co-workers describes the development of a rotational 3D printing technique for main chain liquid crystalline elastomer fibers. Rotational 3D printing is a well-established technique, but has not been used directly for LCEs, which adds novelty. The authors demonstrate that by changing the tilt of the director, fibers can be programmed to undergo either contraction, twisting, or elongation. Through design of defined segments with defined director tilt, the authors are able to achieve finely programmable deformations in meshes and other objects. They also show that segments of varying tilt have different onset deformation temperatures, allowing for sequential activation of deformations. By utilizing twisting of magnetic domains, the authors further achieve multimodal deformation modes by a combination of temperature and magnetic field.

Overall, the described work provides a novel approach to LCE 3D printing and the science is convincing. Yet, the manuscript makes use of superfluous buzzwords (referring to material intelligence, evolution, biomimetic, …), which distracts from the underlying work. Moreover, the manuscripts tends to be imprecise in details (typos, chemical structures, legends, …). In its current state, the manuscript needs considerable work.

Our response

We agree that rotational 3D printing has been established by pioneering groups. We have added a dedicated paragraph in the Introduction and expanded the Discussion to explicitly cite and discuss the works of Raney et al. (2018) and Larson et al. (2023). We now clarify that while those works focused on damage tolerance and multi-material sub-voxel control, our work applies this kinematic technique to LCEs to program helical director fields that enable specific phase-transition-driven deformations (supercoiling, extension) that were previously unattainable in monolithic LCE filaments.

We have carefully addressed the concerns regarding the writing style by removing superfluous buzzwords (e.g., material intelligence, biomimetic) to ensure the focus remains on the underlying science. Additionally, we have thoroughly proofread the manuscript and corrected all typos, chemical structures, and figure legends to ensure precision throughout the text."

Comment #1

Rotational 3D printing is not new. Please add an appropriate discussion into the main text with appropriate references (i.e., from the Lewis and other groups).

Response #1

We added an explicit prior-work context paragraph to discuss previous works on rotational printing and positions our contribution as LCE director-field programming and the θ - mode mapping, rather than claiming novelty of

rotation itself; this is placed in the Introduction (final paragraph) and reinforced in the Discussion (context paragraph). We added citations on rotating-nozzle studies (Refs. 43–46), which clarifies the novelty of our work and avoids overstatement.

In *Introduction*, we add:

"Recently, rotational printing has been proposed for increasing mechanical performance and programming the sub-filament architectures. For example, Raney et al. demonstrated that rotating the nozzle during DIW could impart helical alignment to short fibers within a composite matrix, enhancing damage tolerance via crack steering[46]. Larson et al. further advanced this technique to rotational multimaterial printing, achieving sub-voxel control over filament microstructure to program anisotropic stiffness and swelling behaviors in hydrogels and epoxies[47]. Similarly, Ren et al. leveraged twisted interfaces in bi-material systems to generate mismatch strain for deformation or suture-like geometries for mechanical interlocking[48,49]. However, these strategies rely on structuring heterogeneous phases—either by aligning discontinuous fillers or manipulating material distribution. The potential of this kinematic strategy to program the intrinsic molecular director field within a monolithic filament for multimodal actuation remains underexplored. Leveraging nozzle rotation to decouple the actuation mode from the printing path in a single material could significantly expand the design space of soft structures and robotics, yet a systematic methodology for such single-parameter programming is still lacking (Comparison Table 1)."

Supplementary Table 1. Comparison of rotational 3D printing strategies for programming material microstructure and functional mechanisms.

Ref	Material	Programming target	Enabled microstructure	Functional Mechanism	Application
[5]	Fiber-reinforced Composites	Discontinuous Fibers (Micro-scale)	Helically aligned short fibers	Crack steering & deflection	Impact-resistant composites
[6]	Multi-material System	Material Composition (Meso/Voxel-scale)	Spatial helical heterogeneity	Structural anisotropy via modulus mismatch	Artificial muscles; Stiffness-graded lattices.
[7]	Bi-material Systems	Interface / Structure (Meso-scale)	Twisted bi-material interface	Inter-layer mismatch strain	Shape-shifting strips; Coiling actuators.
[8]	Bi-material Systems	Interface / Structure (Meso-scale)	Suture-like helical interface	Geometric mechanical interlocking	Mechanical metamaterials; Protective structures.
This work	Monolithic LCE	Intrinsic Director Field (Molecular-scale)	Helical molecular director field	Rotated intrinsic strain axes	Soft actuator; Adaptive Grippers; Medical Guidewires.

Comment #2

Please provide a cut through the filament to show the distribution of the director across the filament experimentally (i.e. by TEM or by x-ray scattering).

Response #2

Thank you for this important suggestion. While the reviewer thoughtfully suggested TEM or X-ray scattering, we chose polarized Raman microspectroscopy to directly measure the director distribution through the filament cross section with high spatial resolution (~1 μm) and no mechanical deformation or artificial alignment artifacts.

Samples printed with three representatives programmed θ values were tested. Specifically, we prepared transverse cross-sections and conducted radial line scans from the filament center to the outer surface. The polarization-dependent Raman response of the mesogenic bands was used to extract the local director angle $\theta(r)$ as a function of normalized radius. These new data have been added as Supplementary Fig. 6, together with a brief description of the Raman analysis procedure in the Methods section.

In *Rotational printing enabled helix director field*, we add:

"To verify the director across the filament cross-section, we performed polarized Raman measurements on transverse cuts and extracted the local director angle $\theta(r)$ from radial line scans (Supplementary Fig. 6). The results show predominantly tangential (circumferential) mesogen alignment in the cross-section, with increasing anisotropy as the programmed helix angle θ increases, consistent with nozzle-rotation-induced director programming."

In *Method*, we add:

"*Polarized Raman microspectroscopy.* Transverse filament cross-sections were analyzed using a polarized Raman microscope LabRAM HR Evolution. Radial line scans recorded the polarization-dependent intensity of the mesogenic C=C stretching band at ~1600 cm^{-1}."

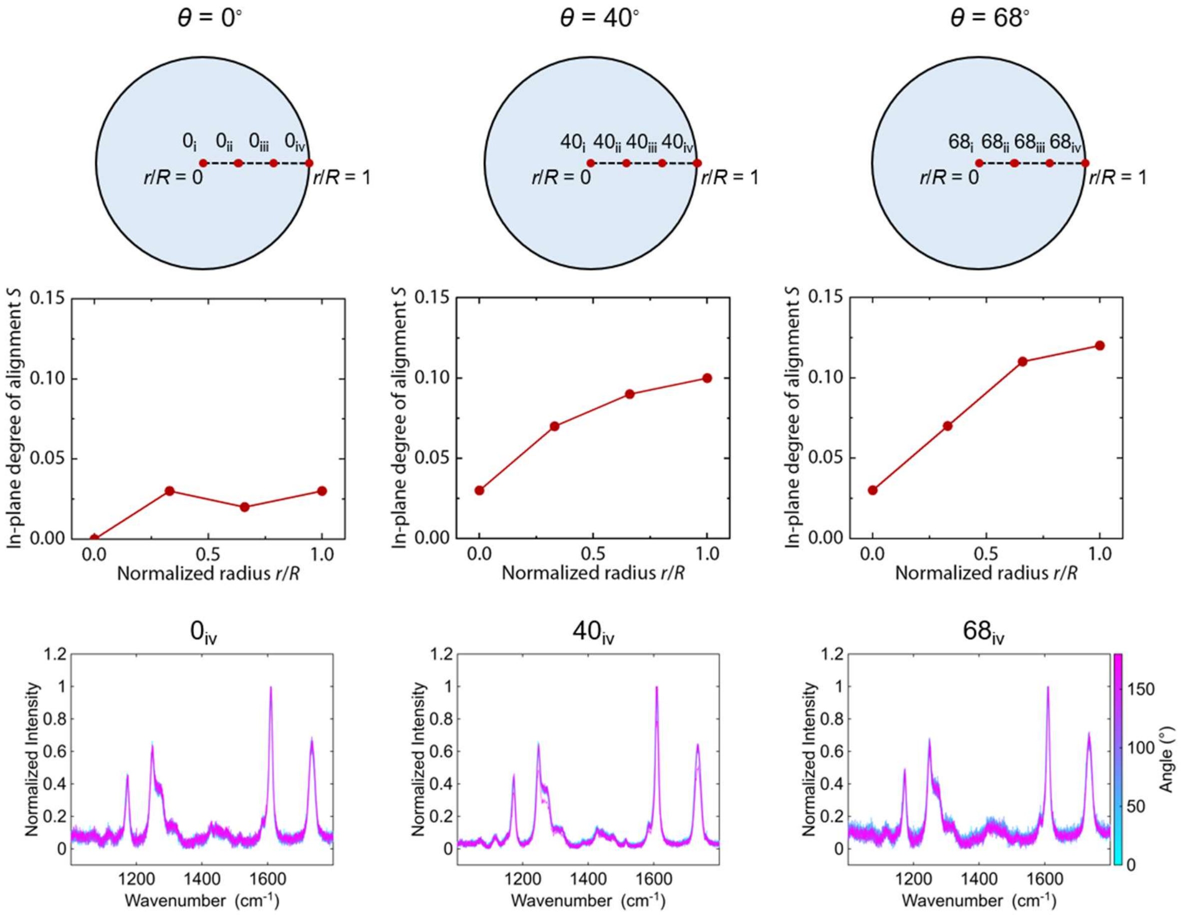

Supplementary Fig. 6. Laser Raman experiment shows higher in-plane rotation of nematic director with larger rotational angle.

Comment #3
What determines the temperature for onset deformation? It is not a different TNI, correct? Please describe in more detail how the onset temperature for deformation can be changed.

Response #3
We thank the reviewer for this insightful query. We confirm that the sequential deformation arises from the helical director field (helix angle θ**)**, not from variations in T_{NI}, as the chemical composition remains strictly uniform.
Instead, θ acts as a geometric control parameter. High θ segments possess higher stored elastic energy, effectively lowering the activation barrier for mechanical instability (Escobar & White, *Adv. Mater.* 2024). Consequently, they trigger macroscopic snap-through at lower temperatures compared to stable, low θ segments. Thus, we utilize geometry to modulate mechanical sensitivity without altering the intrinsic chemical phase transition. We have revised the Introduction and Results to explicitly distinguish the constant chemical T_{NI} from the tunable geometric onset temperature.

In *Programmable Sequential deformation for self- partitioning grippers*, we added:
"This occurs because the higher twist density effectively 'pre-loads' the segment, bringing it closer to the energy barrier required for plectoneme genesis (supercoiling). Consequently, the segment with the smaller helix angle θ_1, which presents a higher energy barrier—remains in its initial state until the temperature reaches a higher threshold, T_2, where thermal stress is sufficient to trigger deformation. This experiment unequivocally demonstrates that the temporal sequence of actuation is encoded within the filament's geometry. Such differential thermal sensitivity enables stepwise actuation, governed by the elastic energy stored in the helical director field (Supplementary Fig. 19, Supplementary Fig. 20). Specifically, the pre-encoded helical director field in high-θ segments acts as a geometric pre-load, storing higher elastic energy that lowers the macroscopic energy barrier for mechanical instability[52]. To demonstrate this capability, we designed a self-partitioning gripper that undergoes programmed deformations to grasp either single or multiple objects."

Comment #4
Please revise the main text, and especially the abstract, to reduce any unnecessary use of buzzwords. The work stands for itself, so it does not need any reference to intelligent materials. There is nothing intelligent (as in decision-making or adaptivity) in the produced LCEs. All responses are baked-in by design.

Response #4
We agree with the reviewer that the work stands on its own without the need for buzzwords. Accordingly, we have removed anthropomorphic phrasing (e.g., 'intelligence,' 'embodied logic,' 'evolution/mutation') from the Title, Abstract, Introduction, and Discussion. These terms have been replaced with deterministic, technical descriptions focused on design-encoded director programming and single-parameter actuation selection. This ensures the text accurately reflects the baked-in nature of the LCE response.

Revised *Title*:
Single-parameter programmed thermomechanical actuation via 3D-printed helical director fields in liquid crystal elastomers

In *Abstract*, we revised:

"Embedding complex actuation capabilities into monolithic soft materials remains a challenge for the advancement of soft robotics. Liquid crystal elastomers (LCEs) offer intrinsic stimuli-responsiveness, yet conventional extrusion-based 3D printing restricts them to uniaxial alignment at filament level, limiting their utility to simple contraction modes. Here we introduce a rotational 3D printing approach that embeds a helical director field within LCE filaments, enabling multimodal actuation controlled by a single fabrication parameter: the helix angle (θ). Tuning θ programs each filament to contract, elongate, twist or remain macroscopically invariant when heated, decoupling actuation logic from device geometry. Spatial gradients in θ create a hierarchy of activation temperatures, yielding sequential shape changes under uniform heating. Localized heating of the magnetic-LCE composite segments allows their magnetic domains to be reoriented, making the shape programs rewritable and enabling switchable volatile and non-volatile memory. We demonstrate these capabilities in self-partitioning grippers, multimodal/color robots and reprogrammable guidewires that perform multi-step or adaptive tasks without external circuitry. By encoding actuation modes, deformation sequences, and memory in a single parameter, this approach establishes a paradigm of material-encoded programmability and points toward monolithic soft robots and reconfigurable structures."

In *Introduction*, we revised:

"Stimuli-responsive materials provide a pathway to soft machines capable of complex, untethered shape transformations. By leveraging intrinsic thermomechanical properties rather than external motors, these systems can achieve sophisticated behaviors such as self-folding, locomotion, and adaptive gripping[1-5]. Rather than relying solely on external computation or rigid control algorithms, stimuli-responsive systems leverage material architectures to realize behaviors such as self-shaping[6-10], self-sensing[11-13], and adaptive response[14-16] in situ. A key challenge in this paradigm is creating actuators that can exhibit desired modes of behavior, enabling machines with multi-modal, adaptive and even reconfigurable response[2,3,17]. Recent advances in smart materials have started to address this: for example, liquid crystal elastomers (LCEs) can be programmed to undergo large, predetermined shape changes (e.g. contraction, bending, or twisting) when stimulated by heat or light[18-20]. By encoding molecular alignment within an LCE, researchers can impart a form of encoded anisotropy—the material itself directs how it will move under stimuli, contributing to the system's overall functionality[6-9,14,15]. However, traditional programming methods are typically tailored for simple deformation mode (e.g., mechanical stretching[21-23]), limited to thin-film LCEs (e.g., surface patterning[24-26]), or limited complexity and materials (electrical/magnetic field alignment[19,27,28]). Creating devices that can achieve complex actuation modes usually requires combining separate actuators or complex architectures, adding design and fabrication complexity that run counter to the concept of monolithic, integrated design.

3D printing has emerged as a powerful approach … Ideally, a single monolithic LCE element that can be programmed with any desired deformation mode in situ would offer greater adaptability, simplifying the creation of responsive machines with rich behavior repertoires."

We revised the section title *DNA inspired evolution and mutation* to *Programmable volatile and non-volatile configuration memory*. In this section, we revised:

"To realize versatile and reconfigurable soft robotics, materials are required to exhibit tunable memory capabilities, allowing for both reversible shape shifting and reprogramming of their actuation behaviors. Here, we introduce magnetic composites not as a primary actuator, but to enable reprogrammable volatile and non-volatile memory on top of the printed helical director fields. The composite structure comprises 10 discrete LCE segments arranged in series, each functioning as a tri-section composite unit. The two terminal ends of each segment are LCE components fabricated via multi-step rotational printing (Supplementary Fig. 23), imparted with

specific helix angles θ so that they undergo predetermined multi-angled twisting deformations upon thermal activation (Supplementary Fig. 24a). The central portion of each segment is a magnetized NdFeB–LCE composite. When heat is applied, the end LCE components twist, causing the central magnetic section to rotate and thereby reorient its magnetic domains (Figure 5a, Supplementary Fig. 25).

By tailoring the helix angles of the terminal LCE segments based on the twist–deformation profile from the printing process, the rotation angle of each central magnetic segment can be precisely controlled, effectively programming the leaf's magnetic response behavior. The LCE composite also exhibits dynamic, temperature-dependent configuration changes. An increase in external temperature induces a coordinated global rotation of all segments, which is reversed when the temperature decreases. This reversible behavior indicates a volatile memory effect, as the thermally induced configuration change "vanishes" upon cooling (Figure 5b). As shown in Figure 5c and Supplementary Fig. 26a and Supplementary Video 8, gradually raising the ambient temperature from 20 °C to 100 °C causes the LCE segments to progressively reorient, resulting in a spectrum of different deformed shapes when the structure is subsequently exposed to a magnetic field. This volatile reconfiguration capability facilitates adaptive robotic functions. Drawing on the mimic octopus (*Thaumoctopus mimicus*), which simultaneously alters body shape and color[57,58], we built a four-segment crawler with different colors on each side (Figure 5d, Supplementary Fig. 24b). At 20 °C the robot walks with its white surface uppermost; upon entering an 80 °C zone the heated segment twists, flipping to its blue side and altering both magnetization and gait. Successive flips progressively convert a white, walking robot into a blue, crawling one, demonstrating multi-modal adaptation encoded entirely in the material (Figure 5e, Supplementary Fig. 26b, Supplementary Video 9).

Conversely, a sudden localized thermal stimulus can induce a non-volatile change in the LCE leaf's configuration that persists even after the stimulus is removed. In other words, a rapid, concentrated heating of a specific region produces a locked reorientation in those LCE segments, and this altered state remains "memorized" in the leaf's magnetic response after cooling, exemplifying a non-volatile memory effect (Figure 5f). The sequence illustrated in Figure 5g and Supplementary Video 10 demonstrates these principles in detail. Initially, without any induced reprogramming, the structure remains consistent and stable as the strength of an applied magnetic field is increased. Next, under a moderate magnetic field of 5 mT, segments 9 and 10 are rapidly heated with a heat gun, inducing local deformations that produce an approximately 180° rotation in their magnetic domain orientation. When the magnetic field is subsequently increased to 40 mT, the tip of the structure bends downward as a result of the earlier thermal reprogramming. Finally, further localized heating of the front and middle segments causes the structure to deform into an "N"-shaped configuration under the magnetic field (Supplementary Fig. 27a). Throughout these adaptive transformations, the LCE structure demonstrates an ability to adjust its torsional configuration and magnetic orientation in response to changing thermal and magnetic conditions."

Revised Figure 5

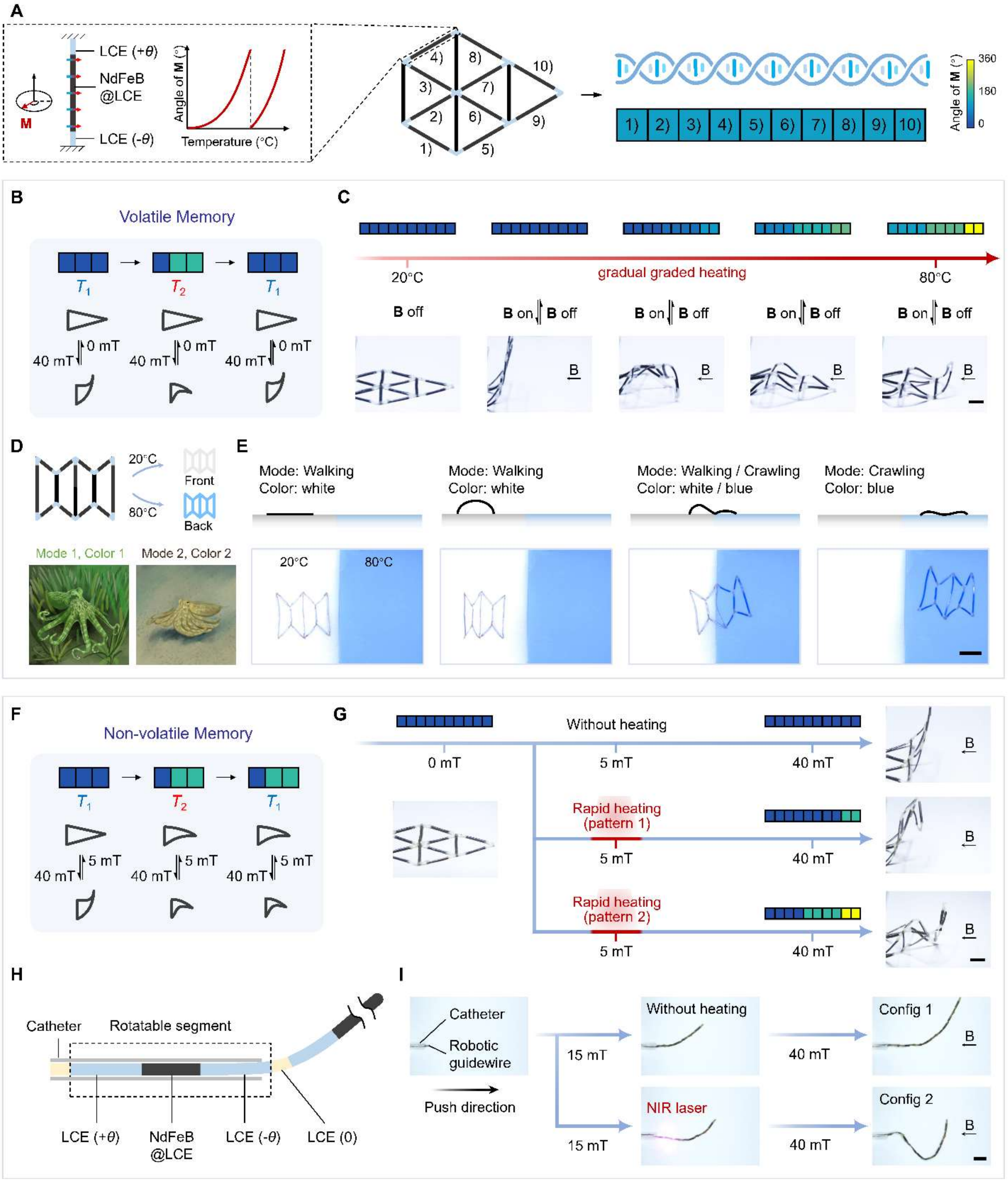

Figure 5. Magnetic-LCE structures with programmable volatile and non-volatile memory. (a) Each LCE leaf is composed of 10 discrete LCE segments, each containing two types of LCE sub-structures with opposite twist and a magnetic composite substructure, resulting in rotation of magnetization under thermal actuation. (b) Increasing the external temperature induces a coordinated global rotation in the LCE leaf (collective twisting of all segments), which disappears upon cooling—demonstrating volatile memory. (c) This temperature-driven reconfiguration leads to varied deformation profiles when the leaf is subjected to a magnetic field. (scale bar=5 mm) (d) Four segment LCE crawler demonstrating color and gait switching enabled by volatile reconfiguration. (e) Robot segments flip from white to blue as they traverse an 80 °C zone, transforming a walking robot into a crawling one by simultaneously reconfiguring its magnetization. (scale bar=5 mm) (f) Under a constant low magnetic field (5

mT), a localized spike in temperature triggers a locked reorientation in the leaf (twisting in specific segments), which is retained after the leaf cools, indicating non-volatile memory of the magnetic response. (g) Under a low magnetic field (5 mT), rapidly heating certain segments induces local deformations that rotate their magnetic domains by ~180°. Under a stronger magnetic field (40 mT), these reprogrammed states cause the leaf to deform into different shapes (scale bar=5 mm). (h) Enabled by thermal reprogramming, a robotic guidewire is designed with reprogrammable magnetic actuation when it "grows" from a catheter. (i) Using a near-infrared (NIR) light-induced reprogramming, the robotic guidewire's magnetic actuation is reprogrammed from an "L" shape to a "U" shape (scale bar=5 mm).

Comment #5

The characterization of ink and resulting polymers remains unsatisfactory. What is the length of the oligomers? Why do you use more dithiol vs. bifunctional mesogens? Please provide a Carother's analysis and better DSC data. (Also note the typos in Fig. S4 (RM82, not RM86).

Response #5

We appreciate the reviewer's comments on the ink characterization.

1. Oligomer Length: Using a Carothers analysis for the EDDET + RM82/RM257 thiol–Michael oligomerization with $r=N_{C=C}/N_{SH}=0.8$ and assuming near-complete conversion of the limiting acrylate groups (p→1), we obtain $\bar{X}_n = (1+r)(1-r) \approx 9$. To further support this estimate, THF-GPC (PS standards) gives PS-equivalent M_n=6.9, M_w=14.0 kDa, and Đ=2.03 (Supplementary Fig. 4). The calculation and GPC details have been added to the revised Supporting Information.
2. Stoichiometry (Excess Dithiol): We used excess dithiol ($r = [\text{Acrylate}]/[\text{Thiol}] = 0.8$) for two main reasons:
   - Processability: To limit the molecular weight and prevent premature gelation, ensuring the ink remains a printable liquid.
   - Functionalization: To generate thiol-terminated oligomers. These pendant thiol groups are necessary to react with the allyl groups of the crosslinker (TATATO) during the second-stage UV photocuring (thiol-ene reaction).
3. Data Updates: We have corrected the typo (RM86 to RM82) in Supplementary Fig. 4(original Supplementary Fig. 5). We also re-evaluated the thermal properties using new DSC measurements to provide clearer data on T_g and T_{NI}, which are updated in Supplementary Fig.5 (original Supplementary Fig. 5).

In *Supplementary Text*, we added:

Theoretical Analysis of Oligomer Length

To estimate the length of the synthesized LCE oligomers, a theoretical analysis based on the Carothers equation was performed. The synthesis involves a base-catalyzed thiol-Michael addition between the dithiol (EDDET) and diacrylate mesogens (RM82 and RM257). The allyl-containing crosslinker (TATATO) is inert under these conditions and does not participate in the initial oligomerization.

The molar ratio of functional groups is calculated as follows:

Thiol groups (N_{SH}): $1.0\ \text{mol (EDDET)} \times 2 = 2.0$

Acrylate groups ($N_{C=C}$): $(0.6\ \text{mol (RM82)} + 0.2\ \text{mol } (RM257)) \times 2 = 1.6$

The stoichiometric imbalance ratio r is:

$$r = \frac{N_{C=C}}{N_{SH}} = 0.8$$

Assuming complete conversion of the limiting acrylate groups ($p \rightarrow 1$), the number-average degree of polymerization $\bar{X}_n$ is:

$$\bar{X}_n = \frac{1+r}{1-r} = 9$$

This calculation indicates that the ink is composed of thiol-terminated oligomers with an average length of approximately 9 structural units. Consistent with this prediction, THF-GPC (PS standards) shows an oligomeric molecular-weight distribution with PS-equivalent M_n=6.9 kDa, M_w=14.0 kDa, and $Đ$=2.03 (Supplementary Fig. 4). The excess thiol groups serve to end-cap the oligomers, preventing gelation during synthesis and providing reactive sites for the subsequent UV-initiated crosslinking with TATATO.

In *Rotational printing enabled helix director field*, we added:

"In this study, we employed a main-chain LCE (Figure 1c, Supplementary Fig. 3) with a molecular weight of M_w=1.4×10^4 (Supplementary Section 1, Supplementary Fig. 4). Notably, T_{NI} of this material can be tuned by adjusting the ratio of RM257 to RM82 (Supplementary Fig. 5)."

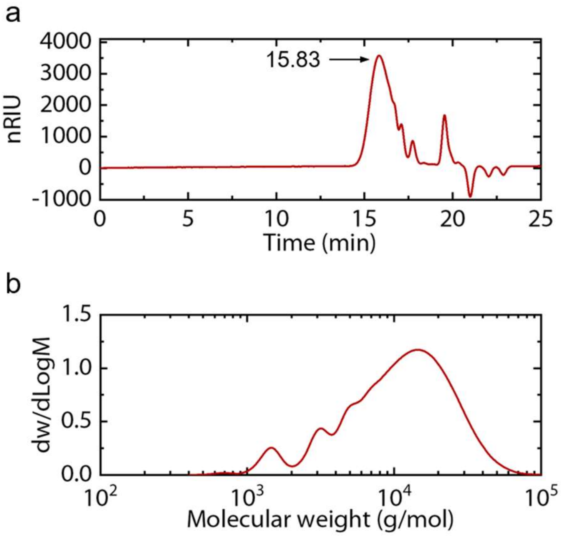

Supplementary Fig. 4. THF-GPC characterization of the LCE oligomer ink. (**a**) Representative refractive-index (RI) chromatogram showing the main elution peak at 15.83 min. (**b**) Corresponding molecular-weight distribution (polystyrene calibration), yielding PS-equivalent M_n=6.9×10^3, M_w=1.4×10^4, and Đ=2.03.

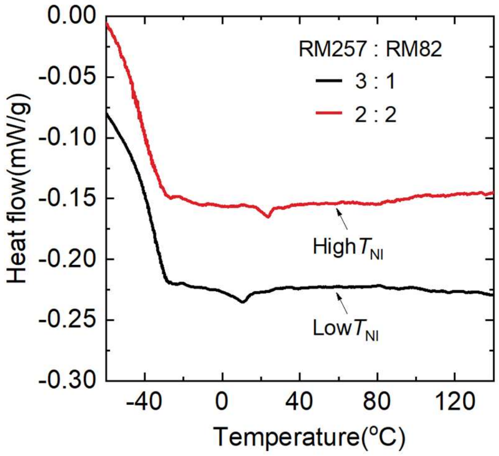

Supplementary Fig. 5. DSC curves of LCE samples with different RM257:RM82 monomer ratios, showing that the phase transition temperature varies with composition.

Comment #6

Please carefully revise the overall manuscript to ensure readability and correctness of the details. Please make sure figures are accessible (including more detailed legends, scale bars everywhere, colored y-axes for multi-axes plots).

Response #6

(1) We have expanded the figure legends to provide more descriptive explanations of the experimental conditions and observed phenomena, allowing the figures to be better understood independently of the main text.
(2) We have double-checked all micrographs and photographs to ensure that scale bars are clearly visible and consistently labeled in all panels (including insets).
(3) We have colored the axis lines, ticks, and labels to match the corresponding data curves for Figure 2 b and c.

Comment #7

Please make the Matlab scripts and other codes openly accessible? Also the build instructions and design files, as well as wiring of the 3D printing setup, are not accessible at the moment.

Response #7

We thank the reviewer for highlighting the importance of accessibility and reproducibility. We have now uploaded the MATLAB scripts for G-code generation. We have also included a comprehensive build guide, the complete set of CAD design files (in STEP formats), and a detailed wiring schematic in the same repository.

We added *Code Availability*:

"The custom MATLAB scripts used for G-code toolpath post-processing, alongside the comprehensive hardware build guide and CAD design files (.STEP format) necessary to replicate the rotational 3D printing setup, are open-source and freely available in the public Zenodo repository under the accession DOI: https://doi.org/10.5281/zenodo.18671953."

Reviewer # 2

Comment in Review Summery

Key Results

The authors applied a rotational 3D printing technique to control liquid crystal elastomer (LCE) fiber orientation to elicit controlled thermal deformation behavior. Previously, deformation in 3D printed LCEs has been dictated by uniaxial fiber orientation due to the shear experienced in the thixotropic feedstock during extrusion. To overcome this, this work utilizes an extrusion nozzle at which rotates at a prescribed angular velocity, introducing localized radial patterning. Using this technique, the authors modified the nematic - isotropic thermally triggered deformation of the extruded filament. Bulk deformation behavior of filaments can be further controlled by combining multiple sections of discrete fiber orientation angles. Spatially prescribed deformation behavior enabled fabrication of simple 2D lattices with varied deformation modes. Furthermore, the authors fabricated stimuli responsive functional "gripper" with two deformation modes that are triggered at different temperatures. This is accomplished by creating a mismatch in phase transition temperature with varied angles. Lastly, a second method of stimuli responsive deformation was added in the form of magnetic particles.

Response

Thank you for reading and reviewing our manuscript.

Comment #1: Validity

Backed by extensive solid mechanics theory. Their method of quantifying "twisting" (phi) leaves something to be desired.

i) Figure 2:

a. Explain the heating segment of the figure.

b+c. Consider color coding each axis for clarity

d. improve resolution

g. needs a pre-deformation image, indicating where the different rotation zones are. Where are the green and yellow markers you are talking about? The provided figures are not green and yellow. Add scale bar.

h. Same issues as g

ii) The tensile data presented in SI Fig. 9 is inconsistent with LCE literature using the same materials system replicated from other work. Namely, tensile strength is an order of magnitude greater and ultimate strain double that documented in previous literature. If this is indeed the case, then this should be a major focus of the paper, as this is unprecedented performance in LCE materials. However, the Young's Moduli presented in part d are inconsistent with the supplied tensile data. Figure SI 10 suggests this technique can achieve impressive actuation strains and work densities versus previous literature, yet it is not referenced in manuscript. The incongruities bring into question the robustness of the experimental approach related to materials characterization.

Response #1

(i) Thank you for your careful read and evaluation. In response to your question:

a. We have clarified the description of the heating segment in the revised caption and main text. This segment illustrates the thermal actuation of LCE filaments printed with different nozzle tilt angles (α_t). Specifically, the images at 100 °C demonstrate how optimizing α_t minimizes shear-induced asymmetry, resulting in stable helical deformations rather than uncontrolled distortion upon heating.

b+c. We have revised the multi-axis plots in Figure 2c. The axes and corresponding data points are now color-coded (e.g., red for axial strain, black for twist density) to enhance readability and clarity.

d. We have replaced the raster panels in Figure 2d with high-resolution images to improve visual quality.

g. We have updated Figure 2g to include pre-deformation images alongside the actuated states. We also added explicit labels for the different rotation zones (L_1, L_2) and scale bars. Furthermore, we have corrected the text in the manuscript to strictly match the colors of the markers used in the figure (blue and white) to eliminate any inconsistency.

h. Similar to panel (g), we have added pre-deformation images and scale bars to Figure 2h to clearly show the filament configuration before and after actuation.

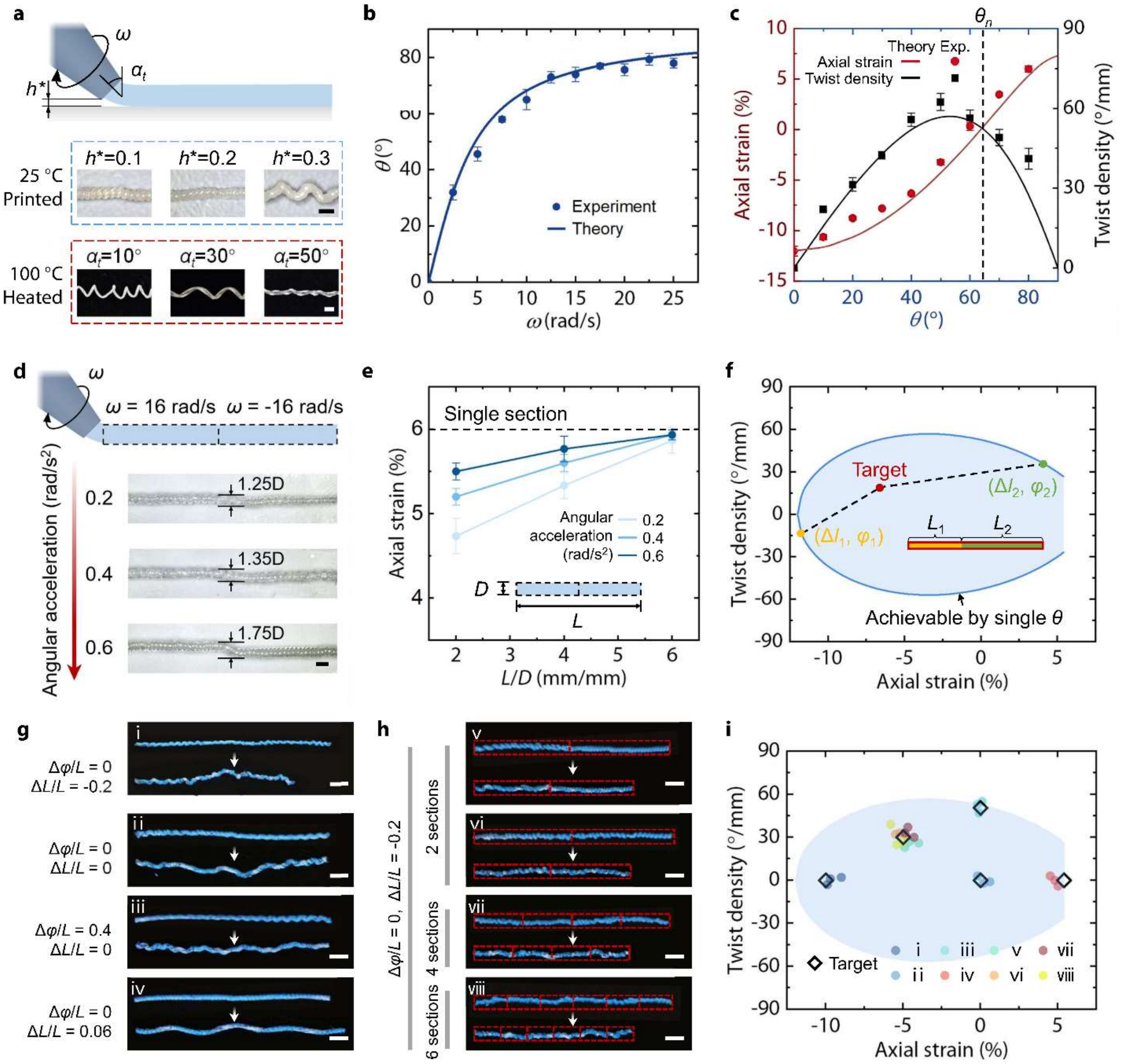

Figure 2. Single filament programming with multi-modality. (a) Comparison of filament morphologies printed at nozzle heights of h^*=0.1 mm, 0.2 mm and 0.3 mm, illustrating how overly low or high nozzle heights lead to excessive filament width or zigzag deposition. The bottom panels (100 °C) demonstrate the effect of nozzle tilt angle (α_t) on actuation symmetry, showing that $\alpha_t = 50^\circ$ yields stable helical deformation. (scale bar = 1 mm) (b) Schematic showing how the nozzle's angular speed ω corresponds to the filament helix angle θ, which can be continuously varied from -80° to $+80^\circ$. (c) Influence of different θ values on the LCE filament's twisting (right

axis) and axial deformation (left axis) upon heating, transitioning from pure axial contraction to primarily twisting, and eventually to axial elongation. (d) Effect of different angular accelerations on the stability of the printed filament shape, where higher accelerations cause noticeable distortion. (scale bar = 0.5 mm) (e) Axial deformation under different acceleration. (f) The shaded region represents all achievable combinations of axial and torsional deformations (programmable deformation design space), the blue boundary marks the achievable deformation under uniform θ. (g) Selection of target deformations (contraction, macroscopically invariant, twisting, elongation) within the design space by dividing the filament into segments with different θ. (scale bar = 2 mm) (h) Printed samples demonstrating various target shapes achieved via different number of segments. (i) Experimental results show such multiple segmentations programming strategy can precisely program both axial and torsional deformations (Scale bar=5 mm).

(ii) We thank the reviewer for their keen observation regarding the tensile data in Supplementary Fig. 14 (original Supplementary Fig. 9). This scrutiny helped us identify a critical unit conversion error in the raw data processing of our quasi-static tensile tests. The force data exported from the tester was in centinewtons (cN) but was inadvertently processed as Newtons (N), resulting in a 100-fold inflation of the reported stress and modulus values. We have re-processed the data with the correct units. The corrected mechanical properties are:

- Tensile Strength: Approximately 1.6 to 2.3 MPa.
- Young's Modulus: Approximately 0.5 – 1.1 MPa.

These corrected values are fully consistent with standard LCE literature and exhibit the expected soft elasticity. We have updated Supplementary Fig. 14 and the relevant text in the manuscript accordingly.
Crucially, we wish to clarify that this sensor unit error was isolated to the tensile characterization and does not affect the actuation performance reported in Supplementary Fig. 17:

1. Actuation Strain: This is a kinematic measurement derived from visual displacement, independent of force sensors.
2. Work Density: These values were obtained from isobaric experiments using calibrated dead weights. The stress was determined by gravity ($F=mg$) and is physically accurate, unaffected by the tensile tester's settings.

Thus, while the quasi-static stiffness has been corrected, the high actuation strain and work density of our system remain valid and robust. Furthermore, we emphasize that this absolute modulus unit error does not invalidate our analytical theoretical models in the SI. In our Neo-Hookean energy minimization (e.g., Eq. S20-S22), the absolute shear modulus μ mathematically cancels out when taking the derivatives $\partial E/\partial\lambda = 0$ and $\partial E/\partial\tau = 0$). Thus, our theoretical framework for predicting shape morphing completely relies on geometric parameters and remains rigorously robust.

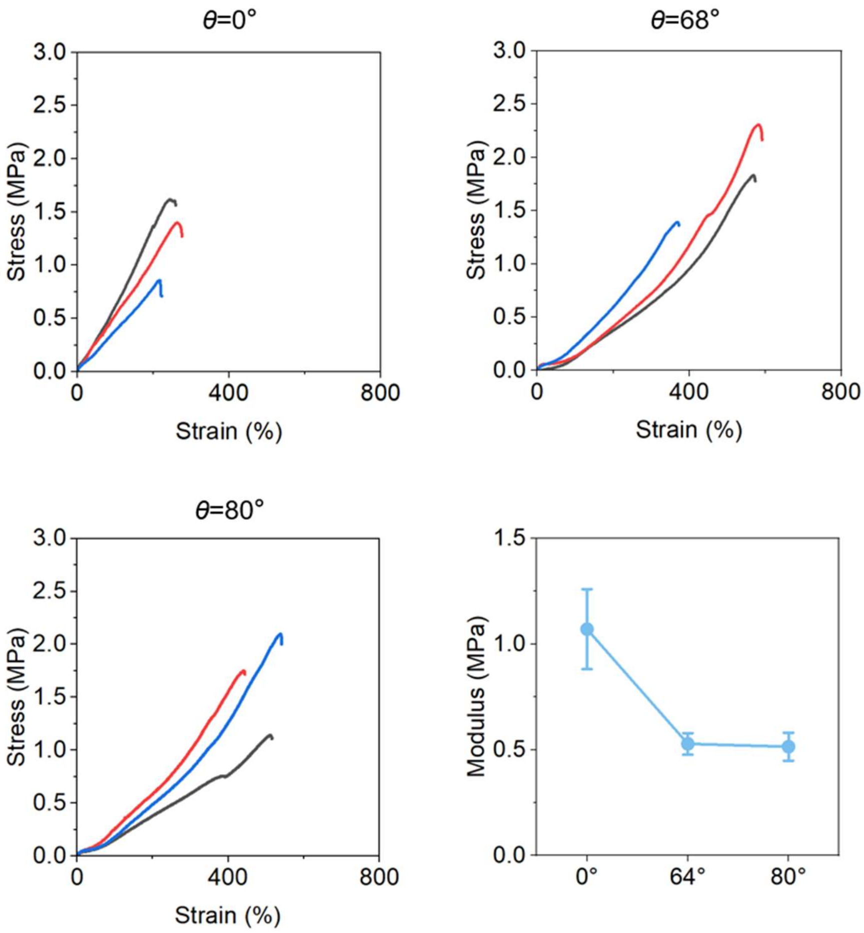

Supplementary Fig. 14. Tensile testing of LCE filaments with different rotational angles indicates that a higher rotational angle leads to a lower tensile modulus.

Comment #2: Significance

i) Interesting, but unlikely to be sufficiently impactful in the field of LCEs. Rather, this study demonstrates the impact fulness of rotational printing work from the Jennifer Lewis group. On-the fly modification of properties to spatially tune/program bulk deformation behavior is of great interest in 3D printing. However, this paper does not truly demonstrate 3D printing, as all structures are single-layered and have no variation in the vertical axis.

ii) Similar rotational DIW:

Larson, N.M., Mueller, J., Chortos, A. et al. Rotational multimaterial printing of filaments with subvoxel control. Nature 613, 682–688 (2023). https://doi.org/10.1038/s41586-022-05490-7

Original rotation printing:

J.R. Raney, B.G. Compton, J. Mueller, T.J. Ober, K. Shea, & J.A. Lewis, Rotational 3D printing of damage-tolerant composites with programmable mechanics, Proc. Natl. Acad. Sci. U.S.A. 115 (6) 1198-1203, https://doi.org/10.1073/pnas.1715157115 (2018).

iii) The supercoiling behavior introduced via the rotational deposition is a point of novelty, especially considering the massive improvements in actuation it offers. Regulated coiling behavior is also highlighted in the multi-stage gripper, which is a welcome novel addition to the ubiquitous "gripper" demonstration.

iv) If the tensile results presented in SI fig. 9 are accurate, then this should be a major focus of the paper, as this is unprecedented performance in LCE materials.

Response #2

i) Thank you for your suggestion on demonstrating 3D object. In the revised manuscript, we added Supplementary Fig. 16. showing the capability of gap-span printing at different rotational angle.

We also add a multilayer 3D lattice that explicitly leverage rotational printing to assign distinct local helix angles (θ) to different strut classes and/or layers, as indicated by the color-coded regions (new Fig. 3g; Supplementary Video 3). Under a single, spatially uniform heating condition, these programmed regions exhibit different deformation modes and amplitudes, which collectively drive a prescribed global 3D shape transformation. During 3D build-up, our platform employs in situ UV curing (~20 mW cm^{-2}) to rapidly crosslink the extruded ink and stabilize each deposited filament.

For cases where a strut must transition between θ values over a long span between two anchor points, we additionally print a small, removable sacrificial support at the switching location on the underlying layer to maintain geometric fidelity. This optional post-processing step does not affect the programmed actuation mechanism and is described in the Supporting Information.

In *Programming Helical Angles for Versatile Shape Transformations*, we revised:

"To demonstrate scalability beyond 1D/2D constructs, we printed free-standing multi-layer structures by out-of-plane stacking (e.g., a 6-layer pyramid, Fig. 3g) This 3D architecture leverages a pronounced structural stiffness gradient to guide localized morphing. The thick multi-layer core strongly resists bending, acting as a geometric anchor that funnels the θ-programmed actuation outward, thereby significantly amplifying the upward curling of the highly compliant single-layer perimeter. We also demonstrate that individual rotationally printed filaments can bridge millimeter-scale gaps, with sagging that increases at higher rotational angles (Supplementary Fig. 16, Supplementary Video 4)."

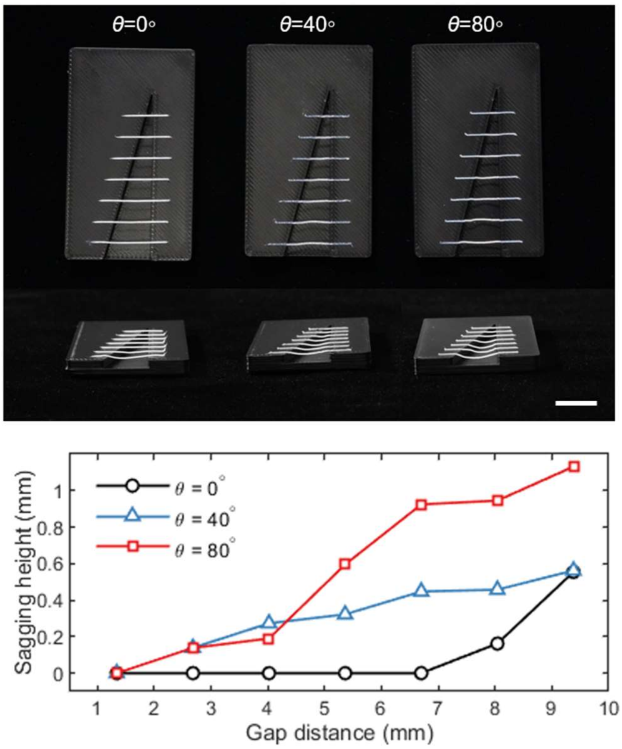

Supplementary Fig. 16. Gap-spanning performance of rotationally printed LCE filaments. Higher rotational angles result in larger sagging heights (scale bar, 5 mm).

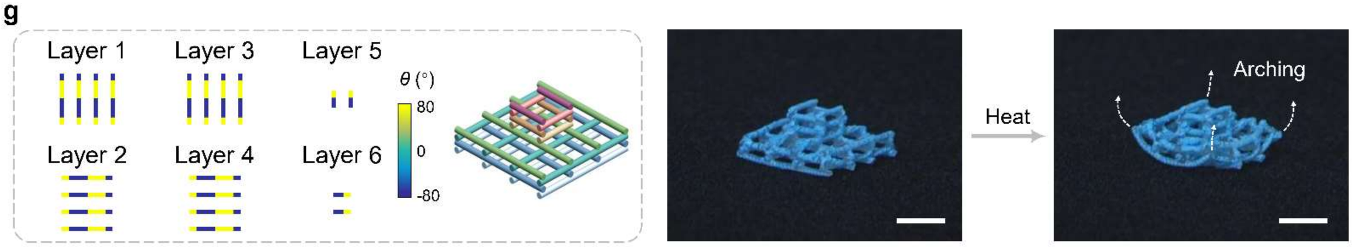

Figure 3. Helical angle programming of a LCE mesh structure yields multiple shape-morphing modes. (g) A 6-layer pyramid fabricated by out-of-plane stacking. Upon heating, the structural stiffness gradient firmly anchors the thick central core, forcing the thermal actuation to concentrate at the compliant perimeter and amplifying its upward curl. (Scale bar: 5 mm).

ii) We added a paragraph in Introduction and Supplementary Table 1 for comparison of our rotational printing of LCE with existing works.

In *Introduction*, we revised:

"Recently, rotational printing has been proposed for increasing mechanical performance and programming the sub-filament architectures. For example, Raney et al. demonstrated that rotating the nozzle during DIW could impart helical alignment to short fibers within a composite matrix, enhancing damage tolerance via crack steering[46]. Larson et al. further advanced this technique to rotational multimaterial printing, achieving sub-voxel control over filament microstructure to program anisotropic stiffness and swelling behaviors in hydrogels and epoxies[47]. Similarly, Ren et al. leveraged twisted interfaces in bi-material systems to generate mismatch strain for deformation or suture-like geometries for mechanical interlocking[48,49]. However, these strategies rely on structuring heterogeneous phases—either by aligning discontinuous fillers or manipulating material distribution. The potential of this kinematic strategy to program the intrinsic molecular director field within a monolithic filament for multimodal actuation remains underexplored. Leveraging nozzle rotation to decouple the actuation mode from the printing path in a single material could significantly expand the design space of soft structures and robotics, yet a systematic methodology for such single-parameter programming is still lacking (Comparison Table 1)."

Supplementary Table 1. Comparison of rotational 3D printing strategies for programming material microstructure and functional mechanisms.

Ref	Material	Programming target	Enabled microstructure	Functional Mechanism	Application
[5]	Fiber-reinforced Composites	Discontinuous Fibers (Micro-scale)	Helically aligned short fibers	Crack steering & deflection	Impact-resistant composites
[6]	Multi-material System	Material Composition (Meso/Voxel-scale)	Spatial helical heterogeneity	Structural anisotropy via modulus mismatch	Artificial muscles; Stiffness-graded lattices.
[7]	Bi-material Systems	Interface / Structure (Meso-scale)	Twisted bi-material interface	Inter-layer mismatch strain	Shape-shifting strips; Coiling actuators.
[8]	Bi-material Systems	Interface / Structure (Meso-scale)	Suture-like helical interface	Geometric mechanical interlocking	Mechanical metamaterials; Protective structures.
This work	Monolithic LCE	Intrinsic Director Field (Molecular-scale)	Helical molecular director field	Rotated intrinsic strain axes	Soft actuator; Adaptive Grippers; Medical Guidewires.

iii) Thank you for the positive assessment of our manuscript.

iv) We sincerely apologize for this oversight and thank the reviewer for their rigorous scrutiny, which allowed us to identify a unit export error (cN vs. N) from our static tensile tester. Importantly, we assure the reviewer that this sensor unit error was strictly isolated to the static tensile test. Our core actuation metrics (e.g., actuation strain, work density up to ~300 kJ/m³) were derived independently using visual kinematic tracking and calibrated physical dead-weights ($F=mg$), and therefore remain completely robust and unaffected.

Comment #3: Data and Methodology

i) The presentation of data and demonstrations in the manuscript are of very high quality.

ii) The methods are valid. Indeed, the influence of LCE fiber orientation on deformation of printed LCE structures is well explored. Moreover, spatial mechanical tunability via rotational DIW 3D printing has been successfully demonstrated and documented.

iii) The authors include extensive theoretical discussion of mechanical deformation in viscoelastic cylinders in SI. While this is a welcome justification, this reviewer questions the extent to which this theory was utilized in the design process.

iv) Spatially tunable material behavior is an important aspect of this paper. However, it does not provide sufficient justification of single-filament deformation versus angle (Figure 2g, h). The authors need to considerably strengthen their presentation of how twist and extension was documented including before and after images for angles used in measurements.

v) The reviewer appreciated the thought put into other demonstrations. Incorporating magnets as a secondary deformation mechanism is quite interesting but ultimately undermines the utility of the rotational printing technique as a viable standalone mechanism for imparting functionality.

vi) The combined phase transition and supercoiling approach to achieving beyond state-of-the-art work densities is impressive. However, it is not clear how they arrived at these results, as methodology is not discussed. It is also unclear what the filament is lifting in b, the authors should specify the weight.

Response #3

(i-ii) We thank reviewers for their comment.

(iii) We clarify that the theoretical model was strictly integral to our forward-design process rather than a post-hoc justification. Specifically, the analytical solutions (Eq. S21, Eq. S22 and S24) allowed us to mathematically predict the critical neutral helix angle (θ=68◦) for pure twisting with zero axial strain prior to experiments. Furthermore, our multi-segment inverse design strategy directly utilized the linear system derived from the compatibility conditions (Eq. S27-S29) as a quantitative blueprint to calculate the exact segment lengths (L_1, L_2) required to achieve arbitrary target macroscopic deformations (as shown in Fig 2), effectively eliminating blind trial-and-error.

In *Rotational printing enabled helix director field*, we added:

"Guided by our analytical energy-minimization model (Supplementary Section 3), we theoretically predicted and experimentally verified a critical neutral helix angle (θ_n = 68°) that exhibits primarily torsional twisting with minimal length change…"

In Programming single filament actuation, we added:

"For example, a two-segment filament with respective print angles θ_1 and θ_2 (and segment lengths L_1 and L_2) can be designed so that its overall twist and axial actuation match the target. Serving as an inverse-design tool to eliminate empirical trial-and-error, our theoretical framework directly dictates the required segment lengths L_1 and L_2 of each section determined by (Supplementary Section 4):"

(iv) We added a comprehensive photographic series of single filaments with varying helix angles θ before (25 ˚C) and after (100 ˚C) thermal actuation (Supplementary Fig. 11). These images visualize the distinct transition from axial contraction to pure twisting and elongation. Annotations are included to demonstrate how twist density and axial strain were measured. We also added before-actuation image in Figure 2g–h.

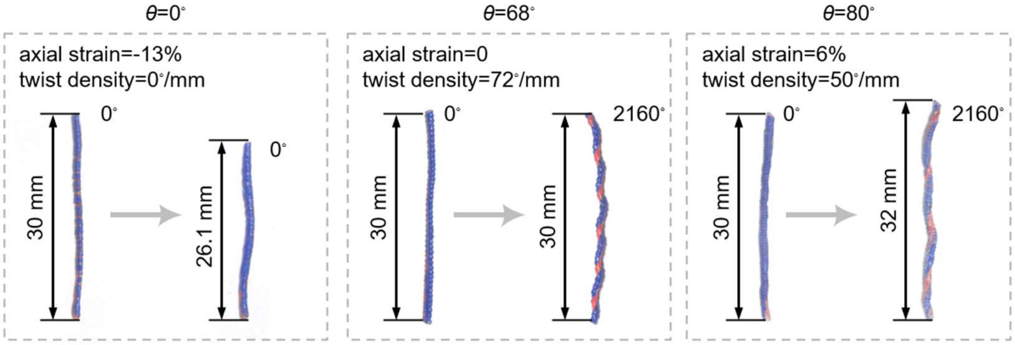

Supplementary Fig. 11. LCE filaments printed at θ = 0°, 68°, and 80° are shown before/after heating, revealing clear torsional rotation for $\theta \neq 0°$ with a θ = 0° non-twisting control. Twist density is quantified as a function of θ.

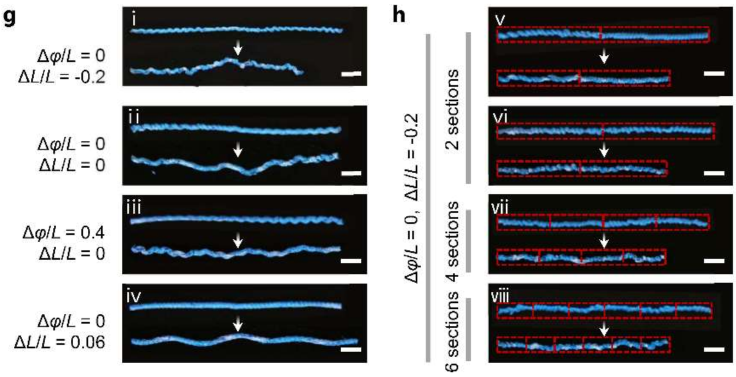

Figure 2. Single filament programming with multi-modality. (g) Selection of target deformations (contraction, zero deformation, twisting, elongation) within the design space by dividing the filament into segments with different θ. (scale bar = 2 mm)

(v) we clarify that the rotational printing technique acts as the foundational mechanism that determines the intrinsic actuation mode (contraction, twisting, or elongation). The introduction of magnetic composites is not to compensate for the printing technique, but solely to demonstrate the system's reprogrammability—enabling volatile and non-volatile memory effects that allow the fixed printed geometry to adaptively switch functions (e.g., the crawler's gait change).

<u>In *Programmable volatile and non-volatile configuration memory*, we added:</u>

"Here, we introduce magnetic composites not as a primary actuator, but to enable reprogrammable volatile and non-volatile memory on top of the printed helical director fields.

(vi) we apologize for the lack of clarity. We have added a dedicated section in the Methods detailing the calculation of work density derived from the combined phase transition and supercoiling. In addition, we have added Supplementary Fig. 18 summarizing the measured work density as a function of lifted load. This new figure provides a mapping from load mass to the reported work density values, and clarifies the operational range over which peak work density is achieved.

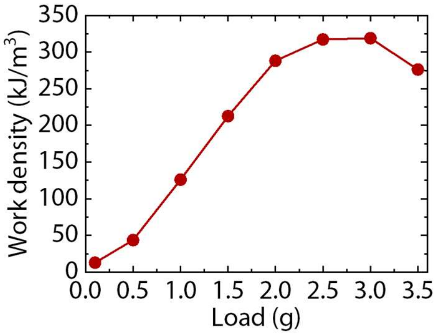

Supplementary Fig. 18. Volumetric work density as a function of lifted load.

<u>In *Method*, we added:</u>

"To evaluate the actuation performance and calculate the work density, LCE filaments with a programmed helix angle of θ were subjected to isotonic actuation tests. A filament sample with an initial length L_0 and diameter D was vertically suspended, with the top end fixed and the bottom end tethered to a load of mass m. The sample was then heated from ambient temperature (20 ◦C) to the actuation temperature (100 ◦C) using NIR lights. The

vertical displacement Δh of the load, resulting from the combined phase transition and supercoiling of the filament, was recorded using a high-resolution camera.
The mechanical work output (W) performed by the filament was calculated as the product of the gravitational force acting on the load and the displacement:

$$W = m \cdot g \cdot \Delta h$$

where g is the gravitational acceleration (9.8 m/s^2). The volumetric work density (U) was then derived by normalizing the work output by the initial volume of the active LCE filament V:

$$V = \pi(D/2)^2 \cdot L_0$$

$$U = \frac{W}{V}$$

This calculation assumes the deformation is uniform along the active length of the filament during the supercoiling process."

Comment #4: Analytical Approach
The demonstration of bulk "twisting" behavior is quite weak, only really apparent in multi filament structures.

Response #4
We appreciate the reviewer's comment. To strengthen the evidence for twisting of single filament and multi-filament assemblies, we added (i) Supplementary Fig. 11 of before/after optical images of individual filaments printed at $\theta = 0°$, 68°, and 80°, where torsional rotation is clearly visible for $\theta \neq 0°$ ($\theta = 0°$ serves as a non-twisting control), (ii) Supplementary Fig. 15 shows three mesh structures exhibiting distinct macroscopic twist angles after heating. Together, these additions show that twisting is an intrinsic, θ-programmed deformation mode and that it scales robustly to bulk architectures.

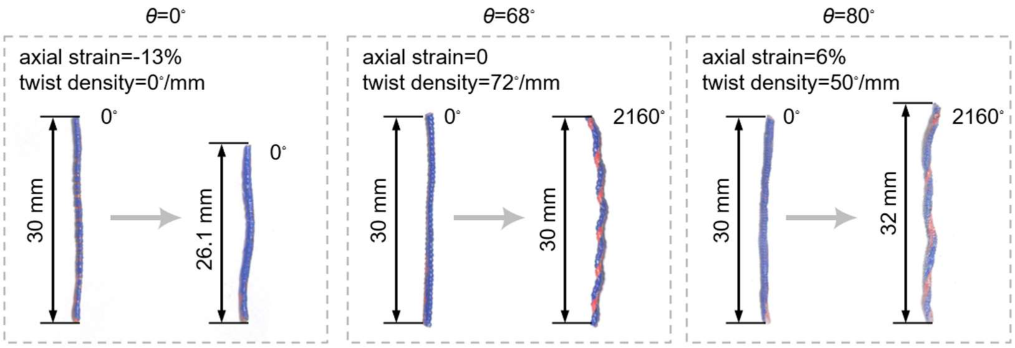

Supplementary Fig. 11. LCE filaments printed at $\theta = 0°$, 68°, and 80° are shown before/after heating, revealing clear torsional rotation for $\theta \neq 0°$ with a $\theta = 0°$ non-twisting control. Twist density is quantified as a function of θ.

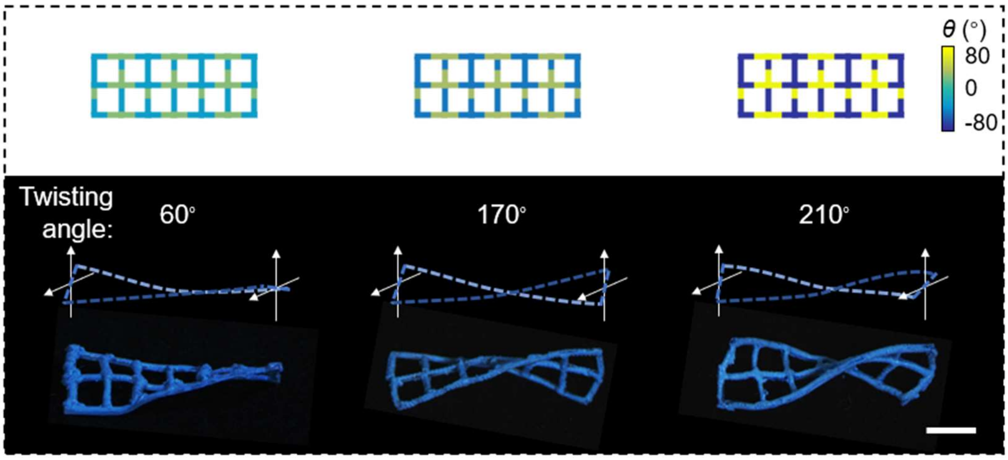

Supplementary Fig. 15. Three programmed mesh architectures exhibit distinct macroscopic twist angles after heating, demonstrating tunable bulk twisting via θ-programming. Scale bars and heating conditions are indicated.

Comment #5: Approach Clarity and Context

The results are clear, and comprehensible by a reader with little experience in LCEs. Ultimately it was an enjoyable paper to read. Unfortunately, the main focus of rotational deposition and controllable LCE deformation lacks novelty and the unbelievable and poorly supported materials characterization data are a major red flag.

Response #4

We strengthened single-filament twisting evidence by adding marker-based visualization and quantification of twist in isolated filaments (in addition to multi-filament assemblies), implemented in Fig. 2g–h and supporting SI panels; this demonstrates that twisting is not an emergent artifact only visible in bundles.

Comment #6: References

The author assembled and discussed prior work in LCE actuation thoroughly. However, the authors neglect to reference prior rotational printing work from J. Lewis indicating a surface understanding of the field and perhaps lacking some needed deeper perspective.

Response #5

We expanded the rotating-nozzle printing context and explicitly distinguished our mechanism from reinforcement- and multimaterial-interface driven rotational printing by adding citations and a comparison paragraph in Introduction and Discussion (Refs and additional LCE printing context refs); this provides the requested deeper perspective and prevents overstating novelty.

In *Reference*, we added:

46 Raney, J. R. et al. Rotational 3D printing of damage-tolerant composites with programmable mechanics. Proc Natl Acad Sci U S A 115, 1198-1203, doi:10.1073/pnas.1715157115 (2018).

47 Larson, N. M. et al. Rotational multimaterial printing of filaments with subvoxel control. Nature, 1-7 (2023).

48 Ren, L. et al. Rotational Co-extrusion 4D printing of heterogeneous filaments to enable sophisticated shape morphing. Additive Manufacturing 73, 103661 (2023).

49 Ren, L. et al. Rotational 3D printing spruce-inspired helical structural material with tunable mechanical properties. Chem Eng J 490, 151591 (2024).

Comment #7: Expertise
Detailed evaluation of the analytical discussion in the SI is outside of our expertise.

Comment #7: Expertise
We thank you for evaluating our manuscript.

Comment #8: Tensile analysis
The printed single filaments were directly used for mechanical tests. Tensile properties were measured on a fiber testing machine with a constant crosshead speed of 10 mm s^{-1}
We looked through the supplied tensile data; we could not replicate the Young's Moduli presented through calculations. After analyzing several other papers and speaking with another LCE expert (cited in paper) we believe that this data is flawed of the scale is misrepresented.

Response #8
We thank the reviewer for scrutinizing Supplementary Fig. 13, which revealed a unit conversion error (cN vs. N) causing a 100-fold inflation in tensile values. We have re-processed the data; corrected properties (Strength 1.6 to 2.3 MPa; Modulus ~0.5–1.1 MPa) align with literature and are updated in the manuscript. Crucially, this error is isolated to tensile characterization. Actuation performance (Supplementary Fig. 16) remains valid, as strain and work density were derived from visual displacement and calibrated weights, independent of the tensile sensor.

<u>Comment in Attached Files</u>

Comment #1
I would hesitate to call these structures "3D printed" considering they are single layer patterns. Extrusion is just a deposition technique rather than a means of creating substantial multi-layer structures.

Respond #1
We also add a multilayer 3D lattice (new Fig. 3g; Supplementary Video 3) and cross-gap experiment (Supplementary Fig. 16) to show the capability of 3D printing.

Comment #2
Questionable use of quiescent.
Respond #2
Thank you for your suggestion. We have replaced this term with macroscopically invariant.

Comment #3
Response → Respond

Respond #3
We have rephrased the whole sentence and removed the original word.

Comment #4
There is "pioneering" work from H. J. Qi and D. J. Roach at the same time. Include.

Respond #4

Thank you for your important suggestion, we have now added ref [31-32] to highlight their contribution in this field.

<u>In *Reference*, we revised:</u>

"31 Roach, D. J., Kuang, X., Yuan, C., Chen, K. & Qi, H. J. Novel ink for ambient condition printing of liquid crystal elastomers for 4D printing. *Smart Materials and Structures* **27**, 125011 (2018).

32 Ambulo, C. P. *et al.* Four-dimensional Printing of Liquid Crystal Elastomers. *ACS Appl Mater Interfaces* **9**, 37332-37339 (2017)."

Comment #5

this is what you did.

Respond #5

Thank you for your comment, we have revised the Introduction to make a clearer expression of the contribution of our work.

<u>In *Introduction*, we revised:</u>

"This constraint largely fixes the filament-level programmed eigenstrain to a dominant axial response along the deposition direction, such that the intrinsic actuation palette of a monolithic extruded filament remains effectively one-dimensional; while sophisticated shape changes (e.g., planar folding[8,30], arching[38,39], and twisting[37,40]) and even richer, non-developable curvatures (including Gaussian curvature) can be achieved via antagonistic/interface designs, voxelation/multi-material architectures, and/or spatially patterned stimuli, these approaches typically add geometric/toolpath or stimulus-field complexity. As a result, the printed LCE structures exhibit strong coupling between deformation mode and printing path, complicating independent control of distinct deformation dimensions."

Comment #6

Acknowledge Lewis group rotational printing papers from 2017 and 2023. Most recent one has an identical printing apparatus and technique. Draws the same conclusions. This is the same thing with LCEs. Not sufficiently novel.

Respond #6

We have cited these papers, including Supplementary Table 1 to show the comparison of our work with existing rotational printing.

Comment #7

Imprinting→ Encoding

Respond #7

We have replaced imprinting with encoding.

Comment #8

how to ensure filament diameter is consistent? This will influence deformation as well.

Respond #8

we added a quantitative calibration of deposited strand width/diameter relative to nozzle diameter (D_f/D_n) across the investigated print speed (v) and rotation speed (ω) space (Supplementary Fig. 9), and explicitly state how D_f is measured and how this informs the effective filament-level scale used in the manuscript.

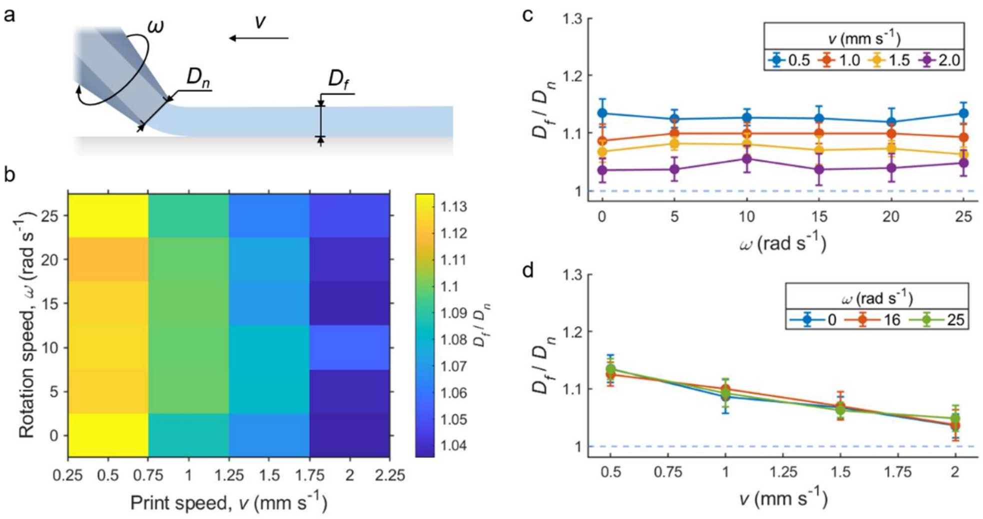

Supplementary Fig. 9. Deposited strand geometry calibration across translation and rotation speeds**.** (a) Definition of nozzle diameter D_n and deposited strand diameter D_f. (b) Map of D_f/D_n across print speed v and rotation speed ω. (c–d) Representative line cuts showing D_f/D_n at different ω and vs. v.

Comment #9

2-axis robotic arm ⟵ I don't think you can call it that.

Respond #9

Thank you for your suggestion, we have replaced this term with two-axis translation stage.

Comment #10

Conflicting terminologies between fields. Rephrase or remove "DNA"

Respond #10

Thank you for your suggestion, we apologize for the inappropriate terminology. We have removed "DNA-inspired" throughout the manuscript.

Comment #11

Conflicting terminologies between fields. Rephrase or remove "DNA"

Respond #11

Thank you for your suggestion, we apologize for the inappropriate terminology. We have removed "DNA-inspired" throughout the manuscript.

Comment #12

Spreading ⟵ over-extrusion or stress relaxation of LCE resin?

Respond #12
Thank you for your suggestion, we have replaced the phrase with over-extrusion.

Comment #13
Often yielding an asymmetric filament cross-section ← example?

Respond #13
Thank you for your suggestion, we have added Supplementary Fig. 10 to show the filament printed under different tilt angle.

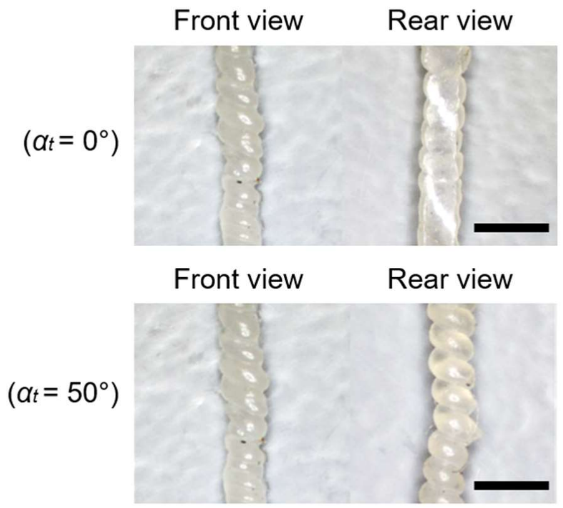

Supplementary Fig. 10. Filament printed at different tilt angle (Scale bar 1 mm).

Comment #14
Neutral angle ← what does this mean?

Respond #14
We agree that this term needs a clear definition. In our context, the "neutral angle" refers to the specific, critical programmed helix angle at which the thermal actuation results in pure twisting, with exactly zero net macroscopic axial extension or contraction.

<u>In *Programming single filament actuation*, we revised:</u>
"At a neutral angle θ_n of approximately 68°, the LCE filament exhibits pure macroscopic twisting upon heating, with zero net axial strain, indicating an almost pure torsional actuation."

Comment #15
Dependent ← Dependence.

Respond #15
Thank you very much, we've corrected the expression.

Comment #16
Supplementary Fig. 7 ← Curve labels?

Respond #16
We've add labels to Supplementary Fig. 12 (previous Supplementary Fig. 7).

Comment #17

Apart from thermal-induced deformation, adjusting θ can also program the mechanical property of printed filament for various applications ← Explicitly state tensile properties and what the trend was. Otherwise this is pointless.

Respond #17

We agree that the original statement was too vague. We have revised the manuscript to specify the tensile properties evaluated and clearly describe their exact trends in relation to the angle θ.

In *Programming single filament actuation*, we revised:

"Apart from thermal-induced deformation, adjusting θ can also program the mechanical property of printed filament. Specifically, as θ increases, the Young's modulus gradually decreases from ~1.1 MPa $\theta = 0°$ to ~0.5 MPa at $\theta = 80°$ (Supplementary Fig. 14)."

Comment #18

Apart from thermal-induced deformation, adjusting θ can also program the mechanical property of printed filament for various applications. ← Explicitly state tensile properties and what the trend was. Otherwise this is pointless.

Respond #18

We agree that the original statement was too vague. We have revised the manuscript to specify the tensile properties evaluated and clearly describe their exact trends in relation to the angle θ.

In *Programming single filament actuation*, we revised:

"Apart from thermal-induced deformation, adjusting θ can also program the mechanical property of printed filament. Specifically, as θ increases, the Young's modulus gradually decreases from ~1.1 MPa $\theta = 0°$ to ~0.5 MPa at $\theta = 80°$ (Supplementary Fig. 14)."

Comment #19

We examined how to change the nozzle's rotation during the printing process. ← How to do it should be obvious, rephrase

Respond #19

Thank you for your suggestion. We agree and have rephrased the sentence for clarity.

In *Programming single filament actuation*, we revised:

"To program spatial variations of θ along a single filament—and thereby achieve more complex, decoupled deformation modes— we dynamically modulated the nozzle's rotation during the printing process."

Comment #20

Eq. (2) ← Explain terms, L vs. l not totally clear

Respond #20

We have changed l to Δl to better clarify the meaning of the term. We have revised the subsequent paragraph to explain terms of Eq. (2).

In *Programming single filament actuation*, we revised:

"Where Δl_{t} is target axial strain, φ_{t} is target twisting density. Δl_1 and Δl_2 are axial strain at θ_1 and θ_2, respectively, φ_1 and φ_2 are twisting density at θ_1 and θ_2, respectively."

Comment #21

Designation of "twist" in these structures is unclear to the point of erroneous. The filaments shown exhibit a great deal of local twisting, iii does not appear to "twist" much at all compared with ii. Indeed, the "zero length deformation" in ii is spurious considering the outward deformation.

Respond #21

We thank the reviewer for the insightful comment. This observation highlights the need to distinguish local deformation from macroscopic end-to-end actuation. In our study, "twist" and "length deformation" strictly denote the global net torsional rotation ($\Delta\varphi/L$) and net axial strain (($\Delta L/L$) across the entire filament.

Structure (ii) comprises sequential segments with opposing programmed angles. Upon heating, these segments twist in opposite directions. This torque mismatch causes localized distortion (the observed "local twisting"). However, these opposing actuations perfectly cancel out globally, yielding strict net-zero end-to-end rotation and length change.

Conversely, structure (iii) is programmed for unidirectional macroscopic torsion. Although it appears less locally distorted than (ii), its ends exhibit a large net global rotation. We have clarified these definitions in the revised manuscript.

In *Programming single filament actuation*, we added:

"It is worth noting that the "twist" and "length deformation" discussed here refer to the macroscopic end-to-end net rotation and axial strain. For instance, in mode (ii), adjacent segments with opposing programmed angles twist in opposite directions upon heating, but they cancel out globally, resulting in a macroscopically invariant shape."

Comment #22

I have doubts this mathematical methodology was actually used in the design of any structure. Unsure why it is included.

Respond #22

We wish to clarify that our theoretical model was a fundamental component of our forward-design process, not a post-hoc rationalization. Prior to any experiments, the analytical solutions (Eq. S21, S22, and S24) enabled us to predict the critical neutral helix angle (θ=68°) required for pure twisting with zero axial strain. Additionally, our multi-segment inverse design strategy relied directly on the linear system derived from the compatibility conditions (Eq. S27–S29). This provided a quantitative blueprint to calculate the precise segment lengths (L_1, L_2) needed to realize arbitrary target macroscopic deformations (Fig. 2), thereby eliminating the need for blind trial-and-error.

Comment #23

The green and yellow markers. ← Incorrect color?

Respond #23

Sorry for the mistake, we have corrected it to blue and white.

Comment #24

Combining two distinct θ values ← What are they?

Respond #24

We thank the reviewer for pointing out this ambiguity. In this context, the two distinct θ values refer to the specific, programmed helix angles (θ_1 and θ_2) assigned to the two individual segments of the LCE filament to achieve the combined deformation.

In *Programming single filament actuation*, we revised:

"The blue and white markers in the figure indicate the programmed orientations of the two segments for each case, demonstrating how combining two distinct programmed helix angles (θ_1 and θ_2 for the first and second segments, respectively) yields the overall targeted deformation."

Comment #25

Figure 2(a) ← Explain the heating part of the figure.

Respond #25

We've marked heated part in Figure 2(a).

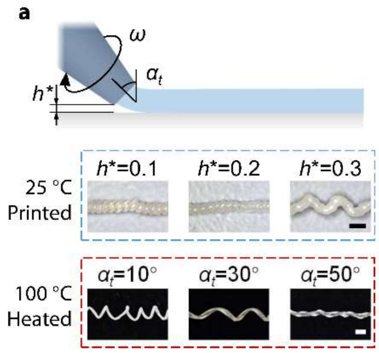

Figure 2. Single filament programming with multi-modality. (a) Comparison of filament morphologies printed at nozzle heights of h^*=0.1 mm, 0.2 mm and 0.3 mm, illustrating how overly low or high nozzle heights lead to excessive filament width or zigzag deposition. The bottom panels (100 °C) demonstrate the effect of nozzle tilt angle (α_t) on actuation symmetry, showing that α_t = 50° yields stable helical deformation. (scale bar = 1 mm)

Comment #26

Figure 2(c) ← Consider color coding each axis

Respond #26

Thank you for your suggestion, we have color coded both axis of the figure.

Comment #27

Figure 2(d) ← Improve resolution

Respond #27

Thank you for your suggestion, we have improved the resolution of this figure.

Comment #28

Figure 2(g) ← Need a pre-deformation image. Ideally a video, otherwise I am having a hard time believing twisting vs contraction claims. Indicate where differing sections are.

Respond #28

Thank you for your suggestion. First, we've added pre-deformation image of the filament. Also, we revised figure to identify different sections.

Comment #29
(scale bar = 2 mm) ← where?

Respond #29
We've added scale bar for all optical images.

Comment #30
We build on a previously established helical-angle programming strategy for one-dimensional LCE filaments and extend it to two-dimensional LCE mesh structures, enabling complex shape morphing. ← Do you mean the previous section or an existing publication?

Respond #30
We apologize for the inappropriate expression. We've revised the sentence to clarify that this method is based on previous section.

In *Programming single filament actuation*, we revised:
"Building on the helical-angle programming strategy for one-dimensional LCE filaments, we extend it to two-dimensional LCE mesh structures, enabling 2D to 3D shape morphing."

Comment #31
We build on a previously established helical-angle programming strategy for one-dimensional LCE filaments and extend it to two-dimensional LCE mesh structures, enabling complex shape morphing. ← Not terribly complex. Control of deformation or strength with "1D" material modalities via deposition change is very well explored and understood.

Respond #31
Thank you for your suggestion. We've rephrase it to "we extend it to two-dimensional LCE mesh structures, enabling 2D to 3D shape morphing."

Comment #32
With a low nematic–isotropic phase transition temperature (~18 °C) ← Same material as in figure S11?

Respond #32
Yes, Figure S11 (current Supplementary Fig. 19) shows the result using LCE with T_{NI} of ~18 °C. We've revised the figure caption to clarify this.

Revised figure caption:
"**Supplementary Fig. 19.** Sequential actuation of supercoiling LCE (T_{NI}=18 °C) ring with different rotational angle. (scale bar = 5 mm)"

Comment #33
which rapidly cross-links the extruded LCE ink to lock in helical alignment. ← Wouldn't crosslinking hinder actuation?

Respond #33

The actuation takes place after the whole printing process. The crosslinking density is engineered to be high enough to "lock in" the programmed nematic director field and prevent structural relaxation, yet low enough to maintain a lightly crosslinked elastomer network. This low crosslink density preserves significant free volume and chain mobility, allowing the mesogens to freely undergo the entropy-driven nematic-to-isotropic phase transition upon heating.

Comment #34

Supplementary Fig. 3. The oligomeric LCE ink is obtained after a prepolymerization step, and subsequent UV crosslinking during the printing process fully cures the material. ← UV intensity?

Respond #34

Thank you for your suggestion, we've added the UV intensity in the figure caption

Revised figure caption:

"**Supplementary Fig. 3.** The oligomeric LCE ink is obtained after a prepolymerization step, and subsequent UV crosslinking (20 mW cm^{-2}) during the printing process fully cures the material."

Reviewer #3

General Comment

This manuscript describes extrusion of liquid crystal elastomers using a rotating nozzle to control actuation properties. The filament and mesh structures that are produced are interesting, and the approach seems novel. However, the impact is lessened by the limited types of structure produced, and some of the writing/descriptions are not clear. I would recommend one major additional demonstration of the novel approach, substantial rewriting for clarity, and addressing of the following points.

Comment #1

The main major demonstration that seems necessary for publication is the demonstration of an actual 3D printed object that uses the highlighted technique of rotational printing to control local actuation. The authors include "3D-printed" in their title and only describe their 1D filaments and 2D mesh structures. 3D printing clearly offers substantial impact, and it would be an important consideration whether this technique can actually be used for 3D printing and what the limitations are. For example, can the technique enable the printing of tall structures? Can the technique be used to span gaps in a 3D lattice-type structure or similar?

Response #1

We appreciate the reviewer's important point regarding the need to demonstrate a truly three-dimensional printed object with spatially programmed actuation. In the revised manuscript, we added Supplementary Fig. 16. showing the capability of gap-span printing at different rotational angle.

We also add a multilayer 3D lattice that explicitly leverage rotational printing to assign distinct local helix angles (θ) to different strut classes and/or layers, as indicated by the color-coded regions (new Fig. 3g; Supplementary Video 3). Under a single, spatially uniform heating condition, these programmed regions exhibit different deformation modes and amplitudes, which collectively drive a prescribed global 3D shape transformation. During 3D build-up, our platform employs in situ UV curing (~20 mW cm^{-2}) to rapidly crosslink the extruded ink and stabilize each deposited filament.

For cases where a strut must transition between θ values over a long span between two anchor points, we additionally print a small, removable sacrificial support at the switching location on the underlying layer to maintain geometric fidelity. This optional post-processing step does not affect the programmed actuation mechanism and is described in the Supporting Information.

In *Programming Helical Angles for Versatile Shape Transformations*, we revised:

"To demonstrate scalability beyond 1D/2D constructs, we printed free-standing multi-layer structures by out-of-plane stacking (e.g., a 6-layer pyramid, Fig. 3g) This 3D architecture leverages a pronounced structural stiffness gradient to guide localized morphing. The thick multi-layer core strongly resists bending, acting as a geometric anchor that funnels the θ-programmed actuation outward, thereby significantly amplifying the upward curling of the highly compliant single-layer perimeter. We also demonstrate that individual rotationally printed filaments can bridge millimeter-scale gaps, with sagging that increases at higher rotational angles (Supplementary Fig. 16, Supplementary Video 4)."

In *Method*, we added:

"Free-standing multilayer structures (Fig. 3g) and gap-spanning struts (Supplementary Fig. 16) were fabricated

by extending the rotational-printing workflow to layer-by-layer build-up. Sliced toolpaths were post-processed to embed nozzle-rotation commands, enabling spatially prescribed helix angles (θ) across different strut classes and/or layers within a single print. During deposition, each filament was stabilized via in situ UV curing (irradiance ≈ 20 mW cm^{-2}) to rapidly crosslink the ink and maintain geometric fidelity during stacking. For gap spanning, struts were printed between two anchor points across millimeter-scale openings and the resulting sagging was quantified as a function of θ (Supplementary Fig. 16). When a long-span strut required a discrete θ transition, a small removable sacrificial support was optionally printed at the switching location on the underlying layer and removed after curing; this auxiliary step does not affect the programmed actuation mechanism."

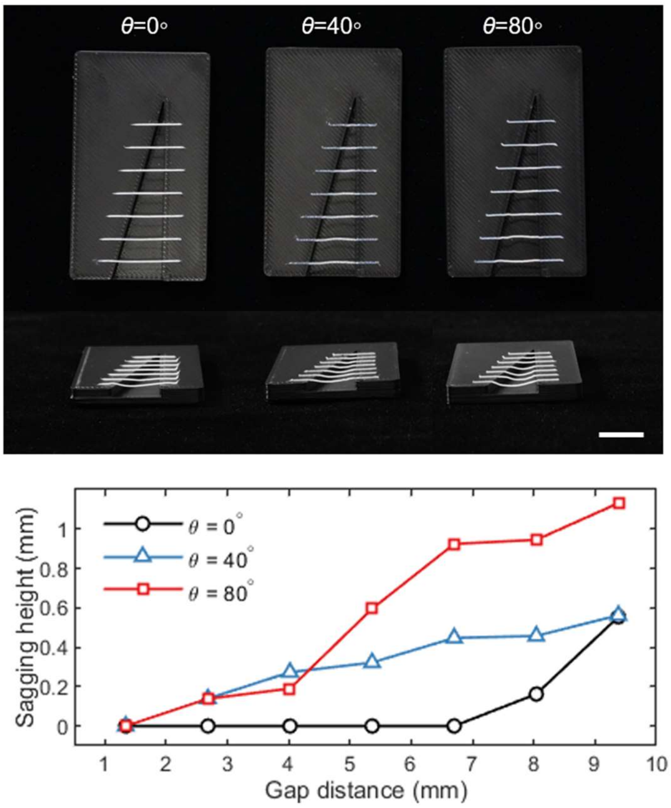

Supplementary Fig. 16. Gap-spanning performance of rotationally printed LCE filaments. Higher rotational angles result in larger sagging heights (scale bar, 5 mm).

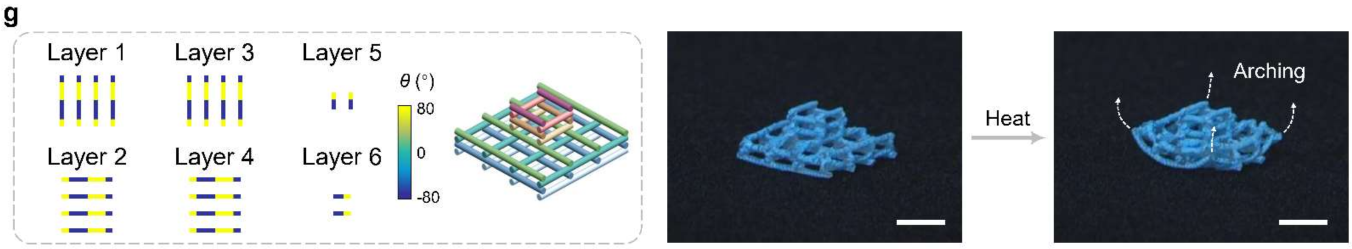

Figure 3. Helical angle programming of a LCE mesh structure yields multiple shape-morphing modes. (g) A 6-layer pyramid fabricated by out-of-plane stacking. Upon heating, the structural stiffness gradient firmly anchors the thick central core, forcing the thermal actuation to concentrate at the compliant perimeter and amplifying its upward curl. (Scale bar: 5 mm).

Comment #2

i) There is a quite a bit of jargon that lacks substance, inexplicit writing, and lack of clarity in some of the main text, captions, and figures. I apologize if I misunderstand or misread some of the writing; there were many

examples that I had trouble parsing, so it is possible that I overlooked detail that could have been already clarified. If that occurred, it still represents a problem with the manuscript's readability since it was difficult to follow in many instances. Some examples are laid out here. First, physical intelligence is not defined in the abstract; the definition in the introduction is clear, but as a reader, the phrase, "LCEs are promising in physical intelligence" seems nonsensical (especially with other fields like AI adopting and utilizing the phrase physical intelligence, possibly more than functional matter researchers are using it.) The phrase "selective heating of LCE segments with magnetic composites make shape programs rewritable" is not clear. It sounds like the authors are saying that they are using magnetic composites to heat the LCE, and it also sounds like the LCE is what is being reprogrammed, but it seems only the magnetic orientation is changing allowing for different response in a fixed field. what that means. Other phrases that aren't really clear include "using their intrinsic material properties and morphology" and "impart a form of embodied logic". Additionally, it seems inaccurate to denote zero net change as a "deformation mode". There was also a typo in the first part of the introduction, "ability to autonomously response", and there may be more that I missed.
ii) Within the figures and figure captions, the following issues stood out to me. Figure 1c shows the constituents of the LCE ink but shows nothing about the "formation" of the ink as suggested by the caption. Figure 1f is not clear what the axes mean, and the labels i through iv are not explained well. In Figure 2a, the images labeled as 100 C are not clear; the text for these mentions filament cross section being asymmetric but why is it shown at 100 C? It also shows a top view of the filament it appears, not the cross section. The caption only mentions the top set of images (modifying h*). In Figure 2e, there appears to be a legend missing. In Figure 2g and 2h, I think these are supposed to be filaments shown at elevated temperatures, but the temperature is not mentioned. The original shape is not shown for comparison.

Response #2
i) We apologize for the lack of clarity and the jargon.
1. We have removed the "physical intelligence" phrasing as noted in Response #2.
2. We rewrote the section on magnetic reprogramming to clearly state that the magnetic orientation is re-aligned by heating the composite above its transition temperature in a magnetic field, locking in a new magnetic easy axis upon cooling.
3. We have renamed "zero net change" to " macroscopically invariant" to accurately reflect that the material is active but the forces balance to maintain dimension.
4. We have proofread the manuscript to correct typos and other grammatical errors. We have extensive rewriting of the Introduction and Abstract for clarity; removal of jargon; correction of typos.

<u>In *Abstract*, we revised:</u>
"Embedding complex actuation capabilities into monolithic soft materials remains a challenge for the advancement of soft robotics. Liquid crystal elastomers (LCEs) offer intrinsic stimuli-responsiveness, yet conventional extrusion-based 3D printing restricts them to uniaxial alignment at filament level, limiting their utility to simple contraction modes. Here we introduce a rotational 3D printing approach that embeds a helical director field within LCE filaments, enabling multimodal actuation controlled by a single fabrication parameter: the helix angle (θ). Tuning θ programs each filament to contract, elongate, twist or remain macroscopically invariant when heated, decoupling actuation logic from device geometry. Spatial gradients in θ create a hierarchy of activation temperatures, yielding sequential shape changes under uniform heating. Localized heating of the magnetic-LCE composite segments allows their magnetic domains to be reoriented, making the shape programs rewritable and enabling switchable volatile and non-volatile memory. We demonstrate these capabilities in self-partitioning grippers, multimodal/color robots and reprogrammable guidewires that perform multi-step or

adaptive tasks without external circuitry. By encoding actuation modes, deformation sequences, and memory in a single parameter, this approach establishes a paradigm of material-encoded programmability and points toward monolithic soft robots and reconfigurable structures."

In *Introduction*, we revised:

"Stimuli-responsive materials provide a pathway to soft machines capable of complex, untethered shape transformations. By leveraging intrinsic thermomechanical properties rather than external motors, these systems can achieve sophisticated behaviors such as self-folding, locomotion, and adaptive gripping[1-5]. Rather than relying solely on external computation or rigid control algorithms, stimuli-responsive systems leverage material architectures to realize behaviors such as self-shaping[6-10], self-sensing[11-13], and adaptive response[14-16] in situ. A key challenge in this paradigm is creating actuators that can exhibit desired modes of behavior, enabling machines with multi-modal, adaptive and even reconfigurable response[2,3,17]. Recent advances in smart materials have started to address this: for example, liquid crystal elastomers (LCEs) can be programmed to undergo large, predetermined shape changes (e.g. contraction, bending, or twisting) when stimulated by heat or light[18-20]. By encoding molecular alignment within an LCE, researchers can impart a form of encoded anisotropy—the material itself directs how it will move under stimuli, contributing to the system's overall functionality[6-9,14,15]. However, traditional programming methods are typically tailored for simple deformation mode (e.g., mechanical stretching[21-23]), limited to thin-film LCEs (e.g., surface patterning[24-26]), or limited complexity and materials (electrical/magnetic field alignment[19,27,28]). Creating devices that can achieve complex actuation modes usually requires combining separate actuators or complex architectures, adding design and fabrication complexity that run counter to the concept of monolithic, integrated design.

3D printing has emerged as a powerful approach … Ideally, a single monolithic LCE element that can be programmed with any desired deformation mode in situ would offer greater adaptability, simplifying the creation of responsive machines with rich behavior repertoires."

We revised the section title *DNA inspired evolution and mutation* to *Programmable volatile and non-volatile configuration memory*. In this section, we revised:

"To realize versatile and reconfigurable soft robotics, materials are required to exhibit tunable memory capabilities, allowing for both reversible shape shifting and reprogramming of their actuation behaviors. Here, we introduce magnetic composites not as a primary actuator, but to enable reprogrammable volatile and non-volatile memory on top of the printed helical director fields. The composite structure comprises 10 discrete LCE segments arranged in series, each functioning as a tri-section composite unit. The two terminal ends of each segment are LCE components fabricated via multi-step rotational printing (Supplementary Fig. 23), imparted with specific helix angles θ so that they undergo predetermined multi-angled twisting deformations upon thermal activation (Supplementary Fig. 24a). The central portion of each segment is a magnetized NdFeB–LCE composite. When heat is applied, the end LCE components twist, causing the central magnetic section to rotate and thereby reorient its magnetic domains (Figure 5a, Supplementary Fig. 25).

By tailoring the helix angles of the terminal LCE segments based on the twist–deformation profile from the printing process, the rotation angle of each central magnetic segment can be precisely controlled, effectively programming the leaf's magnetic response behavior. The LCE composite also exhibits dynamic, temperature-dependent configuration changes. An increase in external temperature induces a coordinated global rotation of all segments, which is reversed when the temperature decreases. This reversible behavior indicates a volatile memory effect, as the thermally induced configuration change "vanishes" upon cooling (Figure 5b). As shown in Figure 5c and Supplementary Fig. 26a and Supplementary Video 8, gradually raising the ambient temperature from 20 °C to 100 °C causes the LCE segments to progressively reorient, resulting in a spectrum of different deformed shapes

when the structure is subsequently exposed to a magnetic field. This volatile reconfiguration capability facilitates adaptive robotic functions. Drawing on the mimic octopus (*Thaumoctopus mimicus*), which simultaneously alters body shape and color[57,58], we built a four-segment crawler with different colors on each side (Figure 5d, Supplementary Fig. 24b). At 20 °C the robot walks with its white surface uppermost; upon entering an 80 °C zone the heated segment twists, flipping to its blue side and altering both magnetization and gait. Successive flips progressively convert a white, walking robot into a blue, crawling one, demonstrating multi-modal adaptation encoded entirely in the material (Figure 5e, Supplementary Fig. 26b, Supplementary Video 9).

Conversely, a sudden localized thermal stimulus can induce a non-volatile change in the LCE leaf's configuration that persists even after the stimulus is removed. In other words, a rapid, concentrated heating of a specific region produces a locked reorientation in those LCE segments, and this altered state remains "memorized" in the leaf's magnetic response after cooling, exemplifying a non-volatile memory effect (Figure 5f). The sequence illustrated in Figure 5g and Supplementary Video 10 demonstrates these principles in detail. Initially, without any induced reprogramming, the structure remains consistent and stable as the strength of an applied magnetic field is increased. Next, under a moderate magnetic field of 5 mT, segments 9 and 10 are rapidly heated with a heat gun, inducing local deformations that produce an approximately 180° rotation in their magnetic domain orientation. When the magnetic field is subsequently increased to 40 mT, the tip of the structure bends downward as a result of the earlier thermal reprogramming. Finally, further localized heating of the front and middle segments causes the structure to deform into an "N"-shaped configuration under the magnetic field (Supplementary Fig. 27a). Throughout these adaptive transformations, the LCE structure demonstrates an ability to adjust its torsional configuration and magnetic orientation in response to changing thermal and magnetic conditions."

Revised Figure 5

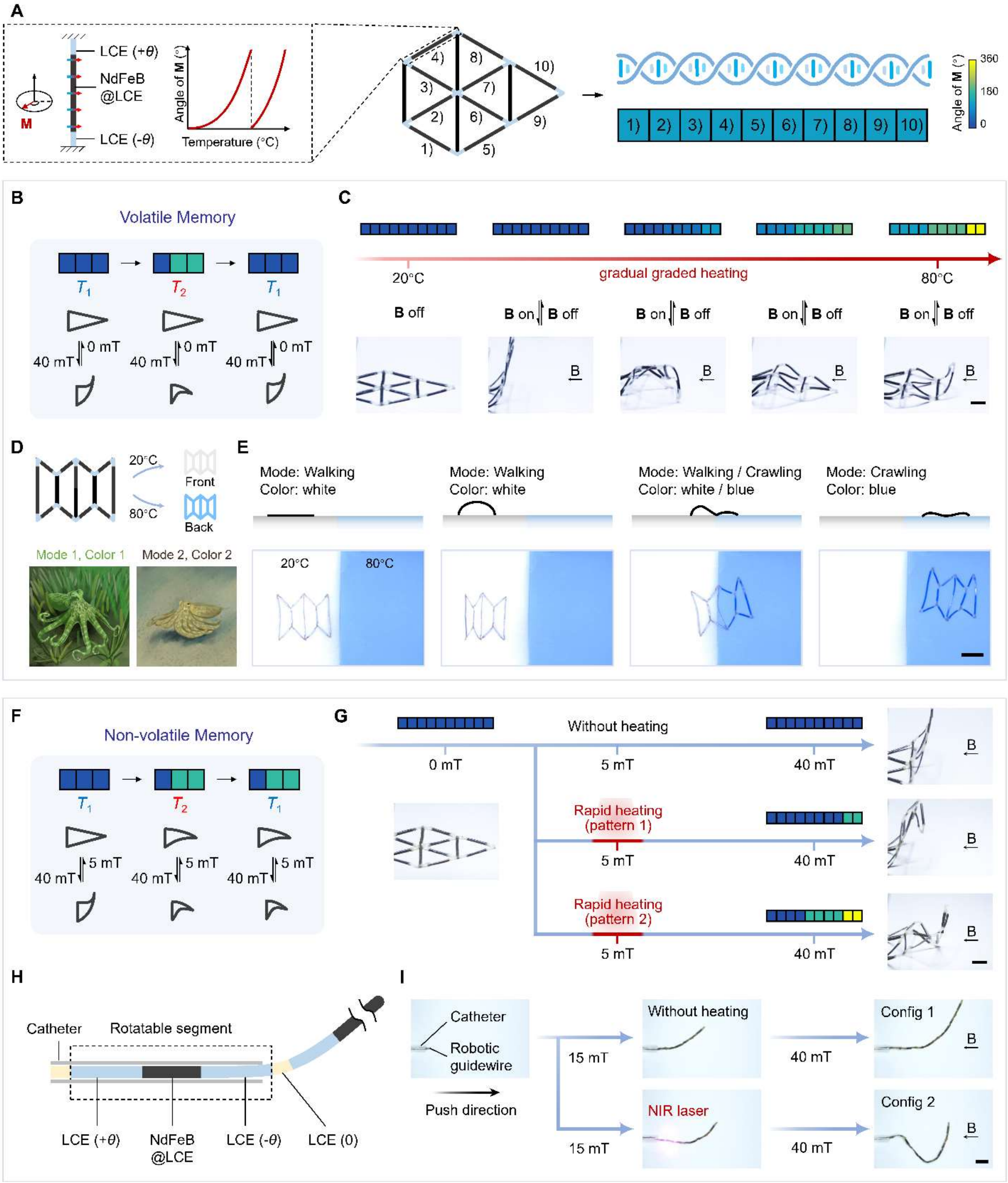

Figure 5. Magnetic-LCE structures with programmable volatile and non-volatile memory. (a) Each LCE leaf is composed of 10 discrete LCE segments, each containing two types of LCE sub-structures with opposite twist and a magnetic composite substructure, resulting in rotation of magnetization under thermal actuation. (b) Increasing the external temperature induces a coordinated global rotation in the LCE leaf (collective twisting of all segments), which disappears upon cooling—demonstrating volatile memory. (c) This temperature-driven reconfiguration leads to varied deformation profiles when the leaf is subjected to a magnetic field. (scale bar=5 mm) (d) Four segment LCE crawler demonstrating color and gait switching enabled by volatile reconfiguration. (e) Robot segments flip from white to blue as they traverse an 80 °C zone, transforming a walking robot into a crawling one by simultaneously reconfiguring its magnetization. (scale bar=5 mm) (f) Under a constant low magnetic field (5 mT), a localized spike in temperature triggers a locked reorientation in the leaf (twisting in specific segments), which is retained after the leaf cools, indicating non-volatile memory of the magnetic response. (g) Under a low

magnetic field (5 mT), rapidly heating certain segments induces local deformations that rotate their magnetic domains by ~180°. Under a stronger magnetic field (40 mT), these reprogrammed states cause the leaf to deform into different shapes (scale bar=5 mm). (h) Enabled by thermal reprogramming, a robotic guidewire is designed with reprogrammable magnetic actuation when it "grows" from a catheter. (i) Using a near-infrared (NIR) light-induced reprogramming, the robotic guidewire's magnetic actuation is reprogrammed from an "L" shape to a "U" shape (scale bar=5 mm).

ii) We thank the reviewer for point out problems in figure and figure caption

1. regarding Figure 1, we have revised the caption of Figure 1c to accurately describe the ink constituents rather than the ink formation. For Figure 1f, we have added a schematic illustration to clearly define the axes (axial strain and twist density) and expanded the caption to explain the distinct behaviors labeled i through iv.
2. Regarding Figure 2, we have improved the image clarity in Figure 2a and revised the caption to specify that the images at 100 °C show the filaments in their thermally actuated state. To clarify the relationship between the top view and cross-section: we used the stability of this thermal actuation as a functional indicator of printed cross-sectional symmetry. Specifically, asymmetric cross-sections (caused by low nozzle tilt angles) result in distorted actuation, while $\alpha_t = 50^\circ$ yields stable helical deformation. We have also added the missing legend to Figure 2e. Finally, for Figures 2g and 2h, we updated the captions to explicitly state that the filaments are shown at 100 °C and clarified that all samples began as straight filaments at room temperature."

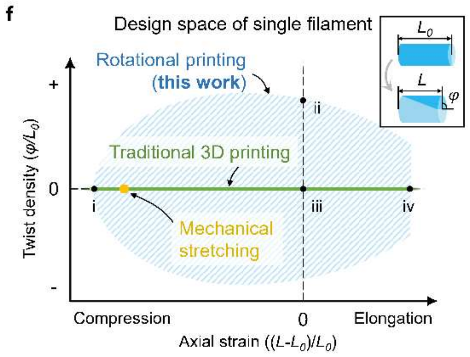

Figure 1. Helix-aligned LCE filament printing system and resulting director orientation and actuation modes. (a) Schematic of the 3D printing setup, featuring a two-axis robotic arm for nozzle positioning, a rotating build platform, and a rotational printhead (with syringe and nozzle) driven by a stepper motor. (b) Illustration of a printed filament showing helical alignment of the LCE director due to nozzle rotation; the director field (**n**) rotates from the core to the periphery, and the angle between the outer-layer director and the filament axis (printing direction, **l**) is defined as θ. (c) Chemical constituents of the LCE ink. (d) Wide-angle X-ray scattering (WAXS) characterization of filament alignment, indicating that the director helix angle θ increases with higher nozzle rotation speed ω. (e) Demonstration of four fundamental thermomechanical deformation modes obtained by programming θ along the filament: axial contraction (0°), near-pure twist (68°), axial elongation (±80°), and zero net deformation (±68°). (scale bar = 0.1 mm) (f) Two-dimensional design space spanned by axial strain and twist density, illustrating the expansion from conventional one-dimensional programming to coupled strain–twist control, where points i–iv correspond to the four deformation modes shown in (e). (g) Multi-modal actuation, time-programmed sequential actuation, and switchable volatile / non-volatile shape memory enabled by different programming forms of θ.

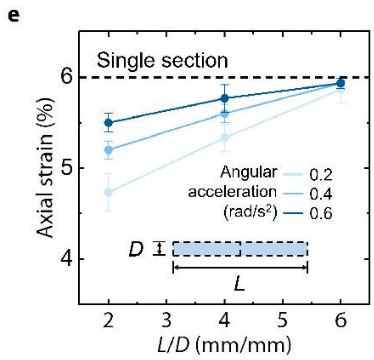

Figure 2. Single filament programming with multi-modality. (a) Comparison of filament morphologies printed at nozzle heights of h^{*}=0.1 mm, 0.2 mm and 0.3 mm, illustrating how overly low or high nozzle heights lead to excessive filament width or zigzag deposition. The bottom panels (100 °C) demonstrate the effect of nozzle tilt angle (α_t) on actuation symmetry, showing that $\alpha_t = 50^\circ$ yields stable helical deformation. (scale bar = 1 mm) (b) Schematic showing how the nozzle's angular speed ω corresponds to the filament helix angle θ, which can be continuously varied from -80° to $+80^\circ$. (c) Influence of different θ values on the LCE filament's twisting (right axis) and axial deformation (left axis) upon heating, transitioning from pure axial contraction to primarily twisting, and eventually to axial elongation. (d) Effect of different angular accelerations on the stability of the printed filament shape, where higher accelerations cause noticeable distortion. (scale bar = 0.5 mm) (e) Axial deformation under different acceleration. (f) The shaded region represents all achievable combinations of axial and torsional deformations (programmable deformation design space), the blue boundary marks the achievable deformation under uniform θ. (g) Selection of target deformations (contraction, zero deformation, twisting, elongation) within the design space by dividing the filament into segments with different θ. (scale bar = 2 mm) (h) Printed samples demonstrating various target shapes achieved via different number of segments. (i) Experimental results show such multiple segmentations programming strategy can precisely program both axial and torsional deformations (Scale bar=5 mm).

Comment #3

i) The mention in the abstract that "programming imposes uniaxial order at voxel level" feels inaccurate or misrepresented here. This point is referenced in many ways throughout the manuscript; the authors emphasize that they are doing some interesting subvoxel alignment. However, I would like to know explicitly how a voxel in this case is defined.

Typically, it relates to the diameter of the strand, which should be close to the diameter of the nozzle. However, it seems based on the video that the authors are significantly overextruding. Can they include in some analysis how the diameter of the strand relates to the diameter of their nozzle, especially at different rotation and print speeds?

ii) I do not see this approach being much different than simple extruding transverse to the direction of a larger printed feature. Additionally, this point does not apply to cases of LCE processing beyond printing (e.g., photopatterning surfaces.)

iii) Typos write away – a little frustrating – "ability to autonomously response".

iv) Similarly, the authors mention that they have many modes of actuation and that typical thermal contraction at the filament level results in "only elementary deformations such as planar folding, arching or twisting." I would like to know what deformations are not elementary since beyond contraction and extension, these seem like pretty complex modes of actuation and additionally omit Gaussian, arbitrary/organic curvature that is possible using antagonistic ordering at interfaces.

v) Additionally, there are previous reports of 3D printed LCE structures that can undergo multiple modes of deformation for a single monolithic print due to asymmetric and/or localized input stimulus (e.g., photoresponse). The responses can be reminiscent to what is reported here and doesn't even require the rotating nozzle.

Response #3

i) We agree that the term "voxel" is often associated with DLP printing or lithographic. For extrusion-based method, although there's paper using voxel or sub-voxel to describe filament-level or sub-filament-level feature (e.g., Natalie et al, Nature 2023), it different from the typical concept of them. To avoid misunderstanding, we replaced these term with filament-level or sub-filament-level in our manuscript.

In addition, to address the reviewer's concern regarding potential overextrusion and to clarify the relevant geometric scale, we added a quantitative calibration of deposited strand width/diameter relative to nozzle diameter (D_f/D_n) across the investigated print speed (v) and rotation speed (ω) space (new Supplementary Fig. 9), and explicitly state how D_f is measured and how this informs the effective filament-level scale used in the manuscript.

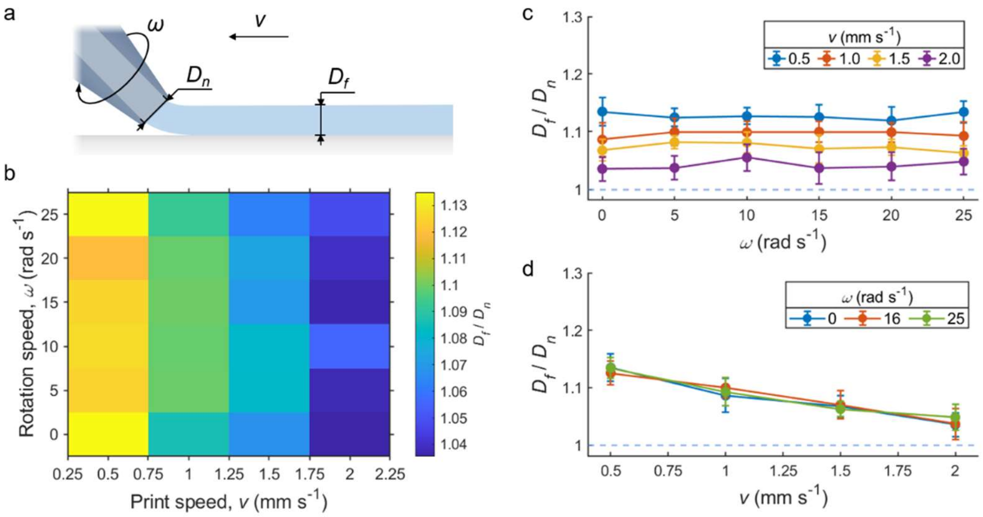

Supplementary Fig. 9. Deposited strand geometry calibration across translation and rotation speeds**.** (a) Definition of nozzle diameter D_n and deposited strand diameter D_f. (b) Map of D_f/D_n across print speed v and rotation speed ω. (c–d) Representative line cuts showing D_f/D_n at different ω and vs. v.

<u>In *Rotational printing enabled helix director field*, we revised:</u>

"While filament diameter—controlled by printing speed at a constant flow rate (Supplementary Fig. 8)—is a critical factor influencing thermal deformation (Supplementary Fig. 9), we maintained a constant diameter in this study; consequently, θ serves as the primary variable governing thermal response."

ii) We thank the reviewer for this comparison. We agree that a feature-scale "transverse" effective alignment can be achieved by raster/zigzag deposition transverse to a part axis. However, this remains path-defined: the local director is still primarily constrained by each deposited filament, so changing actuation relative to a given geometry requires changing the printing trajectory.

In contrast, our rotational printing programs the in-filament helix angle θ through ω/v (or ω at fixed v), thereby largely decoupling the actuation mode from the laydown direction. A further distinction is internal chirality—we generate a helical director field within a continuous filament, enabling torsional and multimodal behaviors that are difficult to reproduce by simple transverse deposition.

We also agree that photopatterning is an important non-printing route, particularly for thin films/surfaces; our discussion here focuses on extrusion-based filamentary/3D architectures. These distinctions are summarized in Supplementary Table 2.

Supplementary Table 2. Comparison of representative LCE programming approaches for controlling director fields and actuation modes.

Approach	Control method	Director field	Couple to geometry / toolpath	Format	Min. feature width	Enabled actuation
Photo - patterning	Polarization pattern, mask	Spatially patterned (mostly planar)	None	thin films	~µm-scale	Rich in-plane deformation; less suited for large or 3D architectures
Mechanical stretching / rubbing	Strain, rubbing direction	Uniform (uniaxial)	Couple with stretching direction	Films / bulk	Sample-scale	Contraction along stretch; limited spatial programming
DIW	Toolpath / degree of alignment	Along filament axis, Piecewise uniform (per printing segment)	High	2D / 3D printed parts	$\approx D_f$ (longitudinal) $\geq 3\ D_f$ (transversal)	Segmental programming, requires complex trajectories to program
Rotational printing (this work)	ω/v (θ)	Helical/chiral within filament	Low	2D / 3D printed parts	$\approx D_f$	Twist / contract / extend from same laydown path; program 3D deformation by θ

iii) Thank you very much, we have thoroughly checked our manuscript to correct typos.
iv) We thank the reviewer for this important point. We agree that folding, arching, and twisting can already represent sophisticated shape changes, and our use of the term "elementary" was imprecise. Our intent was not to downplay these outcomes, but to highlight a programming limitation intrinsic to conventional extrusion-printed LCEs: shear alignment during deposition largely enforces a sign-fixed, uniaxial eigenstrain at the filament level (dominant contraction along the deposition direction), with the deformation mode strongly coupled to the print path. Consequently, realizing more general, non-developable geometries (including surfaces requiring programmed in-plane metrics and Gaussian curvature) typically relies on additional strategies beyond filament-level contraction alone—e.g., curvature-following toolpaths, antagonistic/interface designs, multi-material/voxel-level patterning, or spatially non-uniform stimuli.
We have replaced "elementary" with more precise wording and clarify that our contribution is to expand the intrinsic filament-level actuation space (Fig. 1f and Fig. 2f), enabling both positive and negative axial strain as well as torsion without changing the print path.

In *Introduction*, we revised:

"This constraint largely fixes the filament-level programmed eigenstrain to a dominant axial response along the deposition direction, such that the intrinsic actuation palette of a monolithic extruded filament remains effectively one-dimensional; while sophisticated shape changes (e.g., planar folding[8,30], arching[38,39], and twisting[37,40]) and even richer, non-developable curvatures (including Gaussian curvature) can be achieved via antagonistic/interface designs, voxelation/multi-material architectures, and/or spatially patterned stimuli, these approaches typically add geometric/toolpath or stimulus-field complexity. As a result, the printed LCE structures exhibit strong coupling between deformation mode and printing path, complicating independent control of distinct deformation dimensions."

v) We also agree that prior 3D-printed LCE structures can exhibit multiple deformation modes within a monolithic print when the stimulus field is spatially structured (e.g., localized heating, light absorption gradients, or projected illumination in photoresponsive systems). These reports are highly relevant. However, our approach addresses a different axis of complexity: rather than encoding multimodality in the stimulus distribution at operation, we encode it in the intrinsic director architecture during fabrication using a single parameter (the helix angle θ). As a result, the programmed mode (contraction/extension/torsion/quiescence) and even sequential activation can be executed under uniform, global stimuli (e.g., water bath/oven), as demonstrated by the strain–twist design space (Fig. 1f, Fig. 2f) and the θ-encoded sequential actuation examples (Fig. 4). We will revise the manuscript to explicitly acknowledge stimulus-patterned approaches and position our contribution as fabrication-encoded, stimulus-agnostic programming enabled by rotational printing.

In *Introduction*, we revised:

"Multimodal deformations in a monolithic LCE can also be achieved via spatially structured stimuli, for example locally addressed photo-[44], magneto-[45], or spatially programmed temperature fields. However, the resulting mode is inherently tied to the spatiotemporal stimulus field and may be constrained by practical requirements such as line-of-sight/penetration, spatial calibration, and thermal management, particularly for thick or embedded structures."

Comment #4

Do the grippers actually let go of the object they grip? In the videos, they only show grasping, and it would seem the untangling would be difficult. Furthermore, sequential, multiple instances of pick up and release would be more important in a demonstration of a good gripper. Otherwise, it just could be interpreted as a one time/lucky instance. It seems the authors try to address a concern like this by reported the "grasp success rate". However, it's not clear how this test was performed. Why would the grip success rate have a standard deviation? The only reasonable thing that I can think of is that the success rate was measured for multiple batches of grippers, but that is not mentioned.

Response #4

We have revised Supplementary Movie 5 and added Supplementary Figure 22 to explicitly demonstrate sequential pick-and-place cycles. The video shows the robotic arm grasping an object, moving it to a target location, actively releasing it (detangling), and returning to the initial position to repeat the task.

Regarding the statistics of the "grasp success rate," we clarify that the reported standard deviation represents the variation across multiple experimental sessions. specifically, we conducted 5 independent trials for each case to account for potential variations in setup or object positioning. The mean and standard deviation were calculated based on the success rates of these distinct sessions to provide a more robust statistical evaluation.

In *Method*, we added:
"Data in Figure 4d are presented as mean ± SD based on n=5 independent trials."

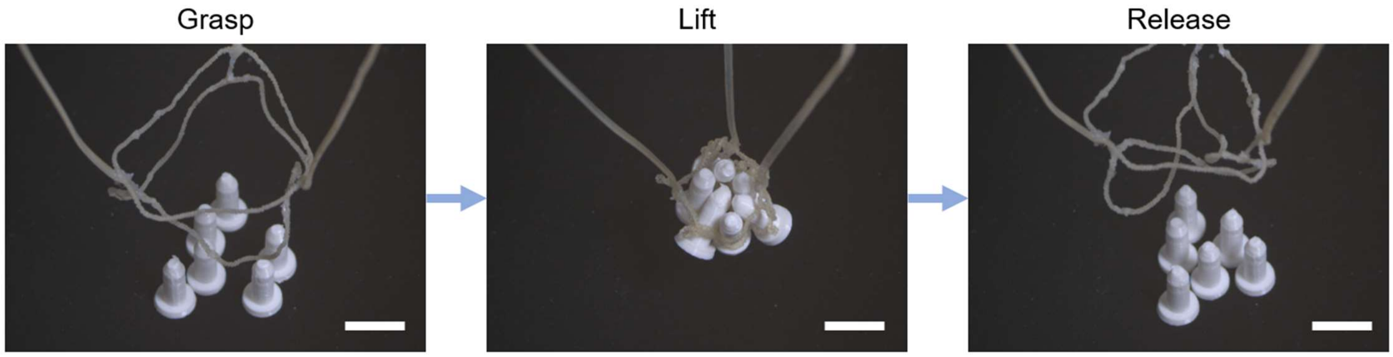

Supplementary Fig. 22. Grasping, lifting, and releasing multiple objects using a self-partitioning gripper (scale bar = 6 mm).

Comment #5
Furthermore for the grippers, the authors show a picture of a robotic arm, but the videos of the gripping look very unstable. Are they actually using the motors to operate the robotic arm, or are they manually picking up objects (either by unlocking the arm and moving it themselves, or by just moving the grippers around by their hand)? If it's the latter, the photos of the robotic arm are misleading. If it's the former, some explanation of the robotic arm or programming that results in shaky/organic movement would be needed.

Response #5
We appreciate the reviewer's keen observation regarding the robotic arm's movement. In our initial proof-of-concept, the robotic arm was temporarily used in a gravity-compensated 'kinesthetic teaching' (lead-through) mode to rapidly validate the gripper's conformal adaptation, which introduced human-hand instability. To address this concern and ensure the rigorousness of our demonstration, we have now fully programmed the robotic arm to execute the picking and placing tasks automatically using its motors. We have re-conducted the experiments and replaced the original videos with new footage. The new videos clearly show the stable, automated operation of the robotic arm, which is now fully consistent with the setup shown in the photos.

Responses to Comments on "NCOMMS-25-85982-T"

We thank the Editor and the reviewers for the positive in-principle decision and for the opportunity to revise the manuscript one final time. In this revision, we have addressed all remaining reviewer concerns and have also revised the manuscript and associated files to comply with the *Nature Communications* formatting requirements and the attached Author Checklist. All corresponding changes are marked in blue in the revised manuscript.

Reviewer # 1

General Comment

The reviewer would like to thank the authors for their careful revision in response to the reviewer comments, specifically the added experimental details and revised language.

Response

Thank you for reviewing our manuscript and the positive comment.

Reviewer # 2

General Comment

The authors did an outstanding job of addressing the critiques and criticisms from reviewers well. This is a large body of excellent work that in my opinion is now at a polished level and accurate level befitting of Nature Communications.

The responses were detailed and thorough and made a big impact on the quality of the work presented.

I support the publication of this work in its current form.

Response

Thank you for reviewing our manuscript and the positive comment.

Code availability

The code is well commented in the G-code portion. There is no README explaining how it should be used and what setup looks like to an untrained user, but this is fairly straightforward for labs with access to compatible printers like the Ultimaker Cura.

The code is nothing special on its own, but provides clarity for groups that might want to build upon the authors' work.

Response

To improve accessibility for non-specialist users, we have added a README to the public Zenodo repository. The README describes the software dependencies, the G-code post-processing workflow, the required printer/hardware setup, and example usage of the scripts. We have also updated the Code Availability statement to reflect this addition.

In *Code Availability*, we revised:

"The custom MATLAB scripts used for G-code toolpath post-processing, together with a README describing software dependencies, workflow instructions, and printer/hardware setup, as well as the CAD design files (.STEP format) necessary to replicate the rotational 3D printing setup, are open-source and available in the public Zenodo repository under the accession DOI: https://doi.org/10.5281/zenodo.18671953."

Reviewer #3

General Comment

I co-reviewed this manuscript with one of the reviewers who provided the listed reports. This is part of the Nature Communications initiative to facilitate training in peer review and to provide appropriate recognition for Early Career Researchers who co-review manuscripts.

Response

We appreciate the reviewer's review and comment.

Reviewer #4

General Comment

Thank you for the response from the authors. My apologies for a hastily written review; it seems I left some typos of my own in my review and left some notes that I intended to clarify. However, the authors appeared to have understood my intention and appeared to have addressed many of the major concerns.

Comment #1

Concerns are adequately addressed

Response #1

We appreciate the reviewer's review and comment.

Comment #2

The authors revised to state, "Embedding complex actuation capabilities into monolithic soft materials remains a challenge for the advancement of soft robotics.", but I don't think that's true. It has been clearly established by various researcher using LCEs and pneumatics that complex actuation is possible. There's really not much value added in that regard that rotational printing enhances.

Response #2

We thank the reviewer for this clarification. We agree that complex actuation has already been demonstrated, and our intent is not to suggest otherwise. We have therefore revised the Abstract to avoid framing the advance as the existence of complex actuation per se. Instead, we now position the contribution more precisely as a strategy for monolithic LCEs that enables multiple modes to be encoded through a single fabrication parameter (θ), while reducing the coupling between actuation mode and laydown path/device geometry. We have also expanded the Discussion to more explicitly delineate the present scope of the method and the future studies needed to extend it beyond the current helical director patterns.

Revised *Abstract*:

"Stimuli-responsive material like liquid crystal elastomers (LCEs) hold great promise for untethered soft machines, yet conventional extrusion-based 3D printing restricts their molecular alignment strictly to the uniaxial deposition path. This inherent constraint strongly couples the actuation mode to the printed geometry, typically requiring complex multi-material architectures or spatially structured stimuli to achieve multimodal behaviors. Here we introduce a rotational 3D printing approach that embeds a helical director field within LCE filaments, enabling multimodal actuation controlled by a single fabrication parameter: the helix angle (θ). Tuning θ programs each filament to contract, elongate, twist or remain macroscopically invariant when heated, decoupling actuation logic from device geometry. Spatial gradients in θ create a hierarchy of activation temperatures, yielding sequential shape changes under uniform heating**.** Localized heating of the magnetic-LCE composite segments allows their magnetic domains to be reoriented, making the shape programs rewritable and enabling switchable volatile and non-volatile memory. We demonstrate these capabilities in self-partitioning grippers, multimodal/color robots and reprogrammable guidewires that perform multi-step or adaptive tasks without external circuitry. By encoding actuation modes, deformation sequences, and memory in a single parameter, this approach establishes a paradigm of material-encoded programmability and points toward monolithic soft robots and reconfigurable structures."

Comment #3
Concerns are adequately addressed

Response #3
We appreciate the reviewer's comment.

Comment #4
The standard deviation was explained well in the response, but the methods within the manuscript could be updated to include a better explanation of grasp success rate and standard deviation. I would think that n = 5 would mean that you had the object picked up 5 times and measured the success rate (e.g., 4/5 = 0.8). But then a standard deviation would involve repeating that test in different instances (resetting the setup/objects as mentioned).

Response #4
We thank the reviewer for pointing out that the previous statistical description was too brief. We have now revised both the Methods and the Fig. 4d caption to explicitly define the grasp success rate and the calculation of the standard deviation. Specifically, for each independent trial, six objects of different shapes were placed beneath the gripper and the setup was reset between trials. The success rate for one trial was calculated as $N_{success}/6$, where a successful grasp was defined as complete lifting and retention of an individual object during the lifting step. The experiment was repeated independently five times for each condition (n = 5), and the data are now reported as mean ± SD across these independent trials.

In *Programmable Sequential deformation for self-partitioning grippers*, we revised:
"Figure 4d quantifies the benefit of this strategy. In tests where the gripper attempted to lift six objects of various shapes at once, the sequential self-partitioning approach produced a much higher success rate than a non-partitioning grasping method. Here, the success rate for a single trial was defined as the fraction of the six objects that were successfully lifted and retained during the lifting step."

In *Method*, we added:
"For the grasping experiments in Fig. 4d, six objects of different shapes were placed beneath the gripper in each trial. A successful grasp for an individual object was defined as complete lifting of the object from the substrate and retention during the lifting step. The success rate for one trial was calculated as $N_{success}/6$, where $N_{success}$ is the number of objects that satisfied this criterion. After each trial, the objects and the gripper were reset to the initial configuration, and the experiment was repeated independently five times for each condition (n = 5)."

Comment #5
While Video 5 appears to have been fixed, video 6 appears to be shaky still. It's probably okay as long as the authors state explicitly. I was also just noting in the methods that it's stated that, "it is integrated with a custom-designed heat gun". That statement appears to reference the gripper, but perhaps the authors could clarify with a photo or description of the heat gun integrated into the robotic arm, if that is what is happening. I imagine that there is no control functionality, so it would be good to explicitly state that the gripper heating is done manually. Also, is Video 5 sped up? There's no indication that it is.

Response #5

We thank the reviewer for this important point. We have clarified the experimental setup in both the main text and the Methods. The gripper is connected to a custom-designed heat gun through a soft connector and adapter and mounted on a 5-axis robotic arm. To avoid overstating the degree of system integration, we now explicitly state that heating was applied in an open-loop manner to trigger the programmed LCE deformation. We have specifically explained in Method that the residual shaking in Supplementary Movie 6 arises from manual handling. We have also revised title of all Supplementary Movies to clarify the play speed of them.

In *Programmable Sequential deformation for self-partitioning grippers*, we revised:

"We fabricated the gripper using an LCE with a low nematic–isotropic phase transition temperature (~18 °C) to minimize heat-induced damage to target objects. The completed device was connected to a custom-designed heat gun through a soft connector and adapter and mounted on a 5-axis robotic arm (Supplementary Fig. 21b, c). In the present proof-of-concept setup, heating was used as an open-loop trigger for the programmed LCE deformation rather than as a closed-loop thermal control system …"

In *Method*, we added:

"*Self- partitioning gripper*. The gripper was printed using LCE with low transition temperature. For heating the self- partitioning gripper, it is integrated with a custom-designed heat gun via a soft connector and adapter, enabling real-time heating of the gripper. Then the gripper is mounted on a 5-axis robotic arm for grasping of objects. While fully pre-programmed movements were utilized in Movie 5, the robotic arm was temporarily operated in a gravity-compensated kinesthetic teaching mode in Movies 6 and 7 to rapidly validate conformal adaptation."

KeyResults

The authors applied a rotational 3D printing technique to control liquid crystal elastomer (LCE) fiber orientation to elicit controlled thermal deformation behavior. Previously, deformation in 3D printed LCEs has been dictated by uniaxial fiber orientation due to the shear experienced in the thixotropic feedstock during extrusion. To overcome this, this work utilizes an extrusion nozzle at which rotates at a prescribed angular velocity, introducing localized radial patterning. Using this technique, the authors modified the nematic - isotropic thermally triggered deformation of the extruded filament. Bulk deformation behavior of filaments can be further controlled by combining multiple sections of discrete fiber orientation angles. Spatially prescribed deformation behavior enabled fabrication of simple 2D lattices with varied deformation modes. Furthermore, the authors fabricated stimuli responsive functional “gripper” with two deformation modes that are triggered at different temperatures. This is accomplished by creating a mismatch in phase transition temperature with varied angles. Lastly, a second method of stimuli responsive deformation was added in the form of magnetic particles.

Validity

Backed by extensive solid mechanics theory. Their method of quantifying “twisting” (phi) leaves something to be desired.

Figure 2:

a. Explain the heating segment of the figure.
b+c. Consider color coding each axis for clarity
d. improve resolution
g. needs a pre-deformation image, indicating where the different rotation zones are. Where are the green and yellow markers you are talking about? The provided figures are not green and yellow. Add scale bar.
h. Same issues as g

The tensile data presented in SI Fig. 9 is inconsistent with LCE literature using the same materials system replicated from other work. Namely, tensile strength is an order of magnitude greater and ultimate strain double that documented in previous literature. If this is indeed the case, then this should be a major focus of the paper, as this is unprecedented performance in LCE materials. However, the Young’s Moduli presented in part d are inconsistent with the supplied tensile data.

Figure SI 10 suggests this technique can achieve impressive actuation strains and work densities versus previous literature, yet it is not referenced in manuscript. The incongruities bring into question the robustness of the experimental approach related to materials characterization.

Significance

Interesting, but unlikely to be sufficiently impactful in the field of LCEs. Rather, this study demonstrates the impactfulness of rotational printing work from the Jennifer Lewis group. On-the-fly modification of properties to spatially tune/program bulk deformation behavior is of great interest in 3D printing. However, this paper does not truly demonstrate 3D printing, as all structures are are single-layered and have no variation in the vertical axis.

Similar rotational DIW:

Larson, N.M., Mueller, J., Chortos, A. *et al.* Rotational multimaterial printing of filaments with subvoxel control. *Nature* **613**, 682–688 (2023). https://doi.org/10.1038/s41586-022-05490-7

Original rotation printing:

J.R. Raney, B.G. Compton, J. Mueller, T.J. Ober, K. Shea, & J.A. Lewis, Rotational 3D printing of damage-tolerant composites with programmable mechanics, Proc. Natl. Acad. Sci. U.S.A. 115 (6) 1198-1203, https://doi.org/10.1073/pnas.1715157115 (2018).

The supercoiling behavior introduced via the rotational deposition is a point of novelty, especially considering the massive improvements in actuation it offers. Regulated coiling behavior is also highlighted in the multi-stage gripper, which is a welcome novel addition to the ubiquitous "gripper" demonstration.

If the tensile results presented in SI fig. 9 are accurate, then this should be a major focus of the paper, as this is unprecedented performance in LCE materials.

Data and Methodology

The presentation of data and demonstrations in the manuscript are of very high quality.

The methods are valid. Indeed, the influence of LCE fiber orientation on deformation of printed LCE structures is well explored. Moreover, spatial mechanical tunability via rotational DIW 3D printing has been successfully demonstrated and documented.

The authors include extensive theoretical discussion of mechanical deformation in viscoelastic cylinders in SI. While this is a welcome justification, this reviewer questions the extent to which this theory was utilized in the design process.

Spatially tunable material behavior is an important aspect of this paper. However, it does not provide sufficient justification of single-filament deformation versus angle (Figure 2g, h). The authors need to considerably strengthen their presentation of how twist and extension was documented including before and after images for angles used in measurements.

The reviewer appreciated the thought put into other demonstrations. Incorporating magnets as a secondary deformation mechanism is quite interesting but ultimately undermines the utility of the rotational printing technique as a viable standalone mechanism for imparting functionality.

The combined phase transition and supercoiling approach to achieving beyond state-of-the-art work densities is impressive. However, it is not clear how they arrived at these results, as methodology is not discussed. It is also unclear what the filament is lifting in b, the authors should specify the weight.

Analytical Approach

The demonstration of bulk "twisting" behavior is quite weak, only really apparent in multi-filament structures.

Suggested improvements

Clarity and Context

The results are clear, and comprehensible by a reader with little experience in LCEs. Ultimately it was an enjoyable paper to read. Unfortunately, the main focus of rotational deposition and controllable LCE deformation lacks novelty and the unbelievable and poorly supported materials characterization data are a major red flag.

References

The author assembled and discussed prior work in LCE actuation thoroughly. However, the authors neglect to reference prior rotational printing work from J. Lewis indicating a surface understanding of the field and perhaps lacking some needed deeper perspective.

Expertise

Detailed evaluation of the analytical discussion in the SI is outside of our expertise.

Tensile analysis

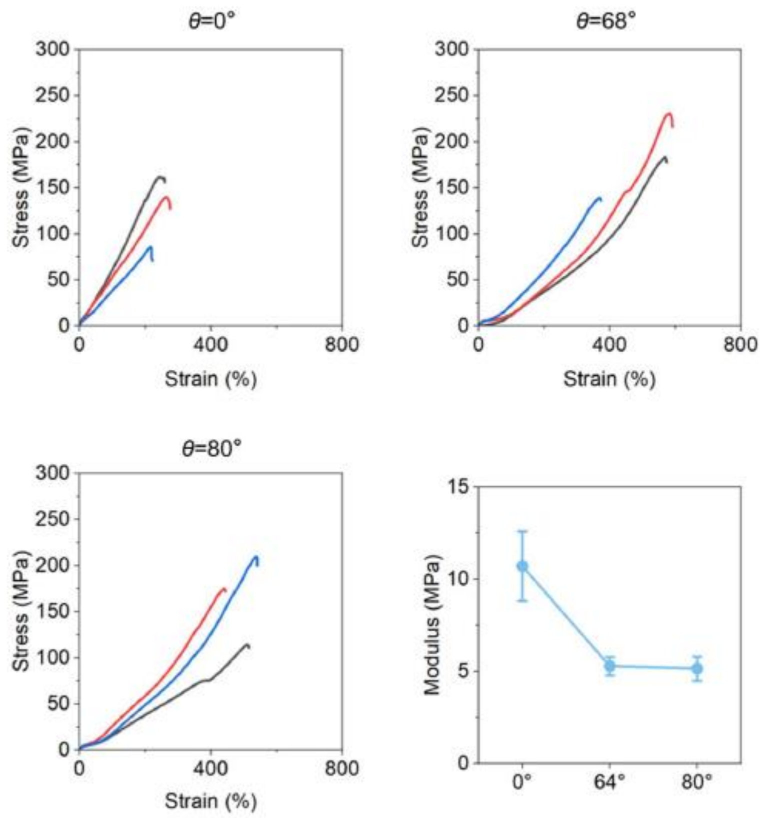

Supplementary Fig. 9. Tensile testing of LCE filaments with different rotational angles indicates that a higher rotational angle leads to a lower tensile modulus.

The printed single filaments were directly used for mechanical tests. Tensile 536 properties were measured on a fiber testing machine with a constant crosshead speed of 10 mm s-

We looked through the supplied tensile data; we could not replicate the Young's Moduli presented through calculations. After analyzing several other papers and speaking with another LCE expert (cited in paper) we believe that this data is flawed of the scale is misrepresented.

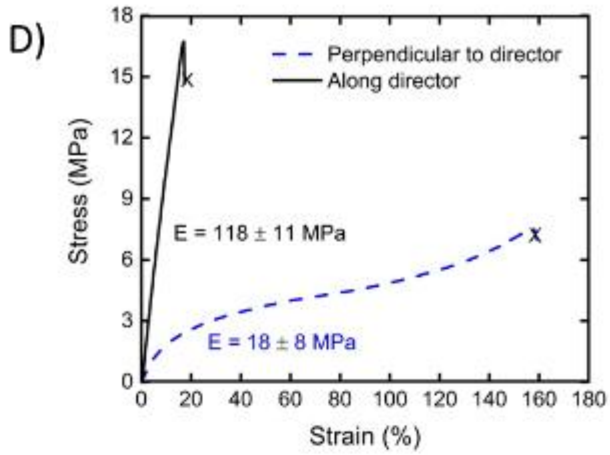

T. Ware paper (**Voxelated liquid crystal elastomers)**
Tensile testing at room temperature of a homogeneously aligned LCE exhibiting characteristic higher modulus along the director than perpendicular to the director.

"Rectangular samples (25 mm x 5 mm x .05 mm) were cut from a homogeneously aligned LCE. The effect of molecular alignment on the engineering stress-strain response was tested by aligning the long axis or short axis of the rectangles along the orientation direction. A strain rate of 0.5 min-1 was used. As seen in Fig. S3D the modulus of the elastomer film is higher along the director than perpendicular to the direction. This is characteristic behavior of an aligned liquid crystalline polymer."

1,4-Bis-[4-(6-acryloyloxyhexyloxy)benzoyloxy]-2-methylbenzene (RM82) and 1,4-Bis-[4-(3-acryloyloxypropyloxy)benzoyloxy]-2-methylbenzene (RM257) were purchased from Merck. The photoalignment material, PAAD-22, was purchased as a solution in dimethylformamide (DMF) from BEAM Co. (USA) and diluted to 1/3 of initial concentration by adding DMF. N-Butylamine was purchased from Sigma Aldrich (USA). The radical photoinitiators, Darocure TPO and I-784, were provided by BASF (Ciba). Unless otherwise mentioned, all chemicals were used as received without further purification or modification.

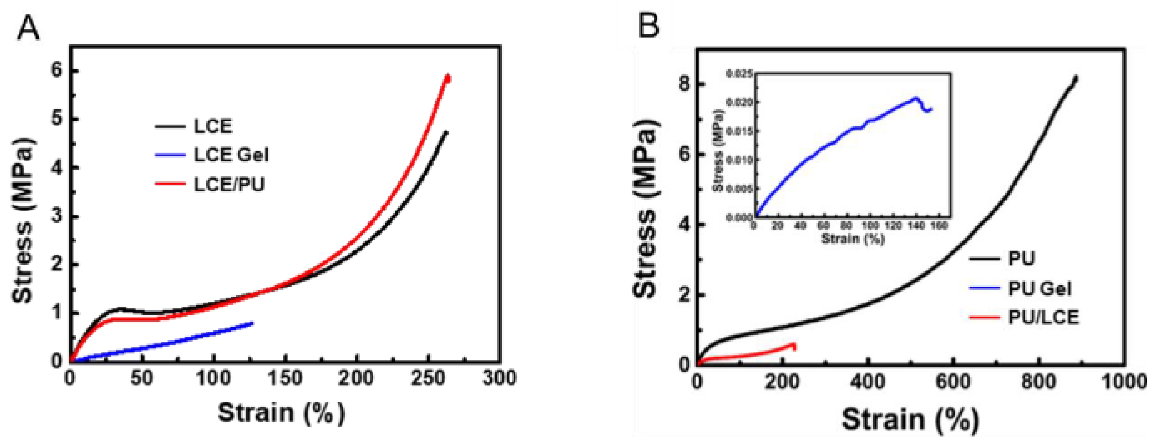

Fig. S3. Stress-strain curves of the first networks, the organic gels, and double network actuators. (A) LCE/PU actuator. (B) PU/LCE actuator.

1,4-Bis[4-(6-acryloyloxyhexyloxy)benzoyloxy]-2-methylbenzene (RM82) was purchased from Bayi Space. 2,2-Dimethoxy-2-phenylacetophenone (DMPA), phenylbis(2,4,6-trimethylbenzoyl)phosphine oxide (photoinitiator 819), and EDDT were obtained from TCI. PU diacrylate (EBECRYL 8413) was acquired from Shanghai Kaiyin. Toluene was purchased from Guoyao Chemical. All chemicals were used as received.

The mechanical performance of LCE film is shown in **Figure S7**.

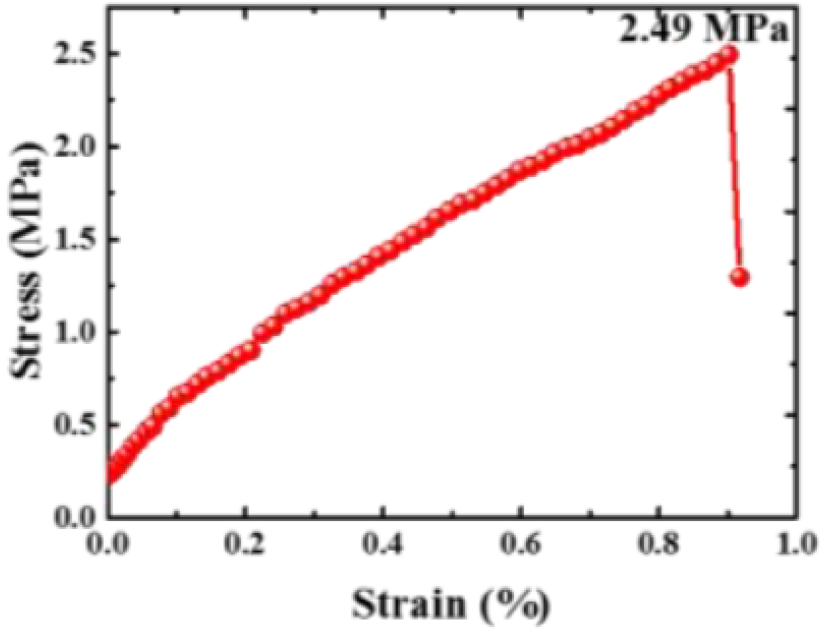

Figure S7. The relationship between strain and stress for LCE film with thickness of 45 μm.
